# Focal adhesion kinase/Src family kinase axis-mediated tyrosine phosphorylation of metabolic enzymes facilitates tumor metastasis

**DOI:** 10.1038/s41392-025-02395-5

**Published:** 2025-09-01

**Authors:** Jie Chen, Jing Zhang, Yuheng Zhu, Yanmeng Zhu, Jingyuan Pang, Qingnan Wu, Yan Wang, Qimin Zhan

**Affiliations:** 1 Key Laboratory of Carcinogenesis and Translational Research (Ministry of Education/Beijing), Laboratory of Molecular Oncology, Peking University Cancer Hospital & Institute, Beijing, China; 2 Peking University International Cancer Institute, Peking University, Beijing, China; 3 Research Unit of Molecular Cancer Research, Chinese Academy of Medical Sciences, Beijing, China; 4 Soochow University Cancer Institute, Suzhou, China; 5 Institute of Cancer Research, Shenzhen Bay Laboratory, Shenzhen, China

**Keywords:** Gastrointestinal cancer, Cancer metabolism

## Abstract

Lymph node metastasis is crucial for esophageal squamous cell carcinoma (ESCC) malignancy. However, the molecular drivers and related mechanisms of lymph node metastasis in ESCC cells are unclear. In the present study, we found that the tyrosine kinase complex-focal adhesion kinase (FAK)/Src family kinase (SFK) axis specifically contributes to metabolic reprogramming by inducing the phosphorylation of ATP-citrate synthase (ACLY) Tyr542, Tyr652, and fructose-bisphosphate aldolase A (ALDOA) Tyr174, Tyr302, or Tyr328 sites in both primary and metastatic ESCC cells. Mechanistically, activated ACLY and ALDOA and their metabolites drive a transcriptional program in primary tumors that induces cyclin-dependent kinase 7/9 (CDK7/9) complex-mediated expression of DNA replication- and cell proliferation-related molecules. This process functions as an enabler of tumor malignancy. In metastatic tumor cells, metabolic enzymes and their products facilitate the transcriptional activity of Yamanaka factors to induce the activation of downstream plasticity-related molecules, fueling ESCC cell survival within metastatic lymph nodes. FAK/SFK axis-controlled ACLY and ALDOA tyrosine phosphorylation and downstream transcription factors and effectors in primary and metastatic ESCC cells are strongly associated with poor patient outcomes. We discovered a lead compound, quercetagitrin, that inhibits the phosphorylation of ALDOA at Tyr174, 302, and 328. Moreover, it has been shown to have antitumor effects alone or in combination with FAK/SFK inhibitors both in vivo and in vitro. The inhibition of tyrosine kinase-regulated metabolic enzyme activities and related signaling networks may be a potential strategy for the treatment and diagnosis of metastatic ESCC.

## Introduction

Esophageal squamous cell carcinoma (ESCC), the prominent histological subtype of esophageal cancer (EC) in China, is characterized by a progressive clinical process and poor prognosis.^1,2^ ESCC is associated with the risk of extensive lymphatic spread, even when the tumor is confined to the submucosa.^3–5^ The occurrence rate of lymphatic metastasis is ~50% in patients with ESCC.^6^ According to previous studies, the detection of lymph node metastasis in patients with ESCC after surgical resection generally indicates an unfavorable prognosis.^7,8^ ESCC is a critical issue and has attracted considerable attention in ESCC treatment.

Tumor cells dissociate from primary sites and then metastasize to distant organs, contributing to the mortality of patients with solid tumors.^9–12^ Surgery and adjuvant therapy can cure some tumors without metastasis. However, metastatic tumors, which are specific to regional lymph nodes, are largely untreatable because of the heterogeneity of these metastatic tumor cells, which are resistant to therapeutic agents and prone to relapse.^13–16^ The underlying molecular mechanisms that drive intratumoral gene reprogramming to induce the lymph node metastasis of tumor cells are not well understood. Various molecular functional approaches have been used to explore critical signaling molecules involved in the lymph node metastasis of tumor cells, and these molecules might be feasible diagnostic and therapeutic targets.^17–20^ Despite the low level of tyrosine phosphorylation in normal cells, it is the core posttranslational modification that, upon deregulation, facilitates the transformation of normal cells and consequently induces the development of malignant phenotypes in tumor cells.^21,22^ Importantly, tyrosine phosphorylation controls signaling networks and represents a mechanism by which tumor cells maintain the activated status of downstream molecules via direct protein‒protein interactions.^23–26^ Thus, tyrosine kinases have emerged as predominant drug targets. Notably, almost all amino acid phosphorylation-targeted drugs clinically used for tumor treatment are tyrosine kinase inhibitors; for example, EGFR inhibitors are used against non-small cell lung cancer.^27–29^

FAK, a nonreceptor tyrosine kinase and hub protein involved in signaling crosstalk, effectively regulates intracellular pathways and cell movement.^30,31^ Importantly, under several stresses, FAK and SFKs form tyrosine kinase complexes that bind to and phosphorylate various adaptor proteins.^30–32^ FAK and SFKs are frequently hyperactivated in solid tumors and are regarded as valuable drug targets.^33–35^ Thus, several FAK and SFK inhibitors are currently being developed and tested in clinical trials. Activated FAK or SFKs can function as addictive oncoproteins to mediate ESCC malignancy, and by targeting FAK and SFKs, the malignancy of such solid tumors can be effectively blocked.^36,37^ However, studies on how FAK and SFKs synergistically mediate the progression and lymph node metastasis of ESCC cells are limited. Importantly, FAK and SFKs are vulnerable targets, and their regulated signaling networks can be exploited for treating metastatic ESCC.

The perturbation of metabolism in tumor cells and their surrounding microenvironment can promote tumor progression, relapse, therapeutic resistance or vulnerability, and other important events related to the clinical outcome of patients with tumors.^38,39^ Specifically, hyperactivation of metabolic enzymes and aberrant accumulation of metabolites can facilitate these events. ACLY (the initiator of fatty acid synthesis) and ALDOA (a critical enzyme for glycolysis and gluconeogenesis) are located at the central linkage of glycolysis and glucose and lipid metabolism.^40,41^ Previous studies have demonstrated that the phosphorylation of serine/threonine residues in metabolic molecules can effectively transduce intracellular signals to mediate several malignant phenotypes of tumor cells.^42,43^ With the development of detection technologies, novel tyrosine phosphorylation sites and their relevant functions have received attention from researchers. Schneider et al reported that tyrosine kinase-ALK binds to and phosphorylates the metabolism-related enzyme GUK1 at the tyrosine 74 (Tyr74) site in the catalytic domain, resulting in increased GDP biosynthesis and facilitating the malignancy of lung cancer cells, indicating that tyrosine phosphorylation in the catalytic domain is critical for the activity of intratumoral metabolic enzymes.^44^ Correspondingly, we selected tyrosine sites, including Tyr174, Tyr302, and Tyr328 in the catalytic domain of ALDOA, Tyr542, and Tyr652 in ACLY for further studies and evaluated whether the highly activated FAK/SFK axis induces the phosphorylation of these specific tyrosine sites in the catalytic domains of these two key fatty acid and glucose metabolic enzymes to promote the lymph node metastasis of tumor cells.

## Results

### Expression of ACLY Tyr542, Tyr652, and ALDOA Tyr174, Tyr302, or Tyr328 in clinical ESCC samples

**Fig. 1:**
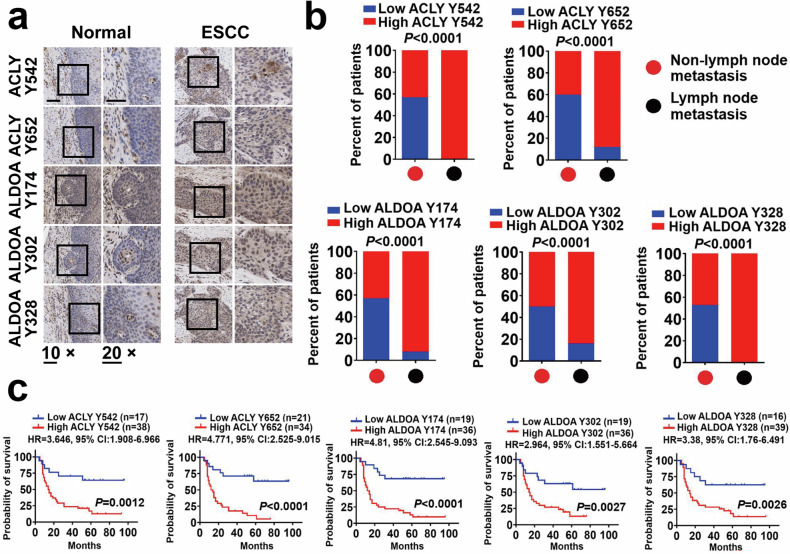
ACLY Tyr542, Tyr652, and ALDOA Tyr174, Tyr302, or Tyr328 are correlated with ESCC malignancy. **a** Representative images of pACLY Tyr542, Tyr652, and pALDOA Tyr174, Tyr302, or Tyr328 in sequential slices of 55 ESCC tumor tissues and 10 normal tissues. Magnification, 10×, 20× as indicated. **b** Percentages of 55 ESCC patients with high or low expression of intratumoral pACLY Tyr542, Tyr652, and pALDOA Tyr174, Tyr302, or Tyr328 according to lymph node metastasis status. Two-tailed Pearson *χ*^2^ test. **c** Survival curves of ESCC patients (55 patients) with low vs high expression of pACLY Tyr542, Tyr652, and pALDOA Tyr174, Tyr302, or Tyr328

Immunohistochemistry (IHC) analysis was performed to evaluate the expression of pACLY Tyr542, Tyr652, and pALDOA Tyr174, Tyr302, or Tyr328 in 55 clinical ESCC samples and 10 adjacent normal tissue samples. Figure 1a shows that these tyrosine proteins were highly expressed in ESCC samples; the expression ratios of pACLY Tyr542, Tyr652, and pALDOA Tyr174, Tyr302, or Tyr328 in ESCC tumors were 69.1%, 38/55 (pACLY Tyr542), 61.8%, 34/55 (pACLY Tyr652), and 65.5%, 36/55 (pALDOA Tyr174 and Tyr302), or 71%, 39/55 (pALDOA Tyr328), respectively. The expression of these tyrosine-phosphorylated proteins was positively correlated with the metastasis of ESCC cells (Fig. 1b). The survival curve revealed that higher expression of tyrosine-phosphorylated ACLY and ALDOA active sites was associated with poorer survival than lower expression (Fig. 1c).

### FAK and SFKs phosphorylate specific tyrosine sites in ACLY and ALDOA in primary or metastatic ESCC cells

**Fig. 2:**
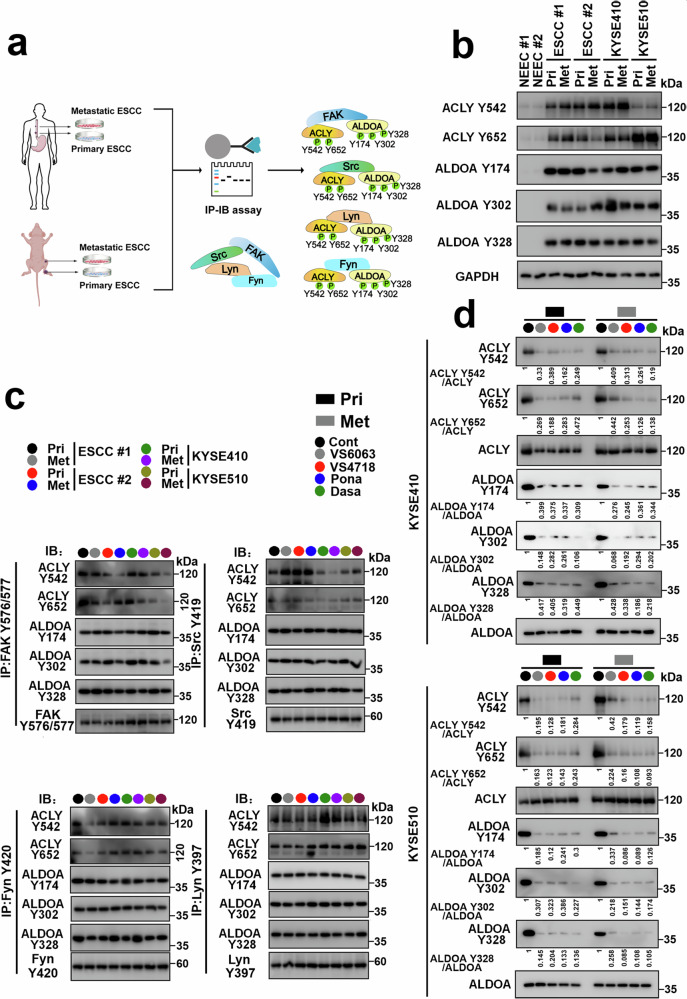
The FAK/SFK axis activates tyrosine phosphorylation of ACLY and ALDOA in primary and metastatic ESCC cells. **a** Schematic overview of the experimental design showing the interaction between the FAK/SFK axis and ACLY or ALDOA and the FAK/SFK axis-induced tyrosine phosphorylation of ACLY and ALDOA in primary and lymph node metastatic ESCC cells. Adobe Illustrator 2022 software was used to construct Fig. 2a. **b** Two cases of primary ESCC cells and paired lymph node metastatic ESCC cells and primary KYSE410 or KYSE510 cells and their paired lymph node metastatic tumor cells from a xenograft mouse model were cultured, and then, cell lysates from the ESCC cells were subjected to immunoblotting, which was used to measure the expression of pACLY Tyr542, Tyr652, and pALDOA Tyr174, Tyr302, or Tyr328. **c** Lysates from ESCC cells were immunoprecipitated with pFAK Tyr576/577, pSrc Tyr419, pLyn Tyr397, or pFyn Tyr420 (IP: pFAK Tyr576/577, left in upper panel; pSrc Tyr419, right in upper panel; pFyn Tyr420, left in lower panel; pLyn Tyr397, right in lower panel). Subsequently, immunocomplexes were immunoblotted using antibodies against the FAK/SFK axis and ACLY Tyr542, Tyr652, and ALDOA Tyr174, Tyr302, or Tyr328 (IB: pFAK Tyr576/577, pSrc Tyr419, pFyn Tyr420, or pLyn Tyr397, pACLY Tyr542, Tyr652, and pALDOA Tyr174, Tyr302, or Tyr328). **d** Primary (black box) and metastatic (gray box) KYSE410 (upper panel) and KYSE510 (lower panel) cells were treated with VS-6063 or VS-4718 (2.5 μM) or ponatinib (5 μM) or dasatinib (0.5 μM), and the expression of pACLY Tyr542, Tyr652, and pALDOA Tyr174, Tyr302, or Tyr328 was assessed by immunoblotting

We applied two research models: (1) cultured ESCC cells from 2 cases of paired primary and lymph node metastatic ESCC cells and (2) established a KYSE410 or KYSE510 lymph node metastasis mouse model and isolated ESCC cells from the primary site and lymph nodes for culture to evaluate the expression of pACLY Tyr542, Tyr652, and pALDOA Tyr174, Tyr302, or Tyr328 in primary and metastatic tumor cells (Fig. 2a). As shown in Fig. 2b, pACLY Tyr542 and Tyr652 and pALDOA Tyr174, Tyr302, and Tyr328 were highly expressed in primary ESCC cells and lymph nodes but not in normal esophageal epithelial cells (NEECs).

Antibodies against pFAK Tyr576/577, pSrc Tyr419, pLyn Tyr397, or pFyn Tyr420 were used to immunoprecipitate the lysates from paired primary and metastatic ESCC cells or KYSE410 and KYSE510 cells from the primary site and lymph nodes, and the expression of pACLY Tyr542 and Tyr652 and pALDOA Tyr174, Tyr302, and Tyr328 in FAK/SFK complexes was evaluated by immunoblotting. As shown in Fig. 2c, the FAK/SFK axis effectively interacted with the ACLY and ALDOA tyrosine sites in both primary and metastatic ESCC cells. Metabolic enzymes mostly exert their catalytic function in the cytoplasm. We evaluated the interaction between the FAK/SFK complex and pACLY or pALDOA in the cytoplasm of ESCC cells. The results from Supplementary Fig. 1a‒d revealed that the FAK/SFK axis interacted with pACLY Tyr542, Tyr652, and pALDOA Tyr174, Tyr302, and Tyr328 in the cytoplasm of ESCC cells.

We then treated ESCC cells with FAK inhibitors, including VS-6063 or VS-4718 (2.5 μM), and SFK inhibitors, such as ponatinib (5 μM) or dasatinib (0.5 μM), and observed that these inhibitors suppressed the expression of pACLY and pALDOA in ESCC cells. As shown in Fig. 2d, FAK/SFK inhibitors effectively suppressed the expression of pACLY Tyr542 and Tyr652 and pALDOA Tyr174, Tyr302, and Tyr328 in primary and metastatic ESCC cells.

Clinically, ESCC tumors were used to evaluate the correlation between the FAK/SFK axis and pACLY and pALDOA. As shown in Supplementary Fig. 2a, b and Supplementary Tables 1 and 2, the expression of pFAK Tyr576/577, pSrc Tyr419, pLyn Tyr397, or pFyn Tyr420 was positively correlated with that of pACLY Tyr542, Tyr652, and pALDOA Tyr174, Tyr302, or Tyr328 in both primary and metastatic ESCC tumors.

### The FAK/SFK axis controls the activity of ACLY and ALDOA in both primary and metastatic ESCC cells

**Fig. 3:**
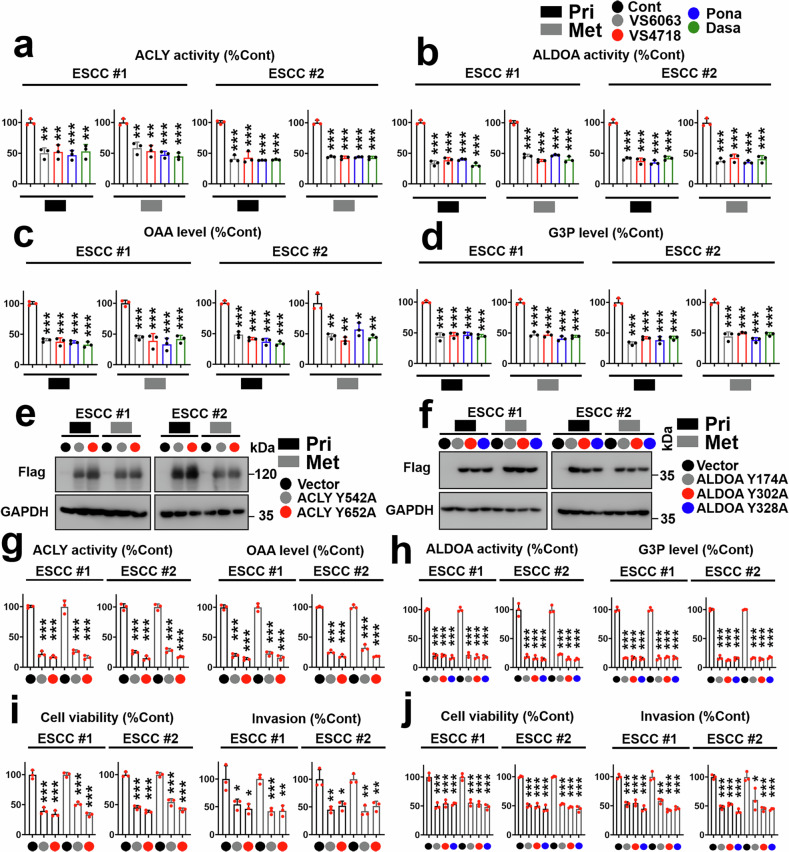
The FAK/SFK axis regulates the activity of ACLY and ALDOA in primary and metastatic ESCC cells. Primary (black box) and metastatic (gray box) ESCC cells were treated with VS-6063 or VS-4718 (2.5 μM), and ponatinib (5 μM), dasatinib (0.5 μM), the activities of ACLY (**a**) and ALDOA (**b**), the production of OAA (**c**), or G3P (**d**) were evaluated (*n* = 3). Transfection efficacy of ACLY Y542A, Y652A (**e**) and ALDOA Y174A, Y302A, or Y328A **f** was evaluated by immunoblotting. The activities of ACLY (left panel of **g**) and ALDOA (left panel of **h**) in primary and metastatic ESCC cells harboring ACLY Y542A, Y652A, and ALDOA Y174A, Y302A, or Y328A were evaluated. The production of OAA (right panel of **g**) or G3P (right panel of **h**) from primary and metastatic ESCC cells harboring ACLY Y542A, Y652A, and ALDOA Y174A, Y302A, or Y328A was measured (*n* = 3). The cell viability and invasive ability of primary (black box) and metastatic (gray box) ESCC cells harboring ACLY Y542A, Y652A (**i**) and ALDOA Y174A, Y302A, or Y328A **j** were assessed via MTS and Matrigel-based transwell assays (*n* = 3). The data are presented as the means ± SDs. *P < 0.05; **P < 0.01; ***P < 0.001

Primary and metastatic ESCC cells were treated with VS-6063, VS-4718 (2.5 μM), ponatinib (5 μM), or dasatinib (0.5 μM), and the enzymatic activities of ACLY and ALDOA and relevant products, including oxaloacetate (OAA, produced by ACLY) and glyceraldehyde 3-phosphate (G3P, produced by ALDOA), were measured. As shown in Fig. 3a‒d and Supplementary Figs. 3a‒d and 4a, b the inhibition of FAK/SFKs effectively blocked the activity of ACLY and ALDOA and the production of OAA, G3P, and pyruvate (an active metabolite in the OAA and G3P pathways) in both primary and metastatic ESCC cells.

ACLY Y542A, Y652A, and ALDOA Y174A, Y302A, and Y328A mutants were transfected into primary and metastatic ESCC cells (Fig. 3e, f), and the activities of ACLY and ALDOA and relevant products were measured (Fig. 3g, h). As shown in Fig. 3g, h, the ACLY Y542A and Y652A mutants blocked the enzymatic activity of ACLY and inhibited the production of OAA in 2 pairs of primary and metastatic ESCC cells. The ALDOA Y174A, Y302A, and Y328A mutants suppressed ALDOA activity and the production of G3P in 2 paired primary and metastatic ESCC cell lines. The effects of ACLY and ALDOA tyrosine mutations on the growth and invasion of ESCC cells were measured via MTS, soft agar, or invasion assays. As shown in Fig. 3i, j and Supplementary Fig. 5a, b, loss-of-function (LOF) mutation of these metabolic enzymes effectively inhibited the malignant progression of primary and metastatic ESCC cells. Depletion of ALDOA or ACLY via small interfering RNAs (siRNAs) inhibited the activity of these enzymes and the production of their respective products (Supplementary Figs. 6a‒c, g‒i). Similarly, ALDOA or ACLY siRNAs inhibited the malignant progression of primary and metastatic ESCC cells (Supplementary Figs. 6d‒f, j‒l). Specifically, we evaluated whether the regulation of the FAK/ACLY/ALDOA axis affects the invasion of KYSE410 and KYSE510 cells. As shown in Supplementary Fig. 7a, b, ACLY Y542A, Y652A, and ALDOA Y174A, Y302A, or Y328A effectively inhibited the invasion of FAK-overexpressing ESCC cells.

### Phosphorylation of critical tyrosine sites in ACLY and ALDOA affects downstream signaling molecules in primary and metastatic ESCC cells

**Fig. 4:**
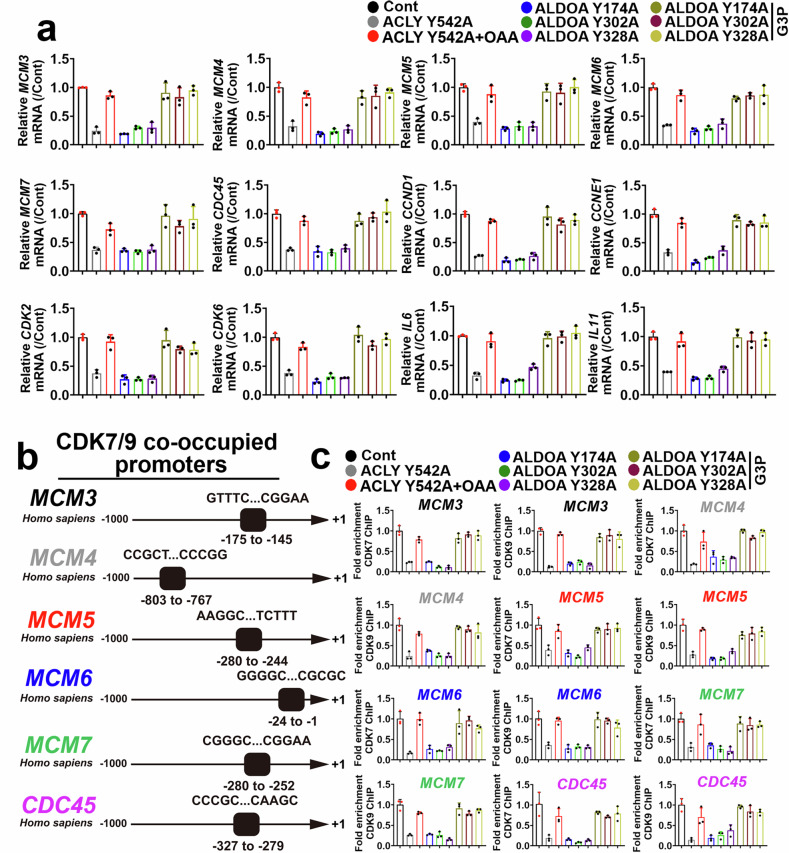
ACLY and ALDOA tyrosine phosphorylation critically contributes to the activation of CDK7/9 and the downstream MCM/CDC45 complex in primary ESCC cells. **a** PCR was used to evaluate the mRNA expression of *MCM3*, *MCM4*, *MCM5*, *MCM6*, *MCM7*, *CDC45*, *CCND1*, *CCNE1*, *CDK2*, *CDK6*, *IL6*, or *IL11* in primary ESCC cells harboring ACLY Y542A with or without OAA (1 μM), ALDOA Y174A, Y302A, or Y328A with or without G3P (1 μM) (*n* = 3). **b** CDK7/9-bound promoter sequences of *MCM3*, *MCM4*, *MCM5*, *MCM6*, *MCM7*, or *CDC45* from -175--145, -803--767, -280--244, -24--1, -280--252, and -327--279 are indicated. **c** ChIP was used to evaluate the binding ability of CDK7/9 to the *MCM3*, *MCM4*, *MCM5*, *MCM6*, *MCM7*, or *CDC45* promoter regions in primary KYSE410 cells harboring ACLY Y542A with or without OAA (1 μM), ALDOA Y174A, Y302A, or Y328A with or without G3P (1 μM) (*n* = 3). The data are presented as the means ± SDs

We assessed the effects of the ALDOA Y174A, Y302A, Y328A, and ACLY Y542A mutants on downstream tumor-promoting and DNA replication molecules, including *MCM3*, *MCM4*, *MCM5*, *MCM6*, *MCM7*, *CDC45*, *IL6*, *IL11*, *CCND1*, *CCNE1*, *CDK2*, and *CDK6*, in primary ESCC cells (Fig. 4a).^45–48^ The levels of these genes were lower in primary ESCC cells transfected with ACLY Y542A than in nontransfected control cells (Fig. 4a). OAA (1 μM) increased the expression of these genes in primary ESCC cells transfected with ACLY Y542A (Fig. 4a).

LOF mutations of ALDOA at the Tyr174, 302, or 328 site effectively blocked the expression of these characteristic genes. Incubation of ESCC cells harboring ALDOA Y174A, Y302A, or Y328A with G3P (1 μM) rescued the expression of these tumor-promoting molecules in primary ESCC cells (Fig. 4a).

We further investigated the transcriptional regulation of *MCM3*, *MCM4*, *MCM5*, *MCM6*, *MCM7*, and *CDC45* by ALDOA and ACLY and their metabolites in primary ESCC cells. We predicted the transcription factors occupying the promoter regions of these proliferation-related genes via the transcription factor database (TFDB) prediction software (http://bioinfo.life.hust.edu.cn/)^49^ and reported that CDK7/9 (the CDK complex) interacted with the −175 to −145 (core sequence: GTTTCCCGCCACCGCGGGGCGCGCAGCGGAA), −803 to −767 (core sequence: CCGCTCCCGCGCGCGTGCGCCCGCTCGGCCCGGACCCGG), −280 to −244 (core sequence: AAGGCGTCCTCCGCGCCCGCCCCTCCGCCATCTTT), −24 to −1 (core sequence: GGGGCGGAAGCGGCGGCGGCGCGCGC), −280 to −252 (core sequence: CGGGCCTGCCCCGCCCTGCGGCCCCGGAA), or −327 to −279 (core sequence: CCCGCCGACCGCTCTCCCAGCCGCCGCTGCCGCTGCCGCCGCGCCGCGCCAAGC) sites in the promoter regions of the *MCM3*, *MCM4*, *MCM5*, *MCM6*, *MCM7*, and *CDC45* genes, respectively (Fig. 4b). Chromatin immunoprecipitation (ChIP) confirmed these predictions and demonstrated that CDK7 and CDK9 co-occupied the promoter region of these genes (Fig. 4c). The results from the ChIP assay further revealed that the regulation of ALDOA and ACLY tyrosine statuses and their products effectively affected the interaction between CDK7/9 and the promoter regions of the *MCM3*, *MCM4*, *MCM5*, *MCM6*, *MCM7*, or *CDC45* genes in primary KYSE410 cells (Fig. 4c). Compared with the control, the ALDOA Y174A, Y302A, or Y328A and ACLY Y542A mutants effectively decreased the expression of CDK7 and CDK9 in KYSE410 cells, whereas the addition of G3P or OAA (1 μM) significantly rescued the levels of CDK7 and CDK9 (Supplementary Fig. 8a, b).

**Fig. 5:**
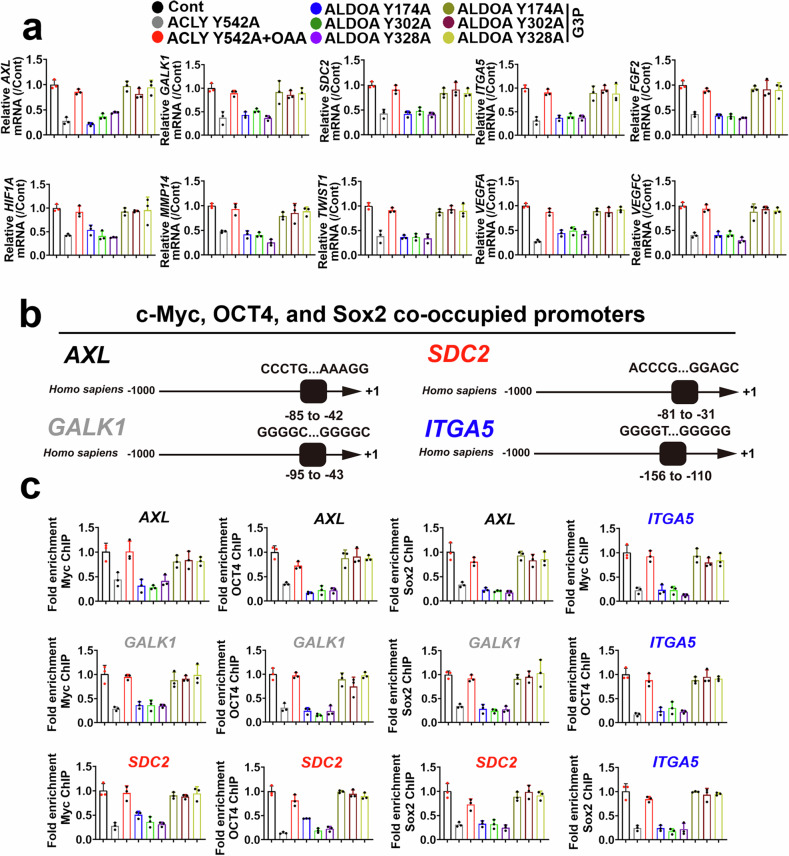
ACLY and ALDOA tyrosine phosphorylation critically contributes to the activation of c-Myc, Oct4, Sox2, and specific downstream molecules. **a** PCR was used to evaluate the expression of the *AXL*, *GALK1*, *SDC2*, *ITGA5*, *FGF2*, *HIF1A*, *MMP14*, *TWIST1*, *VEGFA*, and *VEGFC* genes in metastatic KYSE410 cells harboring ACLY Y542A with or without OAA (1 μM), ALDOA Y174A, Y302A, or Y328A with or without G3P (1 μM) (*n* = 3). **b** c-Myc, Oct4 and Sox2-bound promoter sequences of *AXL*, *GALK1*, *SDC2*, or *ITGA5* from -85--42, -95--43, -81--31, and -156--110 are indicated. **c** ChIP was used to assess the ability of c-Myc, Oct4, and Sox2 to bind to the *AXL*, *GALK1*, *SDC2*, or *ITGA5* promoter regions in metastatic KYSE410 cells harboring ACLY Y542A with or without OAA (1 μM), ALDOA Y174A, Y302A, or Y328A with or without G3P (1 μM) (*n* = 3). The data are presented as the means ± SDs

A previous study focused on lymph node metastatic tumor cells, which are highly enriched in the immune response, epithelial‒mesenchymal transition (EMT), inflammation, or hypoxia pathway-related molecules.^50^ We determined whether the regulation of ALDOA and ACLY activities can affect changes in characteristic genes in metastatic ESCC cells, including *AXL* (immune response and inflammation pathways), *FGF2* and *VEGFC* (EMT), *GALK1* and *SDC2* (hypoxia), *ITGA5* and *MMP14* (EMT and inflammation pathway), *HIF1A* (inflammation), *VEGFA* (hypoxia and EMT), and *TWIST1* (the classic marker of EMT). As shown in Fig. 5a, ALDOA or ACLY tyrosine mutants effectively inhibited the expression of the *AXL*, *GALK1*, *SDC2*, *ITGA5*, *FGF2*, *HIF1A*, *MMP14*, *TWIST1*, *VEGFA*, and *VEGFC* genes in metastatic KYSE410 cells. The incubation of metastatic KYSE410 cells harboring ALDOA or ACLY tyrosine mutants with G3P or OAA (1 μM), respectively, rescued the expression of the above genes (Fig. 5a). Specifically, promoter regions of *AXL*, *GALK1*, *ITGA5*, or *SDC2*-bound transcription factors were predicted, and our results revealed that Yamanaka transcription factors, including c-Myc, Oct4 and Sox2, could interact with DNA sequences from −85 to −42 (core sequence: CCCTGGTGGGCGGAGGCAAAGGGGGAGCCAGGGGCGGAGAAAGG), −95 to −43 (core sequence: GGGGCGGAACCGGCTGAGGTCTGGGGGCGGGGCGTCCGGGCGCGGGGCGGGGC), −156 to −110 (core sequence: GGGGTTGGAGGGGTGCGCCCCCCCCCCACGCCCCTTAGGGGTGGGGG), or −81 to −31 (core sequence: ACCCGGGGAGGGAGGCGCGGCGCGGGAGGAGGAGGGGCGCAGCCGCGGAGC) in the promoter region of the *AXL*, *GALK1*, *ITGA5*, or *SDC2* genes (Fig. 5b). Similarly, mutation of the ACLY and ALDOA tyrosine sites effectively blocked the interaction between c-Myc, Oct4 and Sox2 and the promoter regions of the *AXL*, *GALK1*, *ITGA5*, or *SDC2* genes, and the addition of the metabolites of these metabolic enzymes rescued the ability of c-Myc, Oct4 and Sox2 to bind to the promoter regions of the *AXL*, *GALK1*, *ITGA5*, or *SDC2* genes (Fig. 5c). The results from Supplementary Fig. 9a‒c revealed that mutated ALDOA and ACLY effectively inhibited the transcriptional activity of c-Myc, Oct4 and Sox2 in metastatic KYSE410 cells. G3P or OAA (1 μM) restored the transcriptional activity of these molecules (Supplementary Fig. 9a‒c). We investigated whether c-Myc, Oct4, and Sox2 facilitate the transcriptional activity of *AXL*, *GALK1*, *ITGA5*, or *SDC2* via a dual-luciferase reporter assay. The oligonucleotide sequences containing the c-Myc, Oct4, and Sox2-binding sites in the promoter regions of *AXL*, *GALK1*, *ITGA5*, or *SDC2* and mutants of these genes were inserted into the pGL3-based reporter plasmid (containing firefly luciferase). The wild-type and mutant genes and the control reporter (Renilla) were cotransfected into HEK293T cells. The overexpression of c-Myc, Oct4, and Sox2 dramatically increased the promoter activity of the wild-type but not the *AXL*, *GALK1*, *ITGA5*, or *SDC2* mutants in HEK293T cells (Supplementary Fig. 10a‒e).

### Identification of potential ALDOA inhibitors

**Fig. 6:**
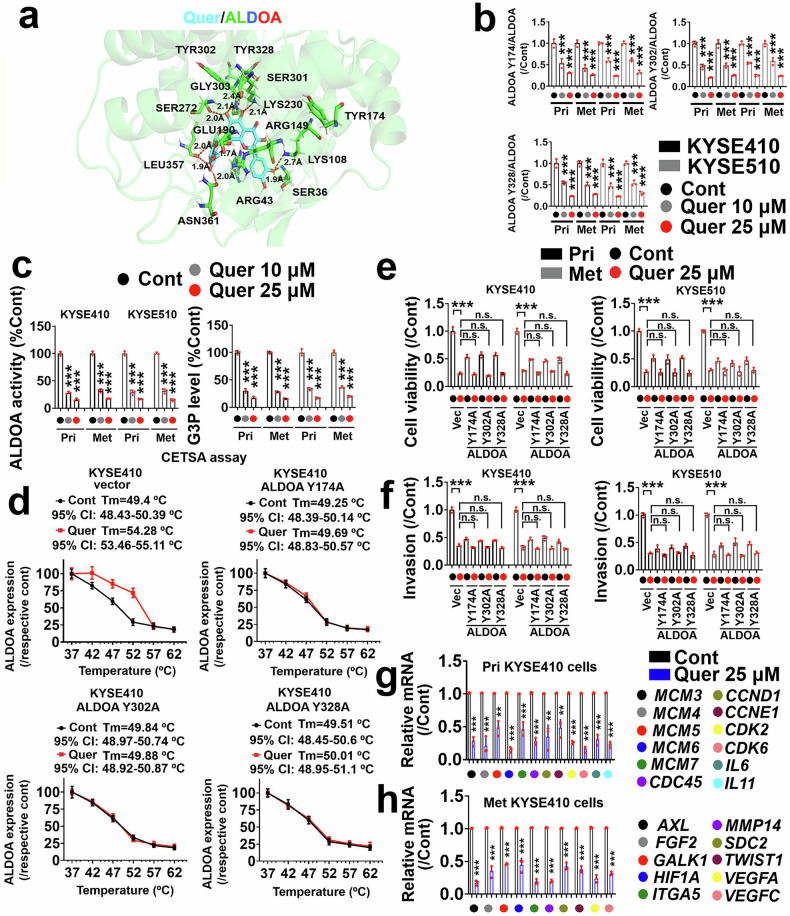
Quercetagitrin inhibits the tyrosine phosphorylation of ALDOA to block ESCC malignancy. **a** Molecular docking between quercetagitrin and ALDOA. Quercetagitrin (0, 10, or 25 μM) dose-dependently inhibited the phosphorylation of ALDOA Tyr174, Tyr302, or Tyr328 **b** and ALDOA activity and G3P production **c** in primary and metastatic KYSE410 and KYSE510 cells (*n* = 3). **d** CETSA assay showing the interaction status between quercetagitrin (50 μM) and ALDOA in KYSE410 cells harboring vector (left of the upper panel in **d**), ALDOA Y174A (right of the upper panel in **d**), Y302A (left of the lower panel in **d**), or Y328A (right of the lower panel in **d**) mutants (*n* = 5). The growth and invasion of ESCC cells incubated with quercetagitrin (25 μM) were assessed via MTS **e** and Matrigel-based transwell invasion assays (*n* = 3) (**f**). **g** PCR assay showing that quercetagitrin (25 μM) inhibited the mRNA expression of *MCM3*, *MCM4*, *MCM5*, *MCM6*, *MCM7*, *CDC45*, *CCND1*, *CCNE1*, *CDK2*, *CDK6*, *IL6*, and *IL11* in primary KYSE410 cells (*n* = 3). **h** PCR assay showing that quercetagitrin (25 μM) inhibited the mRNA expression of *AXL*, *FGF2*, *GALK1*, *HIF1A*, *ITGA5*, *MMP14*, *SDC2*, *TWIST1*, *VEGFA*, and *VEGFC* in metastatic KYSE410 cells (*n* = 3). The data are presented as the means ± SDs. n.s., not significantly different; **P < 0.01; ***P < 0.001

We retrieved the human ALDOA protein (PDB ID: 2ALD) from the RCSB PDB database and applied pockets on the basis of the key residues near Tyr174/302/328 (Fig. 6a and Supplementary Fig. 11). We subsequently docked compounds from the MCE bioactive compound library to select high-affinity compounds for further functional assays (Supplementary Fig. 12a‒h, Supplementary Fig. 13a‒h, and Supplementary Table 3). Quercetagitrin was chosen for further studies (Supplementary Fig. 11), and our results revealed that this compound (10, 25 μM) dose-dependently inhibited the phosphorylation of ALDOA Tyr174, 302, or 328 and ALDOA activity and G3P production in primary and metastatic KYSE410 and KYSE510 cells (Fig. 6b, c). A cellular thermal shift assay (CETSA) was used to evaluate the interaction between quercetagitrin and ALDOA, and Fig. 6d shows that quercetagitrin (50 μM) bound to ALDOA. Furthermore, the ALDOA Y174A, Y302A, and Y328A mutants impaired physical interactions between quercetagitrin (50 μM) and ALDOA in KYSE410 cells (Fig. 6d). The results from the streptavidin pull-down assay followed by electrophoresis analysis revealed that ALDOA was pulled down by biotin-quercetagitrin but not by biotin. Importantly, the biotin-quercetagitrin/ALDOA interaction was decreased in ESCC cells harboring ALDOA Y174A, Y302A, and Y328A mutants (Supplementary Fig. 14). As shown in Supplementary Fig. 15a‒c, quercetagitrin dose-dependently inhibited the activity of the recombinant human ALDOA protein in an in vitro system. Quercetagitrin (25 μM) effectively inhibited the growth and invasion of primary and metastatic KYSE410 and KYSE510 cells but did not further inhibit the malignant progression of these ESCC cells harboring ALDOA Y174A, Y302A, or Y328A mutants (Fig. 6e, f).

Importantly, quercetagitrin (25 μM) effectively inhibited the expression of *MCM3*, *MCM4*, *MCM5*, *MCM6*, *MCM7*, *CDC45*, *CCND1*, *CCNE1*, *CDK2*, *CDK6*, *IL6*, and *IL11* in primary KYSE410 cells (Fig. 6g) and decreased the levels of *AXL*, *FGF2*, *GALK1*, *HIF1A*, *ITGA5*, *MMP14*, *SDC2*, *TWIST1*, *VEGFA*, and *VEGFC* in metastatic KYSE410 cells (Fig. 6h).

### Targeting ALDOA Y174/302/328 sites with quercetagitrin inhibits the malignant progression of ESCC cells in a xenograft mouse model

**Fig. 7:**
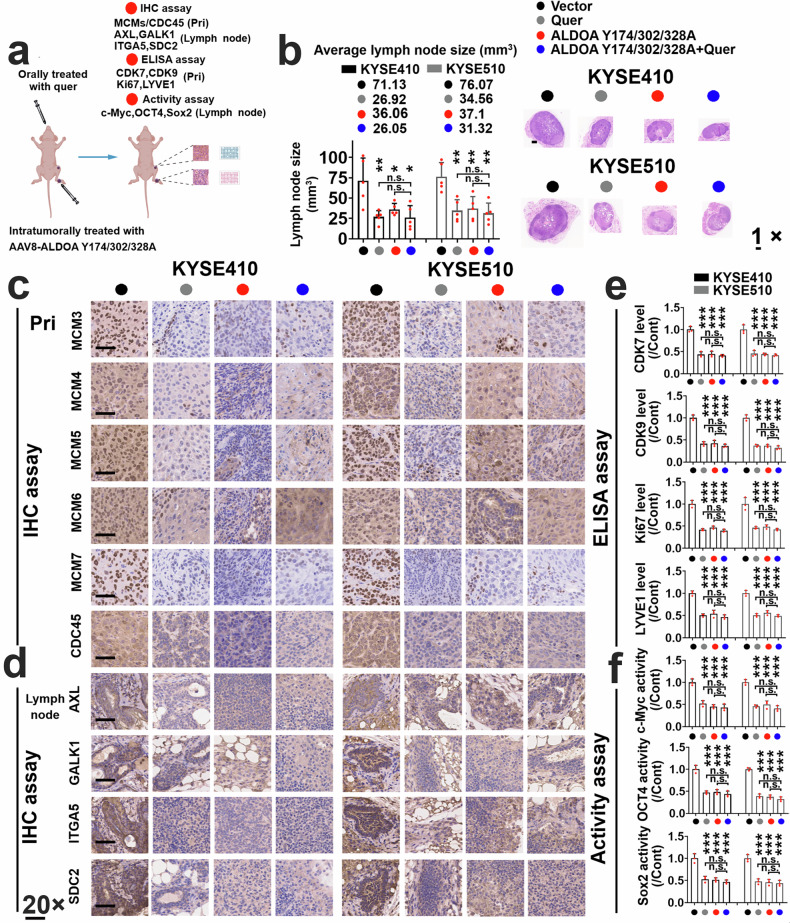
Quercetagitrin inhibits the lymph node metastasis of ESCC cells by blocking ALDOA activity. **a** Protocol used to establish the xenograft model. A lymph node metastasis model was used in the present study. Adobe Illustrator 2022 software was used to construct Fig. 7a. **b** After the footpads were inoculated with KYSE410 or KYSE510 cells for ~1 week, the mice were intratumorally injected with AAV8-CMV-ALDOA Y174/302/328 A into primary sites and orally treated with quercetagitrin (*n* = 5; 25 mg/kg/day, p.o.). After treatment, the lymph nodes were extracted, and the volumes of the lymph nodes were calculated and quantified. Representative images of H&E-stained lymph nodes are shown. Magnification, 1× as indicated. Primary tumors (**c**) or lymph nodes (**d**) from the KYSE410 and KYSE510 tumor groups were extracted, and an IHC assay was subsequently used to evaluate the expression of MCM3, MCM4, MCM5, MCM6, MCM7, or CDC45 in primary tumors (**c**) and the expression of AXL, GALK1, ITGA5, or SDC2 in lymph nodes (**d**). Representative images of IHC staining of the indicated proteins in primary sites or lymph nodes from the KYSE410 (left panel) or KYSE510 (right panel) tumor groups are displayed. Magnification, 20× as indicated. **e** Primary ESCC tumors were lysed, protein lysates were prepared, and the expression of CDK7, CDK9, Ki67, or LYVE1 was evaluated via quantitative ELISA (*n* = 3). **f** Lymph nodes were lysed, and nuclear proteins were obtained and subjected to the transcriptional activity of c-Myc, Oct4, and Sox2 (*n* = 3). The data are presented as the means ± SDs. n.s. not significantly different; *P < 0.05; **P < 0.01; ***P < 0.001

To evaluate whether targeting the ALDOA Tyr174/302/328 sites inhibited ESCC metastasis, we inoculated KYSE410 and KYSE510 cells into the footpad of mice. After ~1 week, the animals were intratumorally injected with AAV8-ALDOA Y174/302/328 A or orally treated with quercetagitrin alone or in combination for 5 weeks (Fig. 7a). As shown in Fig. 7b, AAV8-ALDOA Y174/302/328A or quercetagitrin alone effectively decreased the volume of the lymph nodes of KYSE410 and KYSE510 cells, whereas quercetagitrin did not further increase the effect of AAV8-ALDOA Y174/302/328A on lymph node volume shrinkage. Importantly, quercetagitrin and AAV8-ALDOA Y174/302/328A inhibited the expression of MCMs/CDC45 (as evaluated via IHC; Fig. 7c, and Supplementary Fig. 16a‒f); CDK7, CDK9, and Ki67 (proliferation markers); and LYVE1 (lymphangiogenesis markers; as evaluated via ELISA; Fig. 7e) in primary KYSE410 and KYSE510 tumors and decreased the levels of AXL, ITGA5, GALK1, and SDC2 (as evaluated via IHC; Fig. 7d, and Supplementary Fig. 17a‒d) and the transcriptional activity of c-Myc, Oct4, and Sox2 (Fig. 7f) in the lymph nodes. Specifically, quercetagitrin did not further inhibit the CDK7/9/MCMs/CDC45 signaling axis in primary tumors or Yamanaka transcription factor activity or AXL, ITGA5, GALK1, and SDC2 expression in the lymph nodes of AAV8-ALDOA Y174/302/328A-treated animals (Fig. 7c‒f and Supplementary Figs. 16a‒f and 17a‒d).

To evaluate whether ALDOA tyrosine sites contribute to quercetagitrin-mediated antitumor activity. KYSE410 or KYSE510 cells harboring ALDOA Y174A, Y302A, or Y328A were inoculated into the flanks of the mice. After the tumor volume reached ~100 mm^3^, the mice were treated with quercetagitrin (25 mg/kg/day, p.o.) for 3 weeks. As shown in Supplementary Fig. 18a‒d, quercetagitrin and the ALDOA Y174A, Y302A, or Y328A mutation inhibited the growth of KYSE410 or KYSE510 tumors. Quercetagitrin did not further decrease the tumor volume in ESCC tumors harboring ALDOA Y174A, Y302A, or Y328A mutations. As shown in Supplementary Fig. 18e‒h, quercetagitrin and ALDOA Y174/302/328A mutants effectively decreased the expression of Ki67 and LYVE-1 in tumor tissues; specifically, quercetagitrin could not further reduce the levels of these biomarkers in ESCC tumors harboring ALDOA Y174/302/328A mutants. Similarly, quercetagitrin effectively decreased the G3P level in KYSE410 and KYSE510 tumors (Supplementary Fig. 19). H&E analysis of the intestine, liver, spleen, kidney, and heart from control and quercetagitrin-treated tumor-bearing mice revealed that the morphologies of these organs were not significantly altered between the control and quercetagitrin-treated groups (Supplementary Fig. 20a, b).

### Combining FAK/SFK inhibitors and quercetagitrin synergistically blocked the lymph node metastasis of ESCC cells and downstream effectors

We further assessed whether quercetagitrin could enhance the antitumor effects of FAK and SFK inhibitors in a footpad xenograft model. As shown in Supplementary Fig. 21a, quercetagitrin combined with defactinib (15 mg/kg/day, p.o.) or ponatinib (10 mg/kg/day, p.o.) decreased the lymph node volume in KYSE410 and KYSE510 cells more effectively than defactinib or ponatinib alone. Similarly, quercetagitrin enhanced the inhibitory effects of defactinib and ponatinib on the expression of CDK7/9, Ki67, and LYVE1 in primary tumor tissues (Supplementary Fig. 21b) and the activation and expression of Yamanaka transcription factors in lymph nodes (Supplementary Fig. 21c).

### The FAK/SFK/ACLY Y542/652 and ALDOA Y174/302/328 axes were clinically correlated with downstream effectors in both primary and lymph node metastatic ESCC cells

We evaluated the correlations between pFAK Tyr576/577, pSrc Tyr419, pLyn Tyr397, or pFyn Tyr420 and pACLY Tyr542, Tyr652, and pALDOA Tyr174, Tyr302, and Tyr328 in primary ESCC tumors. As shown in Supplementary Fig. 22a, b and Supplementary Table 4, the tyrosine-phosphorylated FAK/SFK/ACLY and ALDOA axes were positively correlated with MCM3, MCM4, MCM5, MCM6, MCM7, CDC45, CCND1, CCNE1, CDK7, and CDK9 in ESCC tumor tissues.

We further assessed the correlation between the levels of tyrosine-phosphorylated FAK/SFKs/ACLY and ALDOA and those of SDC2, ITGA5, GALK1, FGF2, AXL, MMP14, TWIST1, c-Myc, Sox2, and Oct4 in metastatic lymph nodes. As shown in Supplementary Fig. 23a, b and Supplementary Table 5, tyrosine-phosphorylated proteins of the FAK/SFK/ACLY and ALDOA axes were positively correlated with the indicated downstream proteins in metastatic lymph nodes.

## Discussion

Tumor cells educate themselves to adapt to their surrounding microenvironment and then to facilitate progression and develop several malignant phenotypes. FAK and its interacting proteins, SFKs, can induce the reprogramming of downstream signaling networks to facilitate this activity. Our results showed that the FAK/SFK axis interacts with some metabolism-related molecules and mediates the tyrosine phosphorylation of these molecules to promote the survival of tumor cells. This process involves both primary and lymph node metastatic tumor cells by regulating different downstream signaling networks, indicating that tumor cells preferentially utilize driver oncoproteins to ensure their survival in different lesions. Metabolic reprogramming has been identified as a hallmark of tumors because this biological activity plays a central role in mediating tumor malignancy.^51,52^ However, the molecular mechanisms underlying these intratumoral metabolic switches and the resulting tumor phenotypes remain unclear. Previous studies have confirmed that serine/threonine phosphorylation of metabolic molecules can increase the metabolic rate and facilitate malignant tumor progression, indicating that phosphorylation is critical for the activation of metabolic enzymes and the enhancement of tumor cell metabolism.^42,43,53,54^ Similarly, we reported that FAK and SFKs mediate the tyrosine phosphorylation of ALDOA and ACLY, thereby increasing the activity of these metabolic enzymes. These results suggest that overactivated tyrosine kinases facilitate metabolic reprogramming by directly stimulating the phosphorylation of these key metabolic enzymes. Importantly, our findings suggest that a major aspect of FAK and its partner SFKs in cancer is their role in convergently activating metabolic enzymes and facilitating their activity. Therefore, blockade of metabolic enzyme activities may contribute to a more pronounced suppression of tumor malignancy and the expression of related downstream molecules. However, as FAK, Src, and other SFKs also facilitate metabolism or other biological events, FAK or SFK inhibition alone may fail to result in severe tumor growth inhibition in preclinical or clinical studies.^30,55^ Many approved antitumor agents, particularly tyrosine kinase inhibitors (TKIs) and antibody‒drug conjugates (ADCs), can block primary tumor proliferation; however, they have limited inhibitory effects on metastatic tumors and consequently induce drug resistance.^56–58^ The development of effective antimetastatic therapies remains a critical challenge in oncology.^59^ In the present study, we demonstrated that quercetagitrin uniquely inhibits malignant progression at both primary and metastatic ESCC sites by targeting the hyperphosphorylation of ALDOA tyrosine sites. Our findings provide a promising research strategy for developing novel metastasis-targeted antitumor agents.

Exploring the effects of metabolites on the activation of downstream signaling proteins, especially transcription factors and chromatin-level events, presents significant challenges. Taurine inhibits STAT3 phosphorylation and ATF4 transcriptional activity and consequently suppresses the expression of exhaustion markers in CD8^+^ T cells.^60^ Itaconate activates the intratumoral NRF2‒SLC7A11 pathway to promote immune-induced ferroptosis.^61^ Supplementation with nucleosides can impair the degree of radiation-induced DNA damage and tumor cytotoxicity by suppressing γH2AX activation.^62^ The exact effects of metabolites on signaling pathways are underexplored because of the limitations of methodologies used in scientific research. Specifically, some metabolic enzymes can be phosphorylated to translocate into the nucleus and are involved in the activity of the transcriptional complex. This biological function may be independent of the catalytic function of metabolic molecules.^63–65^ However, how metabolic enzymes affect the assembly of transcription factors is still a major problem in molecular biology and biochemistry. Thus, exploring the effects of metabolites on the activation of downstream transcription factors and chromatin-level events presents significant challenges. Other studies have focused mostly on tyrosine phosphorylation enhancing the enzymatic activity of metabolic molecules.^44,66,67^ In the present study, we demonstrated that the phosphorylation of specific tyrosines in ACLY and ALDOA effectively enhances the enzymatic activity of these two proteins to produce OAA and G3P, which effectively facilitates the function of CDK7/9 in primary ESCC cells and c-Myc, Sox2, and Oct4 in metastatic ESCC cells. We identified a novel biological function of OAA and G3P in regulating transcriptional events in both primary and metastatic tumor cells.

Mutations in the *TP53* tumor suppressor have profound effects on tumor genomic structure, gene expression profiles, and clinical outcomes, especially in ESCC, according to previous studies.^46,68–70^ In particular, many KYSE series ESCC cells, including KYSE410 and KYSE510 cells, harbor *TP53* mutations.^71^ While small molecules that restore mutant p53 tumor-suppressor activity are being explored as potential therapeutic agents, the clinical efficacy of these p53 activators remains controversial. Targeted mutant p53 degradation might also be achieved by taking advantage of a recently discovered critical mechanism underlying mutant p53 accumulation in cancer. Some studies have identified functional molecules with mutant p53.^72–76^ Although our present study revealed that inhibiting ACLY and ALDOA activities effectively blocks the malignancy of *TP53*-mutant ESCC cells, further functional assays and mechanistic investigations are needed to determine whether these two metabolic enzymes can serve as synthetic lethal targets for treating *TP53*-mutated ESCC cells. Studies have established the correlation between protein expression and the mutation status of this molecule at the genetic level. Clinically, somatically mutated genes usually result in the overexpression of the encoded protein.^77,78^ However, determining whether genetic mutations resulting from specific tyrosine phosphorylation in some signaling proteins are interesting topics in the field of cancer research. If we find a highly expressed phosphorylated protein and establish a correlation between its phosphorylation status and genetic mutation, we will provide a novel strategy for tumor diagnosis.

FAK and SFKs can interact with metabolic enzymes or other tumor-promoting proteins. Owing to the excessively complex signaling network involved, the activity of many functionally diverse pathways is regulated by the FAK/SFK axis in primary tumors and metastatic lymph nodes. These diverse pathways fundamentally differ from the metabolite program activated by the FAK/SFK/metabolite axis at primary and metastatic sites. The tumor signaling kinase/metabolite axis supports the malignancy of tumor cells in a pleiotropic manner by directly affecting the cell cycle, DNA replication, anabolic metabolism, and malignant proliferation. In particular, metabolites facilitate the expression of the MCM/CDC45 complex in tumor cells, which contributes to their malignant growth and simultaneously induces the expression of metastasis-related genes. These findings suggest that metabolites in primary tumor cells ensure the maintenance of intratumoral transcriptional programs in highly proliferative tumor cells. Cyclin-dependent kinases, such as CDK7 and CDK9, are functionally connected to the processes of transcription initiation and elongation in various tumors and facilitate the malignant proliferation of tumor cells.^79–81^ In the present study, we determined the regulatory pattern of CDKs in downstream tumor proliferation-related molecules and elucidated the biological mode of uncontrolled growth of primary tumor cells. FAK- and SFK-controlled metabolites and related pathways in metastatic lymph nodes may be beneficial to malignant genes within metastatic tumor cells. A previous study demonstrated that some EMT-like cell subtypes associated with inflammation, the immune response, and hypoxia-related pathways are hyperactivated in metastatic lymph nodes.^50^ The same tyrosine kinase is overactivated in tumor cells and other cellular components surrounding tumor cells, indicating that the activation of tyrosine proteins is the most effective or economical strategy for regulating the transduction of intracellular signals and initiating or maintaining pivotal cellular phenotypes.^82–85^ However, the survival conditions of primary tumor cells and those in metastatic lymph nodes are different. To ensure better survival of tumor cells in different microenvironments, tyrosine phosphorylation-activated signaling proteins differentially regulate downstream effectors to some extent. In support of this perspective, our results showed that FAK/SFK/metabolic proteins stimulate the transcriptional activity of the CDK7/9 complex in primary tumor cells while activating Yamanaka transcription factors to induce the expression of cell plasticity-related molecules in metastatic lymph nodes. Other important factors, including secreted cytokines, chemokines, metabolic products, or even exosomes, may support the survival, development, or other biologic phenotypes of tumor cells in the metastatic region to fuel the malignant state.^50,86,87^ Thus, in primary tumor regions and metastatic lymph nodes, tumor cells must respond to potentially lethal challenges. Tyrosine phosphorylation is sufficient to mediate highly heterogeneous programs of transcriptional activity and their controlled signaling networks, depending on the divergent cellular context. The complementary but distinct functions of signaling pathway-regulated metabolites in primary and metastatic tumors have significant clinical implications. Assessing the tyrosine phosphorylation of FAK/SFKs and their activated metabolites might help determine the malignant potential of ESCC in addition to common clinical parameters. The downstream molecules differentially regulated by the FAK/SFK/metabolite axis can be comprehensively applied to evaluate the development and progression of tumors. Importantly, the dependence of DNA replication or proliferation pathways in supporting tumor cell malignancy, along with several mesenchyme-related pathways contributing to the survival of tumor cells in metastatic nodes, makes FAK-activated metabolites attractive targets for intervention in both tumors in primary and metastatic nodes.

The pharmacological inhibition of kinase hyperphosphorylation and the resulting development of clinically feasible tyrosine kinase inhibitors represent effective strategies for tumor treatment. By integrating molecular docking, functional screening, and validation assays, and biochemical assays, the lead compound quercetagitrin was identified. It directly targets the tyrosine sites in ALDOA, effectively blocking metabolism in both primary and metastatic tumor cells and inhibiting the expression of specific genes in tumor cells. These findings on the role of the tyrosine phosphorylation of ALDOA increase the knowledge of the activity of previously annotated metabolic enzymes and open a distinctive therapeutic avenue for preclinical studies. Despite the limited therapeutic effects of FAK or SFK inhibitors as monotherapies, the FAK/SFK axis is evidently a critical signaling pathway through which tumor cells recover malignant phenotypes following treatment with other antitumor agents.^30^ Therefore, FAK and SFKs are potential targets for combination therapy. In this study, we found that defactinib or ponatinib combined with an ALDOA inhibitor effectively blocked the lymph node metastasis of ESCC cells and related signaling networks. These results will help guide the optimal application of FAK and SFK inhibitors in clinical settings (Supplementary Fig. 24).

## Methods and materials

### Ethics approval statement

All animal procedures were approved by the Institutional Review Board of Peking University Cancer Hospital & Institute (EAEC 2022-06).

All procedures for ESCC sample preparation were confirmed by the Institutional Review Board of Peking University Cancer Hospital & Institute (2020KT117). Written informed consent was obtained from the participants in the study.

### Antibodies

The FAK Tyr576/577 antibody (Cat # 3281) used in the IP and immunoblotting assays and the Sox2 antibody (Cat # 2748 used in the ChIP assay; Cat # 14962 used in the IHC assay) were obtained from CST. The FAK Tyr576/577 antibody (Cat # PA5-37706) used in the IHC assay was from Invitrogen. The Src Tyr419 antibody was obtained from Raybiotech (Cat # 102-17936). Lyn Tyr397 antibody (Cat # ab226778), CDK7 antibody (Cat # ab256787), CDK9 antibody (Cat # ab239364), Myc antibody (Cat # ab32072), Oct4 antibody (Cat # ab181557), SDC2 antibody (Cat # ab308031), ITGA5 antibody (Cat # ab112183), GALK1 antibody (Cat # ab228533), and AXL antibody (Cat # ab219651) were obtained from Abcam. The Fyn Tyr420 antibody was purchased from MyBioSource (Cat # MBS9128730). ACLY antibody (Cat #15421-1-AP), ALDOA antibody (Cat # 11217-1-AP), TWIST1 antibody (Cat # 25465-1-AP), and FGF2 antibody (Cat # 11234-1-AP) were obtained from Proteintech. The MCM3 antibody (Cat # A11475), MCM4 antibody (Cat # A9251), MCM5 antibody (Cat # A5008), MCM6 antibody (Cat # A1955), MCM7 antibody (Cat # A11325), CDC45 antibody (Cat # A16032), CCND1 antibody (Cat # A11022), CCNE1 antibody (Cat # A22360), and MMP14 antibody (Cat # A15691) were obtained from ABclonal. ACLY Tyr542, Tyr652, ALDOA Tyr174, Tyr302, and Tyr328 antibodies were designed and synthesized by ABclonal.

### Cell culture and transfection

To establish metastatic ESCC cells, KYSE410 and KYSE510 cells were injected into the footpads of the mice. After 6 weeks, the metastatic lymph nodes were cut into pieces and cultured in RPMI 1640 medium. An anti-CD326 (Miltenyi Biotec; Cat # 130-061-101) microbead was used to sort ESCC cells for further study.

siRNAs were used to knockdown specific molecules in ESCC cells. Briefly, siRNAs mixed with Lipofectamine 2000 reagent were added to ESCC cells for 48 h, after which the culture medium was replaced. Transfection efficacy was assessed by immunoblotting. The siRNA sequences used were as follows:

ACLY siRNA1: GCTCGATTATGCACTGGAA;

ACLY siRNA2: GACCAAAGATGGAGTCTAT.

ALDOA siRNA1: GTGTCATCCTCTTCCATGA;

ALDOA siRNA2: CCCAAGTTATCAAATCCAA.

For mutant transfection, pcDNA 3.1-Flag plasmids harboring ACLY Y542A, Y652A, or ALDOA Y174A, Y302A, or Y328A, combined with Lipofectamine 2000 reagent, were transfected into ESCC cells. After transfection, positive clones were selected with G418 for subsequent assays.

### MTS assay

The detailed procedure of the MTS assay has been previously described.^88,89^ Briefly, the cells were cultured in 96-well plates and then subjected to the indicated agents for 3 days. MTS solution (Promega; Cat # G3582) was added to evaluate the growth ability of the cells.

### Soft agar colony formation assay

A soft agar-based transformation assay (BioVision; Cat # K921) was applied to assess the anchorage-independent proliferation of cells with or without the indicated agents. The base agarose layer was prepared, and then the top agarose layer containing the cells was cultured. The indicated agents were added and incubated for 8 days. The chromogen was added to each well, and the value of colony growth was spectrophotometrically observed at 450 nm.

### Invasion assay

A Matrigel-based invasion assay (BioVision; Cat # K913) was used to observe the invasiveness of cells in the presence of the indicated agents. Briefly, cells in 200 μL of fetal bovine serum-free medium were plated into a Matrigel-coated upper chamber (8 μm insert). The lower chamber was filled with 800 μL of medium containing 20% fetal bovine serum and then treated with the indicated agents for 24 h. Invaded cells were collected with dissociation solution and then stained with cell dye. The fluorescence value was determined via a microplate reader.

### Aldolase activity assay

Intracellular aldolase activity was evaluated via a relevant assay (Abcam; Cat # ab196994). Briefly, harvested cells were resuspended in 100 μL of cold aldolase assay buffer, vortexed several times, incubated at 4 °C for 20 min, and then centrifuged at 12,000 × *g* for 5 min to collect the supernatants. The supernatants were mixed with aldolase assay buffer (44 μL), substrate (2 μL), aldolase enzyme mixture (2 μL), and aldolase developer (2 μL), and the OD value was subsequently measured at 450 nm.

### G3P assay

The production of G3P from ESCC cells was assessed via a glyceraldehyde 3-phosphate assay kit (Abcam; Cat # ab273344). ESCC cells were dissociated with 100 μL of cold G3P assay buffer for 30 min, and the supernatants (filtered with a 10 kDa spin column during centrifugation) were collected for analysis of the G3P concentration. The samples were incubated with 50 μL of mixture buffer, which included G3P assay buffer (44 μL), G3P developer (2 μL), enzyme mixture (2 μL), and a G3P probe (2 μL). The fluorescence value was read at Ex/Em = 535/587 nm.

### ACLY activity assay

ACLY activity in ESCC cells was evaluated via an ACLY activity assay kit (Cat # BC4245, Solarbio). ESCC cells were added to 200 μL of extraction buffer, including extraction buffers I and II (the ratio of I and II was 99:1 (volume:volume)), sonicated on ice, and then centrifuged at 10,000 × *g* for 10 min to collect the supernatants. The samples (10 μL) were mixed with reaction buffers 1 to 5 (volumes from 1 to 5 were 152, 4, 20, 4, 10, and 10 μL, respectively), and then, the ACLY activity (OD value) was evaluated via spectrophotometry at 450 nm.

### OAA assay

The concentration of OAA in ESCC cells was evaluated via an OAA assay kit (Abcam; Cat # ab83428). ESCC cells were homogenized with 100 μL of OAA assay buffer and then centrifuged at 10,000 × *g* for 10 min to collect supernatants (filtered through a 10 kDa spin column during centrifugation). The samples were mixed with 50 μL of the reaction mixture, which included the assay buffer (44 μL), developer (2 μL), enzyme mixture (2 μL), and OAA probe (2 μL). The OD value of every sample was spectrophotometrically measured at 570 nm.

### Pyruvate assay

The level of pyruvate in ESCC cells was assessed via a pyruvate assay kit (Abcam; Cat # ab65342). Briefly, ESCC cells were harvested and resuspended in pyruvate assay buffer, and then centrifuged to obtain supernatants, which were subjected to a deproteinization step, and samples were collected. The samples were mixed with a total volume of 50 μL, including 46 μL of assay buffer containing samples, 2 μL of probe, and 2 μL of enzyme mixture. The OD value of intracellular pyruvate was detected at 570 nm.

### Luciferase reporter assay

HEK293T cells were seeded onto 24-well plates and cotransfected with c-Myc, Oct4, or Sox2 overexpression plasmids with or without their respective binding sites in the promoter regions of the *AXL*, *GALK1*, *ITGA5*, or *SDC2* genes, including the pGL3-Basic-AXL, GALK1, ITGA5, or SDC2 wild-type or mutant genes, along with the pRL-TK plasmid. After 48 h of transfection, promoter activity was evaluated via a dual-luciferase reporter assay (Cat # E1910, Promega).

### CETSA

ESCC cells were treated with quercetagitrin (50 μM) for 6 hr and then collected and lysed with CETSA lysis buffer 2 (Cat # CETSA-BUF2-100ML, PerkinElmer). The lysates were incubated at a series of temperatures from 37 to 62 °C with a gradient of 5 °C for 3 min and then repeatedly frozen and thawed in liquid nitrogen three times. The supernatants were collected for ELISA to quantitatively evaluate the phosphorylation status of ALDOA.

### Molecular docking

The docking model was constructed via Schrödinger Maestro 11.4 and PyMol software. The human ALDOA protein (PDB ID: 2ALD) was prepared via a protein preparation wizard, and then grid-based ligand docking was applied to screen the MCE bioactive compound library on the basis of ALDOA Tyr174/302/328 surrounding spatial sites. The top scoring compounds were used for further experiments (compound information is listed in Supplementary Table 3).

### ChIP assay

A ChIP assay was used to evaluate the binding ability of CDK7 and CDK9 to DNA in the promoter regions of the *MCM3*, *MCM4*, *MCM5*, *MCM6*, *MCM7*, and *CDC45* genes or Myc, Sox2, and Oct4 to the DNA in the promoter regions of the *AXL*, *GALK1*, *SDC2*, and *ITGA5* genes in ESCC cells. Briefly, the cells were crosslinked with 1% formaldehyde for 10 min at room temperature, and glycine was stopped for 5 min. The fixed cells were resuspended in cell lysis buffer and then in nuclear lysis buffer to obtain a cell suspension, which was subjected to sonication to generate DNA fragments. These obtained fragments were incubated with ChIP buffer and the indicated antibodies overnight at 4 °C on a rotator, and then ChIP-grade protein G magnetic beads were added and incubated for an additional 2 hr. The beads were subsequently washed, and the pulled-down chromatin‒protein complex was reversed cross-linked and incubated for 4 h at 65 °C. The obtained DNA was subjected to RT‒PCR. The primer sequences for the indicated genes are listed in Supplementary Table 6.

### Real-time PCR

RNA was extracted with TRIzol (Invitrogen) and reverse transcribed to cDNA with PrimeScript RT Master Mix (Takara). The obtained cDNA was combined with TB Green Premix Ex Taq (Takara) and subjected to RT‒PCR on an ABI PRISM 7500 detection system (Applied Biosystems). The sequences of primers used are listed in Supplementary Table 7.

### Immunoprecipitation and immunoblotting assays

For immunoprecipitation, cultured cells were washed with cold phosphate-buffered saline (PBS), collected, and centrifuged at 1000 × *g* for 10 min to obtain precipitants, which were subsequently lysed in NP-40 buffer in the presence of protease and phosphatase inhibitors for 1 h at 4 °C. The lysates were centrifuged at 10,000 × *g* for 10 min to collect supernatants, which were subsequently incubated with protein A/G (Thermo Fisher) and the indicated antibodies (1:100) on a rotator at 4 °C overnight. The protein complex was eluted with NP-40 buffer and added to loading buffer for immunoblotting.

For the immunoblotting assay, the collected cells were lysed with RIPA lysis buffer for 1 h at 4 °C. The lysates were centrifuged at 10,000 × *g* for 10 min to obtain supernatants, which were subsequently suspended in loading buffer for electrophoresis. Then, the proteins were transferred to a PVDF membrane, which was sequentially incubated with blocking buffer for 1 h at room temperature, incubated with the indicated antibodies (1:1000, except for GAPDH and β-actin (1:3000)) at 4 °C overnight, washed with PBS-T several times, and incubated with secondary antibodies. Finally, the antigen‒antibody signal was visualized via a chemiluminescent substrate.

### Streptavidin pull-down assay

ESCC cells were lysed with NP-40 buffer containing protease and phosphatase inhibitor cocktail for 60 min. Cell lysates were incubated with biotin-quercetagitrin (50 µM) at 4 °C for 12 hr. Following incubation, streptavidin agarose 6FF was added, and the mixture was incubated overnight at 4 °C. Bead-bound biotin-quercetagitrin lysates were centrifuged and washed with detergent buffer (20 mM NaH_2_PO_4_, 0.15 M NaCl). Loading buffer was mixed with beads and incubated at 100 °C for 5 min. After centrifugation, the supernatant was collected for electrophoresis, and then, biotin-quercetagitrin-interacting ALDOA was detected by immunoblotting.

### In vitro ALDOA activity assay

Two systems were used to evaluate the quercetagitrin-mediated inhibition of recombinant human ALDOA activity in vitro. (1) ALDOA protein (Cat # CSB-EP001583HUa0, CUSABIO) was diluted to a concentration of 250 nM with reaction buffer (50 mM pH 7.4 Tris-HCl, 10 mM EDTA, 20 mM DTT, 0.2 mM NADH, 14 U/mL TPI, and 1 U/mL GPDH) and transferred to a 96-well assay plate (100 µL). Different concentrations of quercetagitrin (0–100 μM) were mixed with the ALDOA protein. The assay plate was incubated at 25 °C for 30 min. After incubation, FBP solution (250 μM) was added to the assay plate to initiate the reaction. The OD value of the NADH level in every well was directly measured at 340 nm. (2) ALDOA protein (250 nM) in reaction buffer containing 50 mM pH 7.5 Tris-HCl, 1 mM EDTA, 0.1% BSA, 1.5 mM DTT, and 0.01% Triton X-100 was incubated with quercetagitrin (0–100 μM) at 25 °C for 30 min. Then, FBP (250 μM), β-NAD^+^ (50 μM), and GAPDH (0.5 U/mL) were added to each well and incubated at 25 °C for 60 min. Finally, NAD/NADH detection solution (Cat # G9071, Promega) was added, and luminescence was read at Ex/Em: 340 nm/360 nm.

### Xenograft mouse model

Female BALB/c-nu mice (4 weeks old) were used in the present study. To evaluate whether quercetagitrin inhibits the lymph node metastasis of ESCC cells by blocking ALDOA activity, KYSE410 and KYSE510 cells were inoculated into the footpads of mice (*n* = 5/group). After ~1 week, AAV8-CMV-ALDOA Y174/302/328 A (1 × 10^11^ vector genomes (vg); twice a week for 4 weeks) was injected into the tumors, and the animals were treated with or without quercetagitrin (25 mg/kg/day, p.o.) for 5 weeks. The synergistic effect of combination treatment on the inhibition of the FAK/SFK axis and ALDOA activity was also assessed in a lymph node metastasis xenograft model. One week after injecting the footpads with ESCC cells, the animals were treated with defactinib (15 mg/kg/day, p.o.) or ponatinib (10 mg/kg/day, p.o.) alone or in combination with quercetagitrin (25 mg/kg/day, p.o.) for 5 weeks. Lymph node volume was evaluated via a previously reported formula,^90^ and representative lymph nodes from every group were subjected to H&E staining. Footpad tumors and lymph nodes were subjected to an IHC assay to evaluate the expression of MCM3, MCM4, MCM5, MCM6, MCM7, and CDC45 in primary tumors and the levels of AXL, GALK1, ITGA5, and SDC2 in the lymph nodes. The dilutions of the primary antibodies used for the animal IHC assay were all 1:100. The expression of CDK7 (EIAAB Science; Cat # E1880h), CDK9 (EIAAB Science; Cat # E8846h), Ki67 (Raybiotech; Cat # ELH-MKI67), and LYVE1 (Raybiotech; Cat # ELH-LYVE1) in primary tumors was measured by corresponding quantitative ELISAs. The transcriptional activity of c-Myc, Sox2, and Oct4 in the lymph nodes was assessed via a human c-Myc activity assay (Raybiotech; Cat # TFEH-CMYC), Sox2 activity assay (Raybiotech; Cat # TFEH-SOX2), and Oct4 activity assay (Raybiotech; Cat # TFEH-Oct4) kits, respectively.

The experimental protocol for the subcutaneous tumor model was described in our previous studies.^88^^–^^91^ After the tumor volume reached ~100 mm^3^, the animals (*n* = 5/group) received quercetagitrin (25 mg/kg/day, p.o.) for 3 weeks. After treatment, the tumor tissues were lysed, and the expression of Ki67 and LYVE-1 in the tumors was subsequently assessed via quantitative ELISA. H&E staining was used to evaluate the morphological changes in the intestine, liver, spleen, kidney, and heart between the control and quercetagitrin treatment groups.

### IHC staining of clinical ESCC tissue samples

The protocol for IHC staining and the calculation of the staining index were performed according to our previous studies.^90,91^ Briefly, Tris-EDTA was applied to retrieve the antigens of ESCC tissues, which were then subjected to blocking solution and incubated with primary antibodies overnight at 4 °C. The dilution of the primary antibodies was 1:100. The tissues were incubated with secondary antibodies for 1 h at room temperature. 3,3 N-diaminobenzidine tetrahydrochloride (DAB) solution was used for visualization. The following formula was used to measure the expression of the indicated proteins: staining intensity × percentage of positive cells.

### Statistical analysis

Analysis was performed via GraphPad Prism software. Unpaired Student’s *t*-test was used to evaluate differences between two groups. The dose-dependent effects in at least three groups were analyzed via one-way ANOVA. Correlations between two parameters were analyzed via two-tailed *χ*^2^ and Pearson tests, as indicated for the clinical samples. *P* values < 0.05, <0.01, or 0.001 were labeled *, **, or ***, respectively.

## Supplementary information

Supplementary Figures, Figure legends, and Tables

## Data Availability

All data in the present article have been deposited in https://figshare.com/, and the accession numbers are: 10.6084/m9.figshare.29906357, 10.6084/m9.figshare.29906363, 10.6084/m9.figshare.29906468 and 10.6084/m9.figshare.29906519.

## References

[1] Niu, C. et al. Risk factors for esophageal squamous cell carcinoma and its histological precursor lesions in China: a multicenter cross-sectional study. *BMC Cancer* **21**, 1034 (2021). 34530751 10.1186/s12885-021-08764-x PMC8444572

[2] Morgan, E. et al. The global landscape of esophageal squamous cell carcinoma and esophageal adenocarcinoma incidence and mortality in 2020 and projections to 2040: new estimates from GLOBOCAN 2020. *Gastroenterology* **163**, 649–658 (2020). 10.1053/j.gastro.2022.05.054 35671803

[3] Kurokawa, Y. et al. Mapping of lymph node metastasis from esophagogastric junction tumors: a prospective nationwide multicenter study. *Ann. Surg.* **274**, 120–127 (2021). 31404008 10.1097/SLA.0000000000003499

[4] Min, B. H. et al. Nomogram for prediction of lymph node metastasis in patients with superficial esophageal squamous cell carcinoma. *J. Gastroenterol. Hepatol.* **35**, 1009–1015 (2020). 31674067 10.1111/jgh.14915

[5] Gockel, I. et al. Risk of lymph node metastasis in submucosal esophageal cancer: a review surgically resected patients. *Expert Rev. Gastroenterol. Hepatol.* **5**, 371–384 (2011). 21651355 10.1586/egh.11.33

[6] Wu, N., Chen, Z., Pang, L., Ma, Q. & Chen, G. Prognostic significance of lymph node characteristics on survival in esophageal squamous cell carcinomas. *Wien. Klin. Wochenschr.* **125**, 26–33 (2013). 23292643 10.1007/s00508-012-0310-2

[7] Wang, H., Deng, F., Liu, Q. & Ma, Y. Prognostic significance of lymph node metastasis in esophageal squamous cell carcinoma. *Pathol. Res. Pract.* **213**, 842–847 (2017). 28554754 10.1016/j.prp.2017.01.023

[8] Hsu, P. K., Lee, Y. Y., Chuang, L. C. & Wu, Y. C. Lymph node dissection for esophageal squamous cell carcinoma. *Thorac. Surg. Clin.* **32**, 497–510 (2022). 36266036 10.1016/j.thorsurg.2022.07.001

[9] Hebert, J. D., Neal, J. W. & Winslow, N. M. Dissecting metastasis using preclinical models and methods. *Nat. Rev. Cancer* **23**, 391–407 (2023). 37138029 10.1038/s41568-023-00568-4

[10] Steeg, P. S. Targeting metastasis. *Nat. Rev. Cancer* **16**, 201–218 (2016). 27009393 10.1038/nrc.2016.25 PMC7055530

[11] Stacker, S. A. et al. Lymphangiogenesis and lymphatic vessel remodeling in cancer. *Nat. Rev. Cancer* **14**, 159–172 (2014). 24561443 10.1038/nrc3677

[12] Valastyan, S. & Weinberg, R. A. Tumor metastasis: molecular insights and evolving paradigms. *Cell* **147**, 275–292 (2011). 22000009 10.1016/j.cell.2011.09.024 PMC3261217

[13] Kim, M. et al. CXCR4 signaling regulates metastasis of chemoresistant melanoma cells by a lymphatic metastatic niche. *Cancer Res.* **70**, 10411–10421 (2010). 21056990 10.1158/0008-5472.CAN-10-2591

[14] Jeong, H. S. et al. Investigation of the lack of angiogenesis in the formation of lymph node metastases. *J. Natl. Cancer Inst.* **107**, djv155 (2015). 26063793 10.1093/jnci/djv155 PMC4651102

[15] Dong, H. et al. YB-1 expression is associated with lymph node metastasis and drug resistance to Adriamycin in breast cancer. *Dis. Markers* **2023**, 4667089 (2023). 36785738 10.1155/2023/4667089 PMC9922184

[16] Zhang, C. et al. Noninvasive imaging of CD206-positive M2 macrophages as an early biomarker for post-chemotherapy tumor relapse and lymph node metastasis. *Theranostics* **7**, 4276–4288 (2017). 29158825 10.7150/thno.20999 PMC5695012

[17] Zheng, H. et al. PDGFRα^+^ITGA11^+^ fibroblasts foster early-stage cancer lymphovascular invasion and lymphatic metastasis via ITGA11-SELE interplay. *Cancer Cell* **42**, 682–700 (2024). 38428409 10.1016/j.ccell.2024.02.002

[18] Huang, Q. et al. Multi-omics analysis reveals NNMT as a master metabolic regulator of metastasis in esophageal squamous cell carcinoma. *NPJ Precis Oncol.* **8**, 24 (2024). 38291241 10.1038/s41698-024-00509-w PMC10828394

[19] Huang, H. et al. Multi-omics analyses reveal spatial heterogeneity in primary and metastatic oesophageal squamous cell carcinoma. *Clin. Transl. Med.* **13**, e1493 (2023). 38009315 10.1002/ctm2.1493 PMC10679972

[20] Peng, W., Qiao, H., Mo, L. & Guo, Y. Progress in the diagnosis of lymph node metastasis in rectal cancer: a review. *Front. Oncol.* **13**, 1167289 (2023). 37519802 10.3389/fonc.2023.1167289 PMC10374255

[21] Sharma, K. et al. Ultradeep human phosphoproteome reveals a distinct regulatory nature of Tyr and Ser/Thr-based signaling. *Cell Rep.* **8**, 1583–1594 (2014). 25159151 10.1016/j.celrep.2014.07.036

[22] Sefton, B. M., Hunter, T., Beemon, K. & Eckhart, W. Evidence that the phosphorylation of tyrosine is essential for cellular transformation by Rous sarcoma virus. *Cell* **20**, 807–816 (1980). 6251974 10.1016/0092-8674(80)90327-x

[23] Kolch, W. & Pitt, A. Functional proteomics to dissect tyrosine kinase signaling pathways in cancer. *Nat. Rev. Cancer* **10**, 618–629 (2010). 20720570 10.1038/nrc2900

[24] Lundby, A. et al. Oncogenic mutations rewire signaling pathways by switching protein recruitment to phosphotyrosine sites. *Cell* **179**, 543–560 (2019). 31585087 10.1016/j.cell.2019.09.008 PMC7618132

[25] Zhang, Q. et al. ALK phosphorylates SMAD4 on tyrosine to disable TGF-beta tumour suppressor functions. *Nat. Cell Biol.* **21**, 179–189 (2019). 30664791 10.1038/s41556-018-0264-3

[26] Rikova, K. et al. Global survey of phosphotyrosine signaling identifies oncogenic kinases in lung cancer. *Cell* **131**, 1190–1203 (2007). 18083107 10.1016/j.cell.2007.11.025

[27] Cerulli, R. A. & Kritzer, J. A. Phosphotyrosine isosteres: past, present and future. *Org. Biomol. Chem.* **18**, 583–605 (2020). 31777907 10.1039/c9ob01998g PMC7233463

[28] Cooper, A. J., Sequist, L. V. & Lin, J. J. Third-generation EGFR and ALK inhibitors: mechanisms of resistance and management. *Nat. Rev. Clin. Oncol.* **19**, 499–514 (2022). 35534623 10.1038/s41571-022-00639-9 PMC9621058

[29] Zhang, J., Yang, P. & Gray, N. Targeting cancer with small molecule kinase inhibitors. *Nat. Rev. Cancer* **9**, 28–39 (2009). 19104514 10.1038/nrc2559 PMC12406740

[30] Dawson, J. C., Serrels, A., Stupack, D. G., Schlaepfer, D. D. & Frame, M. C. Targeting FAK in anticancer combination therapies. *Nat. Rev. Cancer* **21**, 313–324 (2021). 33731845 10.1038/s41568-021-00340-6 PMC8276817

[31] Sulzmaier, F. J., Jean, C. & Schlaepfer, D. D. FAK in cancer: mechanistic findings and clinical applications. *Nat. Rev. Cancer* **14**, 598–610 (2014). 25098269 10.1038/nrc3792 PMC4365862

[32] Mitra, S. K. & Schlaepfer, D. D. Integrin-regulated FAK/Src signaling in normal and cancer cells. *Curr. Opin. Cell Biol.* **18**, 516–523 (2006). 16919435 10.1016/j.ceb.2006.08.011

[33] Kim, L. C., Song, L. & Haura, E. B. Src kinases as therapeutic targets for cancer. *Nat. Rev. Clin. Oncol.* **6**, 587–595 (2009). 19787002 10.1038/nrclinonc.2009.129

[34] Lee, B. Y., Timpson, P., Horvath, L. G. & Daly, R. J. FAK signaling in human cancer as a target for therapeutics. *Pharm. Ther.* **146**, 132–149 (2015). 10.1016/j.pharmthera.2014.10.001 25316657

[35] Arang, N. et al. High-throughput chemogenetic drug screening reveals PKC-RhoA/PKN as a targetable signaling vulnerability in GNAQ-driven uveal melanoma. *Cell Rep. Med.* **4**, 101244 (2023). 37858338 10.1016/j.xcrm.2023.101244 PMC10694608

[36] Chen, J. et al. Tumor-associated macrophage (TAM)-derived CCL22 induces FAK addiction in esophageal squamous cell carcinoma (ESCC). *Cell Mol. Immunol.* **19**, 1054–1066 (2022). 35962191 10.1038/s41423-022-00903-z PMC9424285

[37] Zhang, J. et al. Src heterodimerically activates Lyn or Fyn to serve as targets for the diagnosis and treatment of esophageal squamous cell carcinoma. *Sci. China Life Sci.* **66**, 1245–1263 (2023). 36763244 10.1007/s11427-022-2216-x

[38] Martinez-Outschoorn, U. E., Peiris-Pagés, M., Pestell, R. G., Sotgia, F. & Lisanti, M. P. Cancer metabolism: a therapeutic perspective. *Nat. Rev. Clin. Oncol.* **14**, 11–31 (2017). 27141887 10.1038/nrclinonc.2016.60

[39] Mohanty, V. Inferring cancer metabolism from gene-expression data. *Nat. Rev. Cancer* **24**, 230 (2024). 38316946 10.1038/s41568-024-00670-1

[40] Granchi, C. ATP citrate lyase (ACLY) inhibitors: an anti-cancer strategy at the crossroads of glucose and lipid metabolism. *Eur. J. Med. Chem.* **157**, 1276–1291 (2018). 30195238 10.1016/j.ejmech.2018.09.001

[41] Zhou, J. et al. Aldolase A promotes cervical cancer cell radioresistance by regulating the glycolysis and DNA damage after irradiation. *Cancer Biol. Ther.* **24**, 2287128 (2023). 38010897 10.1080/15384047.2023.2287128 PMC10761068

[42] Guo, D. et al. Aerobic glycolysis promotes tumor immune evasion by hexokinase 2-mediated phosphorylation of IκBα. *Cell Metab.* **34**, 1312–1324 (2022). 36007522 10.1016/j.cmet.2022.08.002

[43] Chen, T. et al. AKT1 phosphorylation of cytoplasmic ME2 induces a metabolic switch to glycolysis for tumorigenesis. *Nat. Commun.* **15**, 686 (2024). 38263319 10.1038/s41467-024-44772-8 PMC10805786

[44] Schneider, J. L. et al. GUK1 activation is a metabolic liability in lung cancer. *Cell* **188**, 1248–1264 (2025). 39919745 10.1016/j.cell.2025.01.024 PMC12148050

[45] Liu, Z. et al. Integrated multi-omics profiling yields a clinically relevant molecular classification for esophageal squamous cell carcinoma. *Cancer Cell* **41**, 181–195 (2023). 36584672 10.1016/j.ccell.2022.12.004

[46] Cui, Y. et al. Whole-genome sequencing of 508 patients identifies key molecular features associated with poor prognosis in esophageal squamous cell carcinoma. *Cell Res.* **30**, 902–913 (2020). 32398863 10.1038/s41422-020-0333-6 PMC7608103

[47] Li, N., Gao, N. & Zhai, Y. DDK promotes DNA replication initiation: mechanistic and structural insights. *Curr. Opin. Struct. Biol.* **78**, 102504 (2023). 36525878 10.1016/j.sbi.2022.102504

[48] Zhao, D. et al. PAFR/Stat3 axis maintains the symbiotic ecosystem between tumor and stroma to facilitate tumor malignancy. *Acta Pharm. Sin. B* **13**, 694–708 (2023). 36873192 10.1016/j.apsb.2022.08.014 PMC9978919

[49] Hu, H. et al. AnimalTFDB 3.0: a comprehensive resource for annotation and prediction of animal transcription factors. *Nucleic Acids Res.* **47**, D33–D38 (2019). 30204897 10.1093/nar/gky822 PMC6323978

[50] Robinson, D. R. et al. Integrative clinical genomics of metastatic cancer. *Nature* **548**, 297–303 (2017). 28783718 10.1038/nature23306 PMC5995337

[51] Martínez-Reyes, I. & Chandel, N. S. Cancer metabolism: looking forward. *Nat. Rev. Cancer* **21**, 669–680 (2021). 34272515 10.1038/s41568-021-00378-6

[52] Pavlova, N. N., Zhu, J. & Thompson, C. B. The hallmarks of cancer metabolism: still emerging. *Cell Metab.* **34**, 355–377 (2022). 35123658 10.1016/j.cmet.2022.01.007 PMC8891094

[53] Zhang, Y. et al. Macrophage-associated PGK1 phosphorylation promotes aerobic glycolysis and tumorigenesis. *Mol. Cell* **71**, 201–215 (2018). 30029001 10.1016/j.molcel.2018.06.023

[54] Wu, K. et al. Creatine kinase B suppresses ferroptosis by phosphorylating GPX4 through a moonlighting function. *Nat. Cell Biol.* **25**, 714–725 (2023). 37156912 10.1038/s41556-023-01133-9

[55] Feng, X. et al. A platform of synthetic lethal gene interaction networks reveals that the GNAQ uveal melanoma oncogene controls the hippo pathway through FAK. *Cancer Cell* **35**, 457–472 (2019). 30773340 10.1016/j.ccell.2019.01.009 PMC6737937

[56] Ma, B., Wells, A. & Clark, A. M. The pan-therapeutic resistance of disseminated tumor cells: role of phenotypic plasticity and the metastatic microenvironment. *Semin. Cancer Biol.* **60**, 138–147 (2020). 31376430 10.1016/j.semcancer.2019.07.021 PMC6992520

[57] Anderson, R. L. et al. A framework for the development of effective anti-metastatic agents. *Nat. Rev. Clin. Oncol.* **16**, 185–204 (2019). 30514977 10.1038/s41571-018-0134-8 PMC7136167

[58] Esposito, M., Ganesan, S. & Kang, Y. Emerging strategies for treating metastasis. *Nat. Cancer* **2**, 258–270 (2021). 33899000 10.1038/s43018-021-00181-0 PMC8064405

[59] Villanueva, M. T. Targeting inherited mutations to prevent metastasis. *Nat. Rev. Drug Discov.* **24**, 90 (2025). 39806011 10.1038/d41573-025-00009-x

[60] Cao, T. et al. Cancer SLC6A6-mediated taurine uptake transactivates immune checkpoint genes and induces exhaustion in CD8^+^ T cells. *Cell* **187**, 2288–2304 (2024). 38565142 10.1016/j.cell.2024.03.011

[61] Lin, H. et al. Itaconate transporter SLC13A3 impairs tumor immunity via endowing ferroptosis resistance. *Cancer Cell* **42**, 2032–2044 (2024). 39515327 10.1016/j.ccell.2024.10.010 PMC11631639

[62] Teng, H. et al. Gut microbiota-mediated nucleotide synthesis attenuates the response to neoadjuvant chemoradiotherapy in rectal cancer. *Cancer Cell* **41**, 124–138 (2023). 36563680 10.1016/j.ccell.2022.11.013

[63] He, Y. et al. Nuclear localization of metabolic enzymes in immunity and metastasis. *Biochim Biophys. Acta Rev. Cancer* **1868**, 359–371 (2017). 28757126 10.1016/j.bbcan.2017.07.002

[64] Yang, W. et al. ERK1/2-dependent phosphorylation and nuclear translocation of PKM2 promotes the Warburg effect. *Nat. Cell Biol.* **14**, 1295–1304 (2012). 23178880 10.1038/ncb2629 PMC3511602

[65] Lu, X. et al. EGFR signaling promotes nuclear translocation of plasma membrane protein TSPAN8 to enhance tumor progression via STAT3-mediated transcription. *Cell Res.* **32**, 359–374 (2022). 35197608 10.1038/s41422-022-00628-8 PMC8975831

[66] Lee, J. et al. EGFR-phosphorylated platelet isoform of phosphofructokinase 1 promotes PI3K activation. *Mol. Cell* **70**, 197–210 (2018). 29677490 10.1016/j.molcel.2018.03.018 PMC6114939

[67] Liu, R. et al. Tyrosine phosphorylation activates 6-phosphogluconate dehydrogenase and promotes tumor growth and radiation resistance. *Nat. Commun.* **10**, 991 (2019). 30824700 10.1038/s41467-019-08921-8 PMC6397164

[68] Song, Y. et al. Identification of genomic alterations in oesophageal squamous cell cancer. *Nature* **509**, 91–95 (2014). 24670651 10.1038/nature13176

[69] Zhang, L. et al. Genomic analyses reveal mutational signatures and frequently altered genes in esophageal squamous cell carcinoma. *Am. J. Hum. Genet.* **96**, 597–611 (2015). 25839328 10.1016/j.ajhg.2015.02.017 PMC4385186

[70] Qin, H. et al. Genomic characterization of esophageal squamous cell carcinoma reveals critical genes underlying tumorigenesis and poor prognosis. *Am. J. Hum. Genet.* **98**, 709–727 (2016). 27058444 10.1016/j.ajhg.2016.02.021 PMC4833434

[71] Ma, F. et al. Heterogeneity analysis of esophageal squamous cell carcinoma in cell lines, tumor tissues and patient-derived xenografts. *J. Cancer* **12**, 3930–3944 (2021). 34093800 10.7150/jca.52286 PMC8176252

[72] Peuget, S., Zhou, X. & Selivanova, G. Translating p53-based therapies for cancer into the clinic. *Nat. Rev. Cancer* **24**, 192–215 (2024). 38287107 10.1038/s41568-023-00658-3

[73] Wang, H., Guo, M., Wei, H. & Chen, Y. Targeting p53 pathways: mechanisms, structures, and advances in therapy. *Signal Transduct. Target Ther.* **8**, 92 (2023). 36859359 10.1038/s41392-023-01347-1 PMC9977964

[74] Vousden, K. H. & Ryan, K. M. p53 and metabolism. *Nat. Rev. Cancer* **9**, 691–700 (2009). 19759539 10.1038/nrc2715

[75] Hassin, O. & Oren, M. Drugging p53 in cancer: one protein, many targets. *Nat. Rev. Drug Discov.* **22**, 127–144 (2023). 36216888 10.1038/s41573-022-00571-8 PMC9549847

[76] Kaiser, A. M. & Attardi, L. D. Deconstructing networks of p53-mediated tumor suppression in vivo. *Cell Death Differ.* **25**, 93–103 (2018). 29099489 10.1038/cdd.2017.171 PMC5729531

[77] Stratton, M. R., Campbell, P. J. & Futreal, P. A. The cancer genome. *Nature* **458**, 719–724 (2009). 19360079 10.1038/nature07943 PMC2821689

[78] Martínez-Jiménez, F. et al. A compendium of mutational cancer driver genes. *Nat. Rev. Cancer* **20**, 555–572 (2020). 32778778 10.1038/s41568-020-0290-x

[79] Chou, J., Quigley, D. A., Robinson, T. M., Feng, F. & Ashworth, A. Transcription-associated cyclin-dependent kinases as targets and biomarkers for cancer therapy. *Cancer Discov.* **10**, 351–370 (2020). 32071145 10.1158/2159-8290.CD-19-0528

[80] Minzel, W. et al. Small molecules co-targeting CKIα and the transcriptional kinases CDK7/9 control AML in preclinical models. *Cell* **175**, 171–185 (2018). 30146162 10.1016/j.cell.2018.07.045 PMC6701634

[81] Lv, M. et al. CDK7-YAP-LDHD axis promotes D-lactate elimination and ferroptosis defense to support cancer stem cell-like properties. *Signal Transduct. Target Ther.* **8**, 302 (2023). 37582812 10.1038/s41392-023-01555-9 PMC10427695

[82] Graham, D. K., DeRyckere, D., Davies, K. D. & Earp, H. S. The TAM family: phosphatidylserine-sensing receptor tyrosine kinase gone awry in cancer. *Nat. Rev. Cancer* **14**, 769–785 (2014). 25568918 10.1038/nrc3847

[83] Myers, K. V., Amend, S. R. & Pienta, K. J. Targeting Tyro3, Axl and MerTK (TAM receptors): implications for macrophages in the tumor microenvironment. *Mol. Cancer* **18**, 94 (2019). 31088471 10.1186/s12943-019-1022-2 PMC6515593

[84] Katoh, M. FGFR inhibitors: effects on cancer cells, tumor microenvironment and whole-body homeostasis (Review). *Int. J. Mol. Med.* **38**, 3–15 (2016). 27245147 10.3892/ijmm.2016.2620 PMC4899036

[85] Sharma, A. et al. Onco-fetal reprogramming of endothelial cells drives immunosuppressive macrophages in hepatocellular carcinoma. *Cell* **183**, 377–394 (2020). 32976798 10.1016/j.cell.2020.08.040

[86] Hood, J. L. Melanoma exosomes enable tumor tolerance in lymph nodes. *Med. Hypotheses* **90**, 11–13 (2016). 27063077 10.1016/j.mehy.2016.02.018 PMC4829918

[87] Alderton, G. K. Metastasis: active lymph nodes. *Nat. Rev. Cancer* **13**, 606–607 (2013). 23969687 10.1038/nrc3587

[88] Chen, J. et al. Chrysin serves as a novel inhibitor of DGKα/FAK interaction to suppress the malignancy of esophageal squamous cell carcinoma (ESCC). *Acta Pharm. Sin. B* **11**, 143–155 (2021). 33532186 10.1016/j.apsb.2020.07.011 PMC7838054

[89] Zhang, L. et al. Focal adhesion kinase (FAK) inhibitor-defactinib suppresses the malignant progression of human esophageal squamous cell carcinoma (ESCC) cells via effective blockade of PI3K/AKT axis and downstream molecular network. *Mol. Carcinog.* **60**, 113–124 (2021). 33283357 10.1002/mc.23273

[90] Chen, J. et al. AKT2^S128^/CCTα^S315/319/323^-positive cancer-associated fibroblasts (CAFs) mediate focal adhesion kinase (FAK) inhibitors resistance via secreting phosphatidylcholines (PCs). *Signal Transduct. Target Ther.* **9**, 21 (2024). 38280862 10.1038/s41392-023-01728-6 PMC10821909

[91] Chen, J. et al. Tumor-associated macrophage (TAM)-secreted CCL22 confers cisplatin resistance of esophageal squamous cell carcinoma (ESCC) cells via regulating the activity of diacylglycerol kinase α (DGKα)/NOX4 axis. *Drug Resist. Update* **73**, 101055 (2024). 10.1016/j.drup.2024.101055 38387281

