# Supplementary material for: Focal adhesion kinase/Src family kinase axis-mediated tyrosine phosphorylation of metabolic enzymes facilitates tumor metastasis
Source: Signal Transduct Target Ther. 2025 Sep 1;10:280. doi: 10.1038/s41392-025-02395-5 (PMC12399764; doi:10.1038/s41392-025-02395-5)
Supplement: Supplementary file 1 — Supplementary Figures, Figure legends, and Tables [file 41392_2025_2395_MOESM1_ESM.docx]

Supplementary Materials for

**Focal adhesion kinase/Src family kinase axis-mediated tyrosine phosphorylation of metabolic enzymes facilitates tumor metastasis**

Jie Chen^1,2,3,4#^*, Jing Zhang ^1,2,3#^, Yuheng Zhu^1,2,3^, Yanmeng Zhu^1,2,3^, Jingyuan Pang ^1^, Qingnan Wu^1,2,3^, Yan Wang^1,2,3^ and Qimin Zhan^1,2,3,4,5^*

Correspondence to: [zhanqimin@bjmu.edu.cn](mailto:zhanqimin@bjmu.edu.cn), cj_blue@126.com

**This file includes:**

Figure. S1 to S24

Table. S1 to S7

**Supplementary Figure legends and Figures**


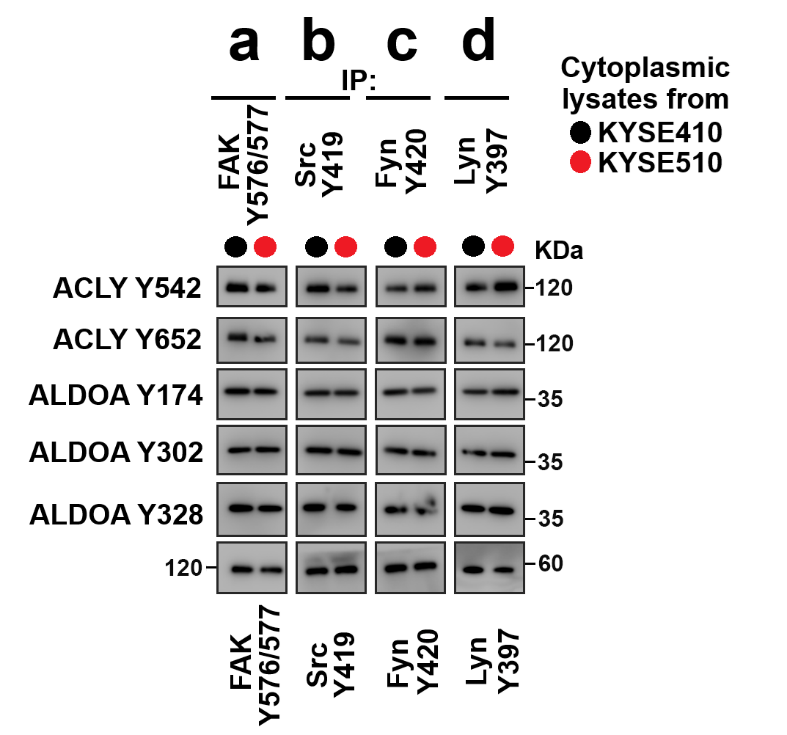


**Supplementary Fig. 1 FAK/SFK axis phosphorylates specific tyrosine sites of ACLY and ALDOA in the cytoplasm of ESCC cells**

**a-d** Cytoplasmic protein extracts of KYSE410 and KYSE510 cells were collected, and then immunoprecipitated with pFAK Tyr576/577 **(a)**, pSrc Tyr419 **(b)**, pFyn Tyr420 **(c)**, or pLyn Tyr397 **(d)**, respectively. Subsequently, immunocomplexes were immunoblotted using antibodies against FAK/SFK axis, and pACLY Tyr542, Tyr652, and pALDOA Tyr174, Tyr302, or Tyr328.


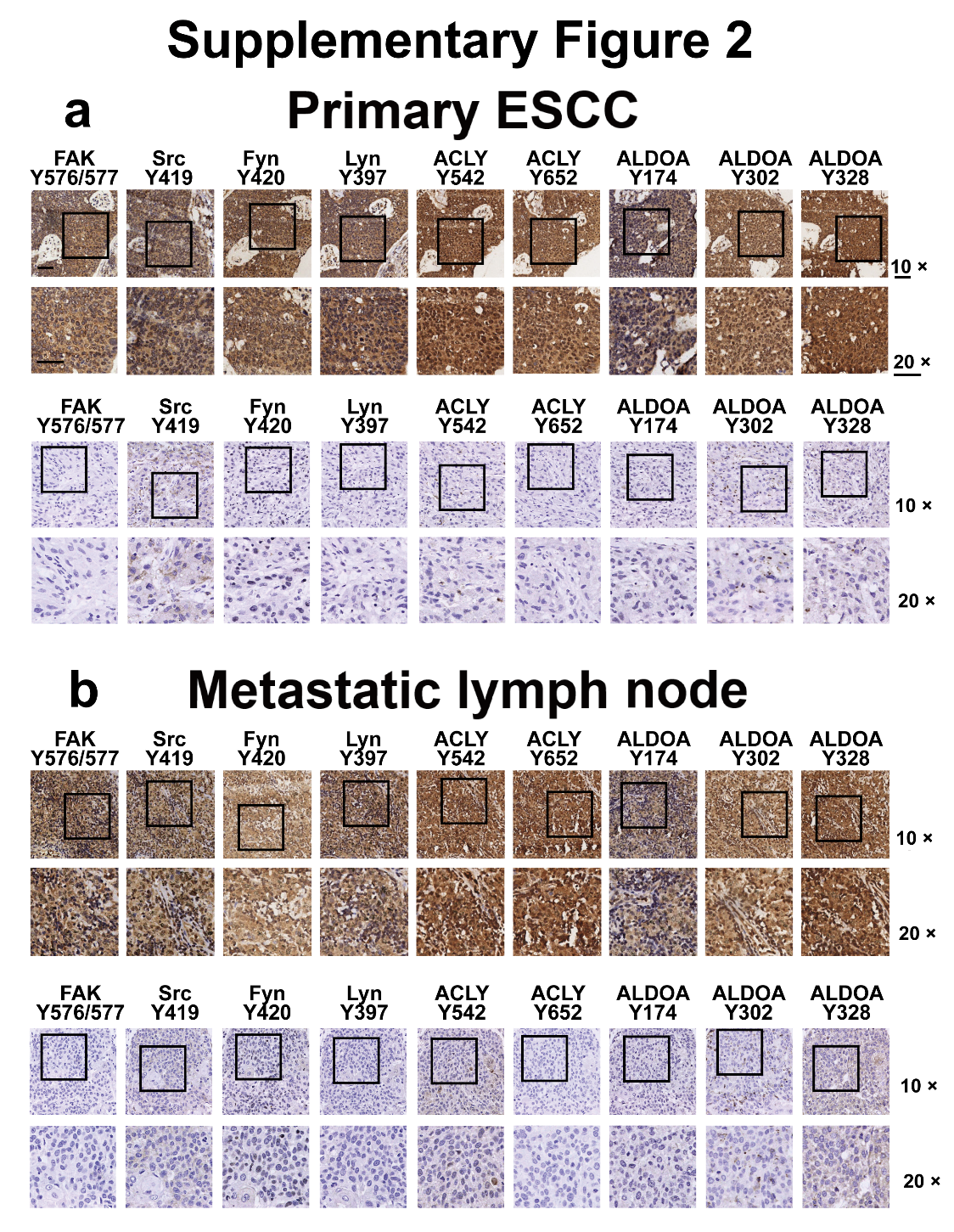


**Supplementary Fig. 2 The clinical correlation between FAK/SFK axis and ACLY Tyr542, Tyr652, or ALDOA Tyr174, Tyr302, Tyr328 in both primary and metastatic ESCC tissues**

**a** Immunohistochemical staining showing the positive correlation between the expression of pFAK Tyr576/577, pSrc Tyr419, pFyn Tyr420, or pLyn Tyr397 with that of pACLY Tyr542, Tyr652, or pALDOA Tyr174, Tyr302, Tyr328 in sequential slices of primary ESCC tissues (approximately *n* = 18). **b** Immunohistochemical staining showing the positive correlation between the expression of pFAK Tyr576/577, pSrc Tyr419, pFyn Tyr420, or pLyn Tyr397 with that of pACLY Tyr542, Tyr652, or pALDOA Tyr174, Tyr302, Tyr328 in sequential slices of metastatic lymph nodes (approximately *n* = 20). Magnification, 10 ×, 20 × as indicated. The statistical information was listed in Supplementary Table 1 and 2.


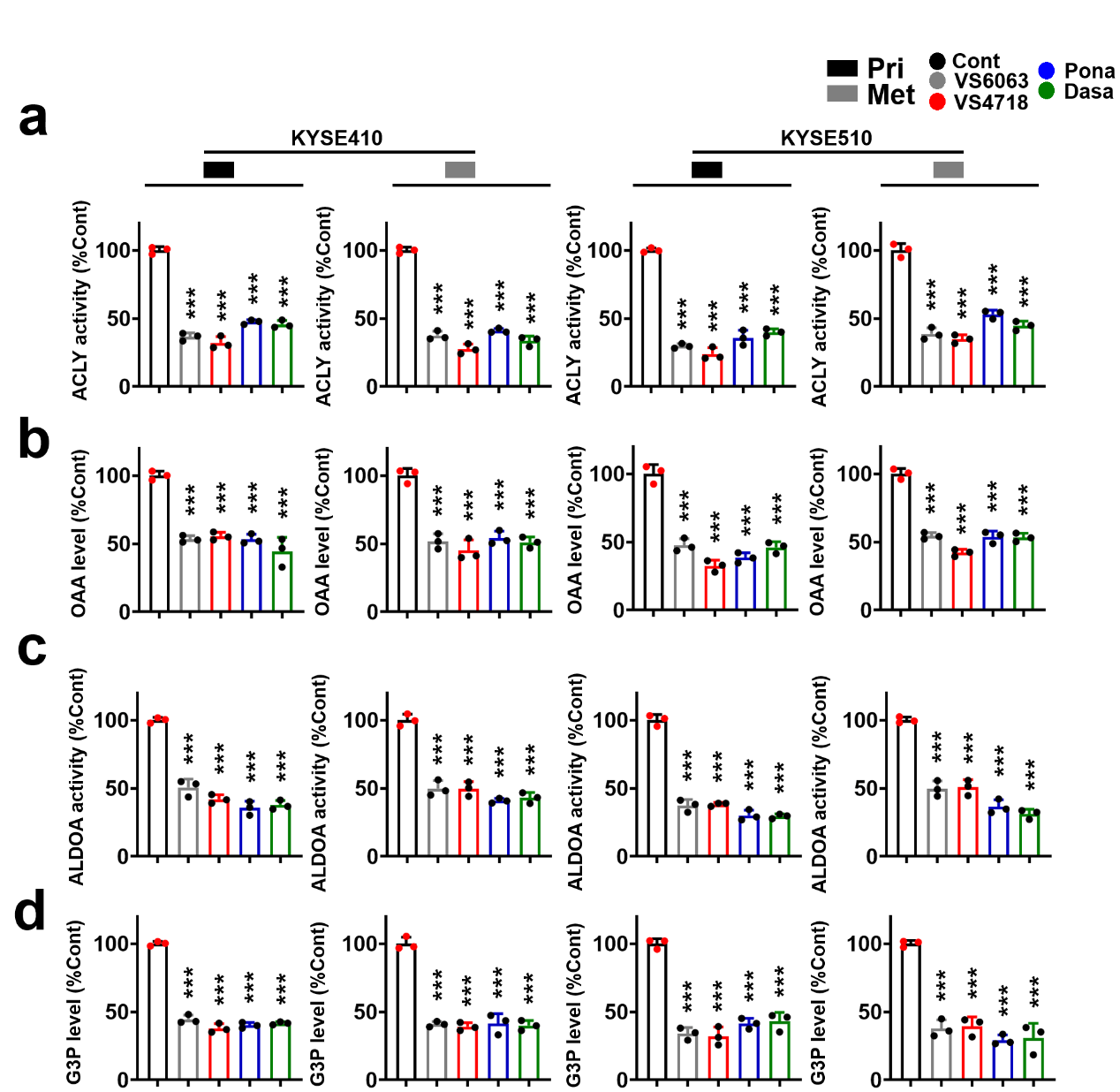


**Supplementary Fig. 3 FAK/SFK inhibitors block the activity of ACLY and ALDOA and the production of OAA and G3P in both primary and metastatic ESCC cells**

**a-d** Primary (black box) and metastatic (gray box) KYSE410 and KYSE510 cells were treated with VS-6063 or VS-4718 (2.5 μM), and ponatinib (5 μM), or dasatinib (0.5 μM), the activity of ACLY **(a)** or the production of OAA **(b)**, and the activity of ALDOA **(c)** or G3P level **(d)** were respectively evaluated (*n* = 3). The data are presented as the means ± SDs. ****P* < 0.001.


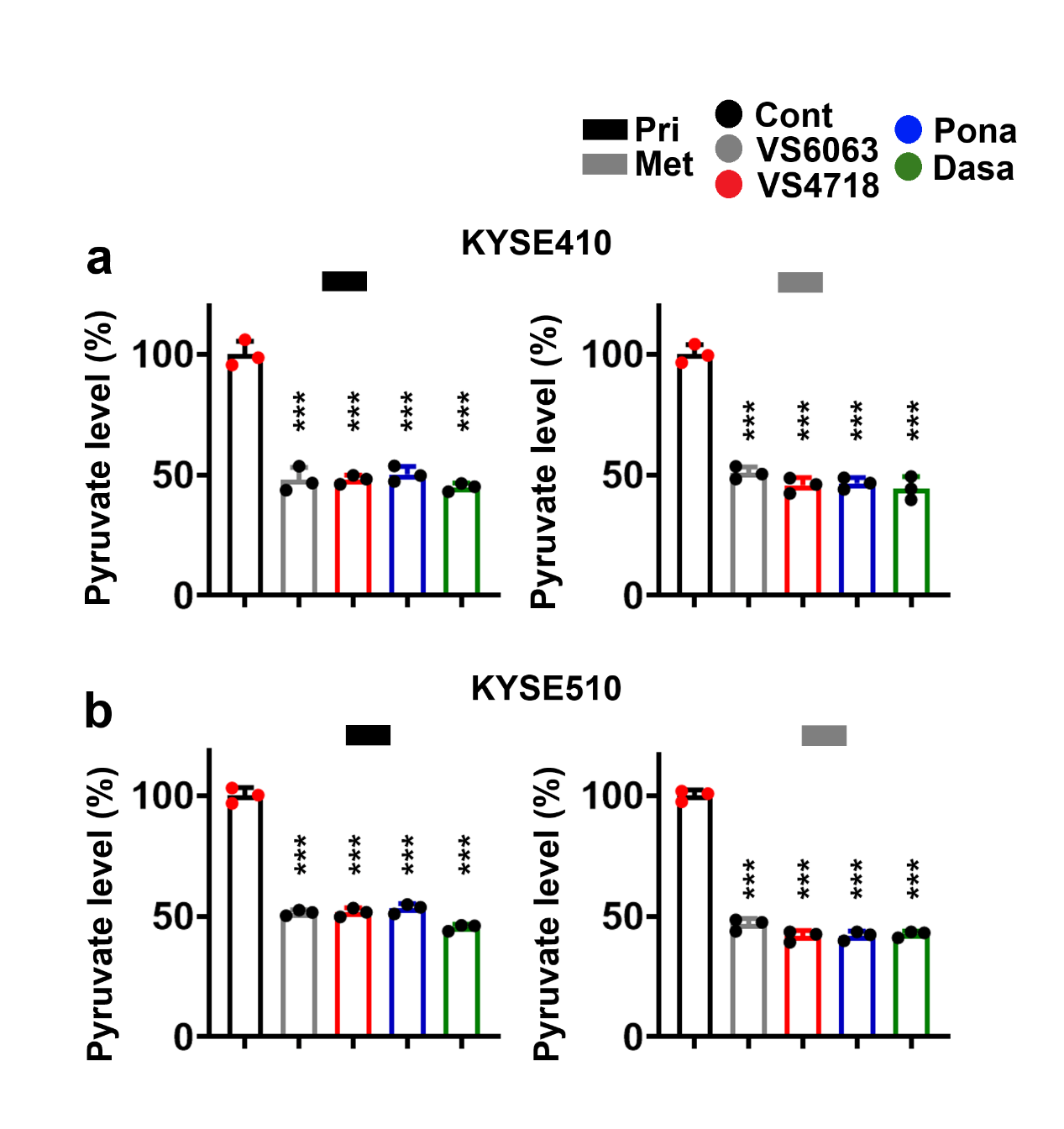


**Supplementary Fig. 4 FAK/SFK inhibitors suppress the production of pyruvate in both primary and metastatic ESCC cells**

**a-b** Primary (black box) and metastatic (gray box) KYSE410 **(a)** and KYSE510 **(b)** cells were treated with VS-6063 or VS-4718 (2.5 μM), and ponatinib (5 μM), or dasatinib (0.5 μM), the pyruvate level was evaluated (*n* = 3). The data are presented as the means ± SDs. ****P* < 0.001.


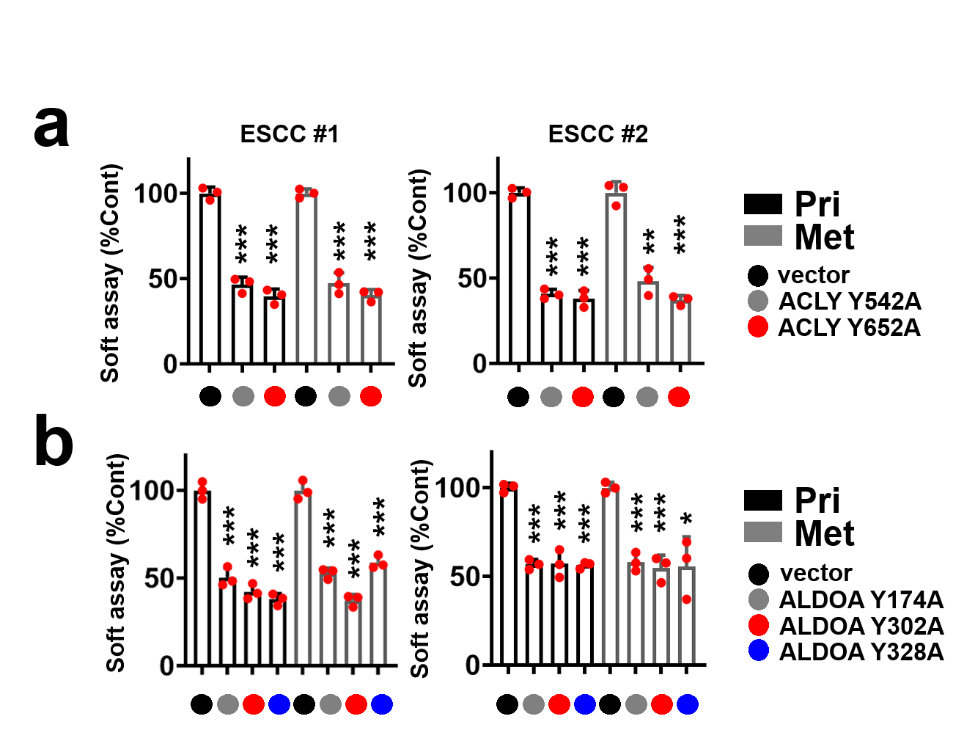


**Supplementary Fig. 5 Mutation of ACLY and ALDOA specific tyrosine sites inhibits the anchorage-independent growth of primary and metastatic ESCC cells**

**a-b** The anchorage-independent growth of primary (black box) and metastatic (gray box) ESCC cells harboring ACLY Y542A, Y652A **(a)** and ALDOA Y174A, Y302A, or Y328A **(b)** were assessed using soft agar assay (*n* = 3). The data are presented as the means ± SDs. **P* < 0.05; ***P* <0.01; ****P* < 0.001.


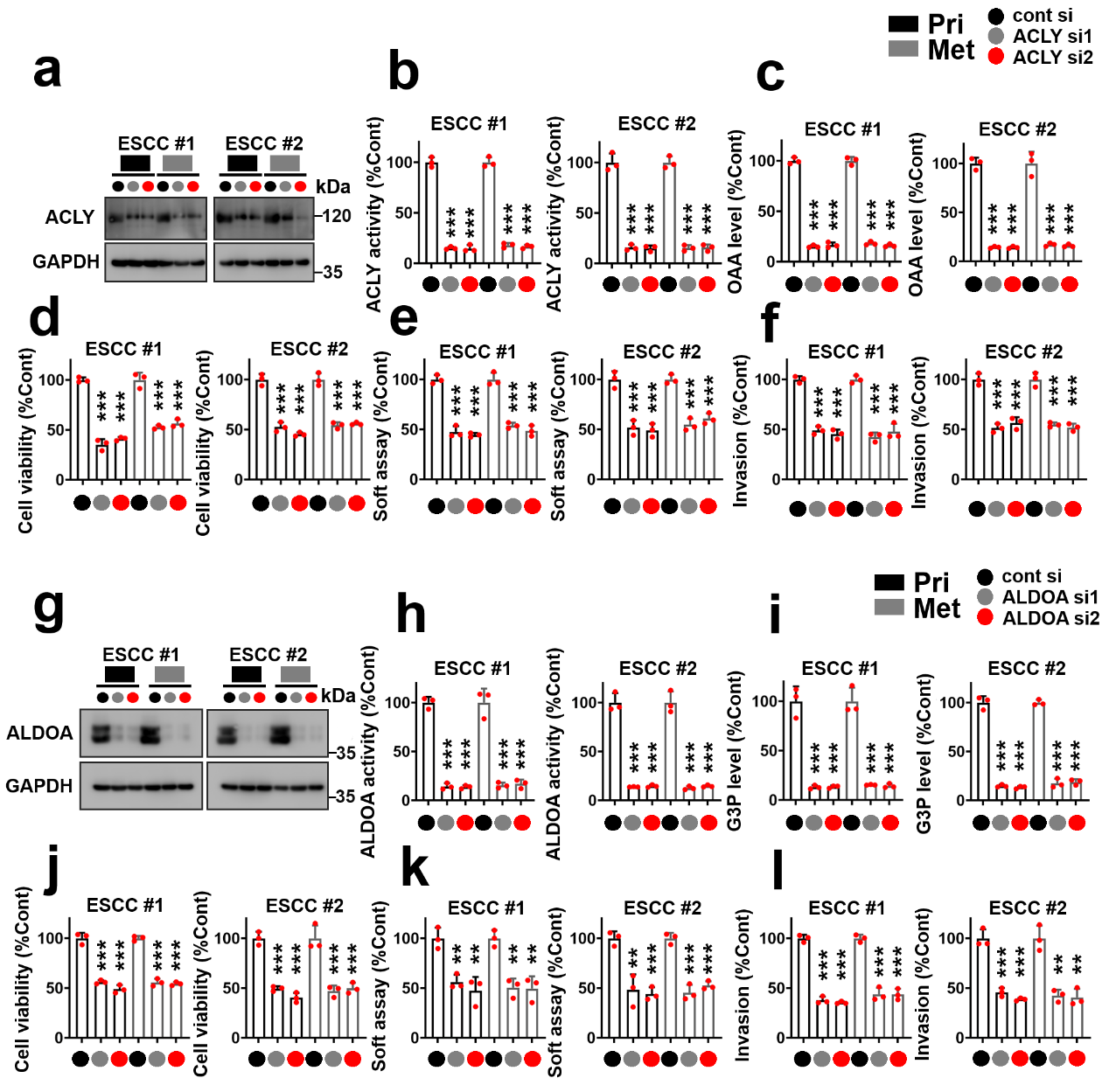


**Supplementary Fig. 6 ACLY and ALDOA siRNAs inhibit the malignant progression of primary and metastatic ESCC cells**

**a** Primary and metastatic ESCC cells were transfected with ACLY siRNA1 and 2, and transfection efficacy was assessed using immunoblotting. **b-c** The ACLY activity **(b)** and the production of OAA **(c)** in ESCC cells harboring ACLY siRNA1 and 2 were also measured (*n* = 3). **d-f** Growth and invasion of ESCC cells were evaluated using MTS **(d)**, soft agar **(e)**, or matrigel-based transwell assay (*n* = 3) **(f)**. **g** Primary and metastatic ESCC cells were transfected with ALDOA siRNA1 and 2, and transfection efficacy was assessed using immunoblotting. **h-i** The ALDOA activity **(h)** and the production of G3P **(i)** in ESCC cells harboring ALDOA siRNA1 and 2 were also measured. **j-l** Growth and invasion of ESCC cells were evaluated using MTS **(j)**, soft agar **(k)**, or matrigel-based transwell assay (*n* = 3) **(l)**. The data are presented as the means ± SDs. ***P* <0.01; ****P* < 0.001.


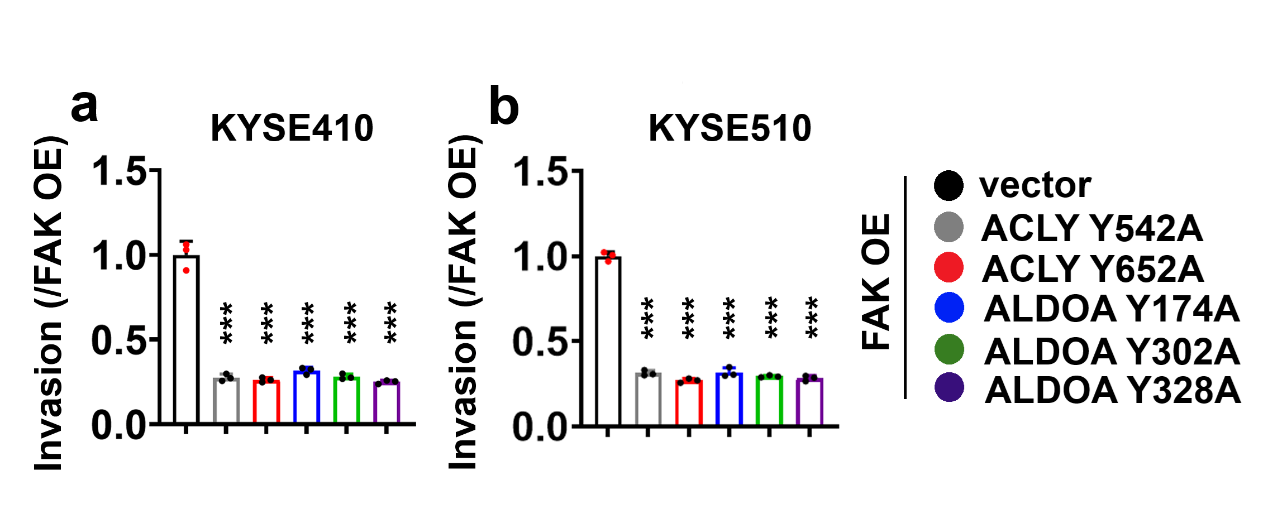


**Supplementary Fig. 7 FAK/ALDOA and ACLY axes mediate the invasion of ESCC cells**

**a-b** KYSE410 **(a)** and KYSE510 **(b)** harboring FAK-overexpressing plasmid (FAK OE) was transfected with ACLY Y542A, Y652A, or ALDOA Y174A, Y302A, Y328A, and the invasive ability of KYSE410 **(a)** and KYSE510 **(b)** cells was evaluated using matrigel-based transwell assay (*n* = 3). The data are presented as the means ± SDs. ****P* < 0.001.


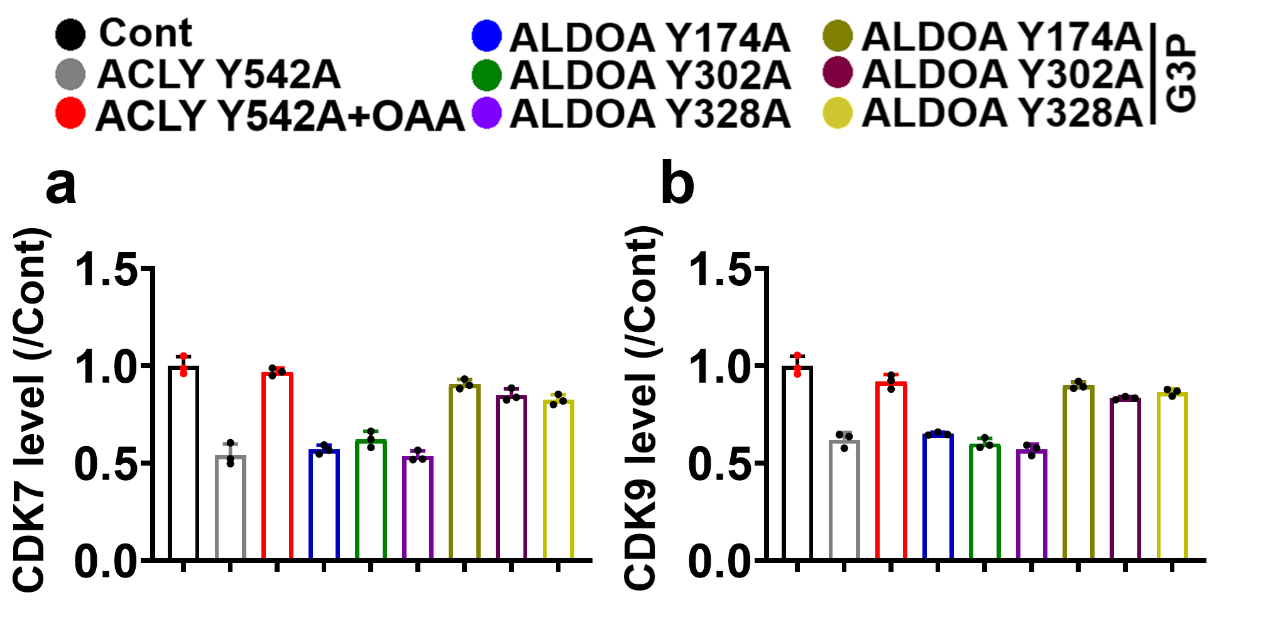


**Supplementary Fig. 8 ACLY and ALDOA tyrosine phosphorylation mediate the expression of CDK7 and CDK9 in primary ESCC cells**

**a-b** Quantitative ELISA assay was used to assess the protein of CDK7 **(a)** and CDK9 **(b)** in primary KYSE410 cells harboring ACLY Y542A with or without OAA (1 μM), ALDOA Y174A, Y302A, or Y328A with or without G3P (1 μM) (*n* = 3). The data are presented as the means ± SDs.


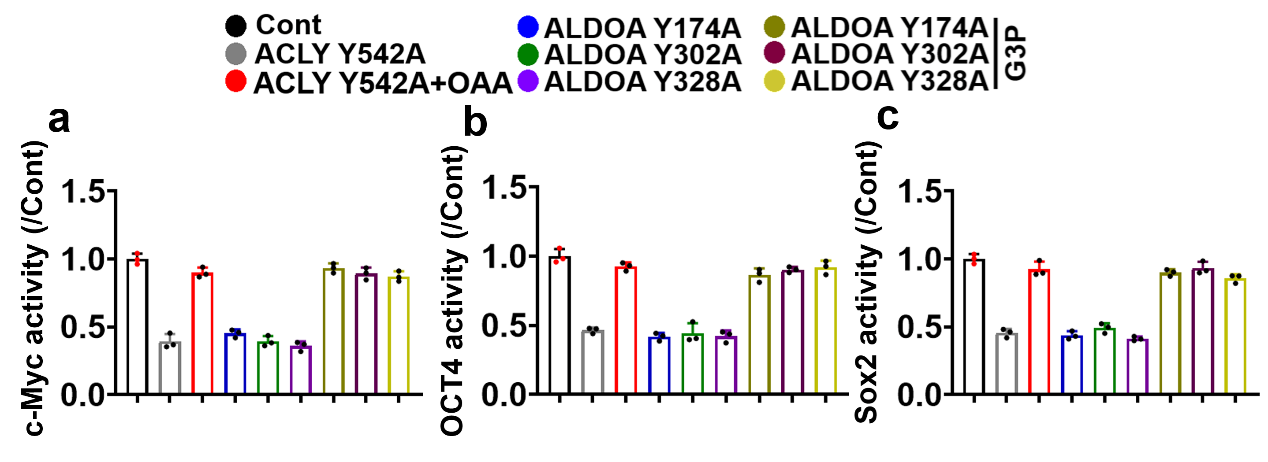


**Supplementary Fig. 9 ACLY and ALDOA tyrosine phosphorylation mediate the activity of c-Myc, Oct4, and Sox2 in metastatic ESCC cells**

**a-c** Nuclear lysates were obtained from metastatic KYSE410 cells, and the transcriptional activity of c-Myc **(a)**, Oct4 **(b)**, or Sox2 **(c)** was assessed using transcriptional activity assay (*n* = 3). The data are presented as the means ± SDs.


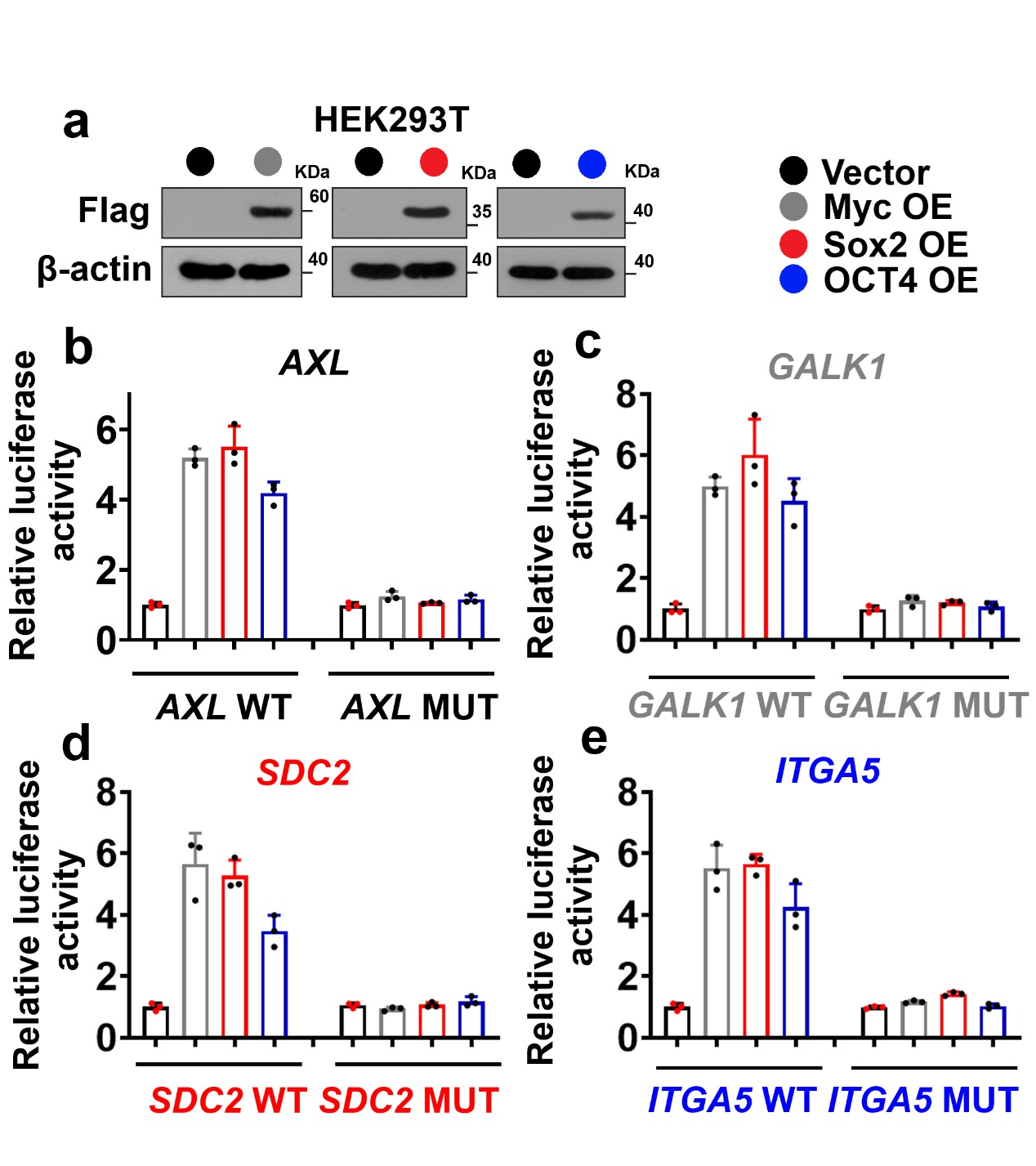


**Supplementary Fig. 10 c-Myc, Oct4, and Sox2 activate *AXL*, *GALK1*, *SDC2*, *ITGA5***

**a** Luciferase reporter assays in HEK293T cells transfected with indicated plasmids. Transfection efficacy was assessed using immunoblotting. **b-e** The luciferase activity of *AXL* **(b)**, *GALK1* **(c)**, *SDC2* **(d)**, *ITGA5* **(e)** in HEK293T cells was evaluated using dual-luciferase reporter assay (*n* = 3). The data are presented as the means ± SDs.


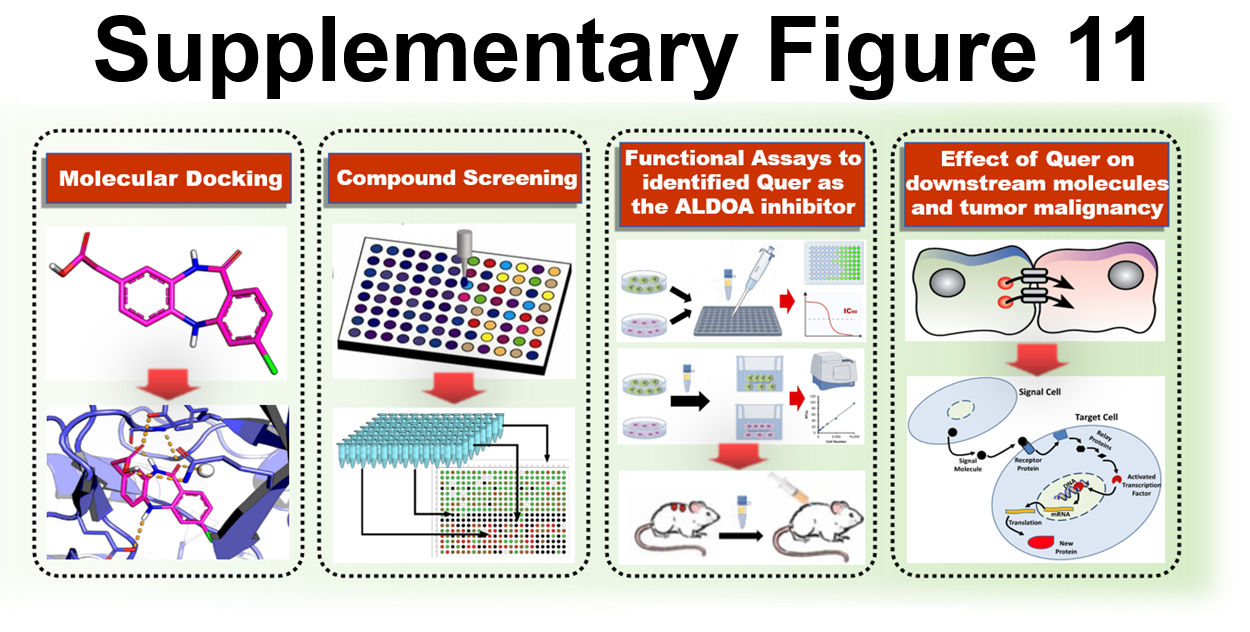


**Supplementary Fig. 11 Diagram showing the approach and protocol to screen ALDOA inhibitor**

Molecular docking, compound screening and functional assays identify quercetagitrin as ALDOA inhibitor, molecular biological experiments are applied to explore relevant mechanisms. Adobe Illustrator 2022 software is applied to draw Supplementary Fig. 11.


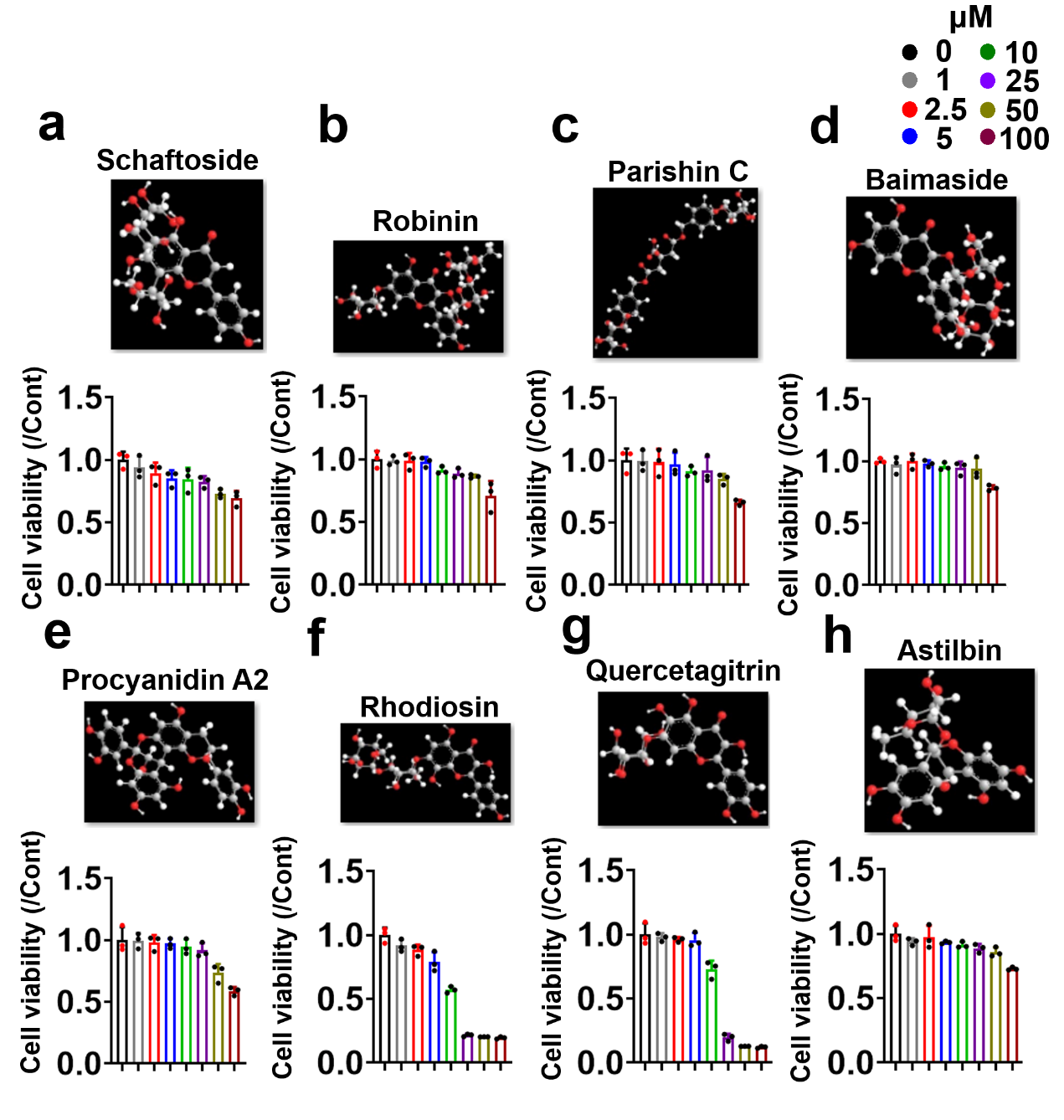


**Supplementary Fig. 12 Effect of potential ALDOA-targeted compounds on the growth of ESCC cells**

**a-h** The effect of schaftoside **(a)**, robinin **(b)**, parishin C **(c)**, baimaside **(d)**, procyanidin A2 **(e)**, rhodiosin **(f)**, quercetagitrin **(g)**, or astilbin **(h)** (0-100 μM) on the growth of KYSE510 cell evaluated using MTS assay (*n* = 3). The data are presented as the means ± SDs.


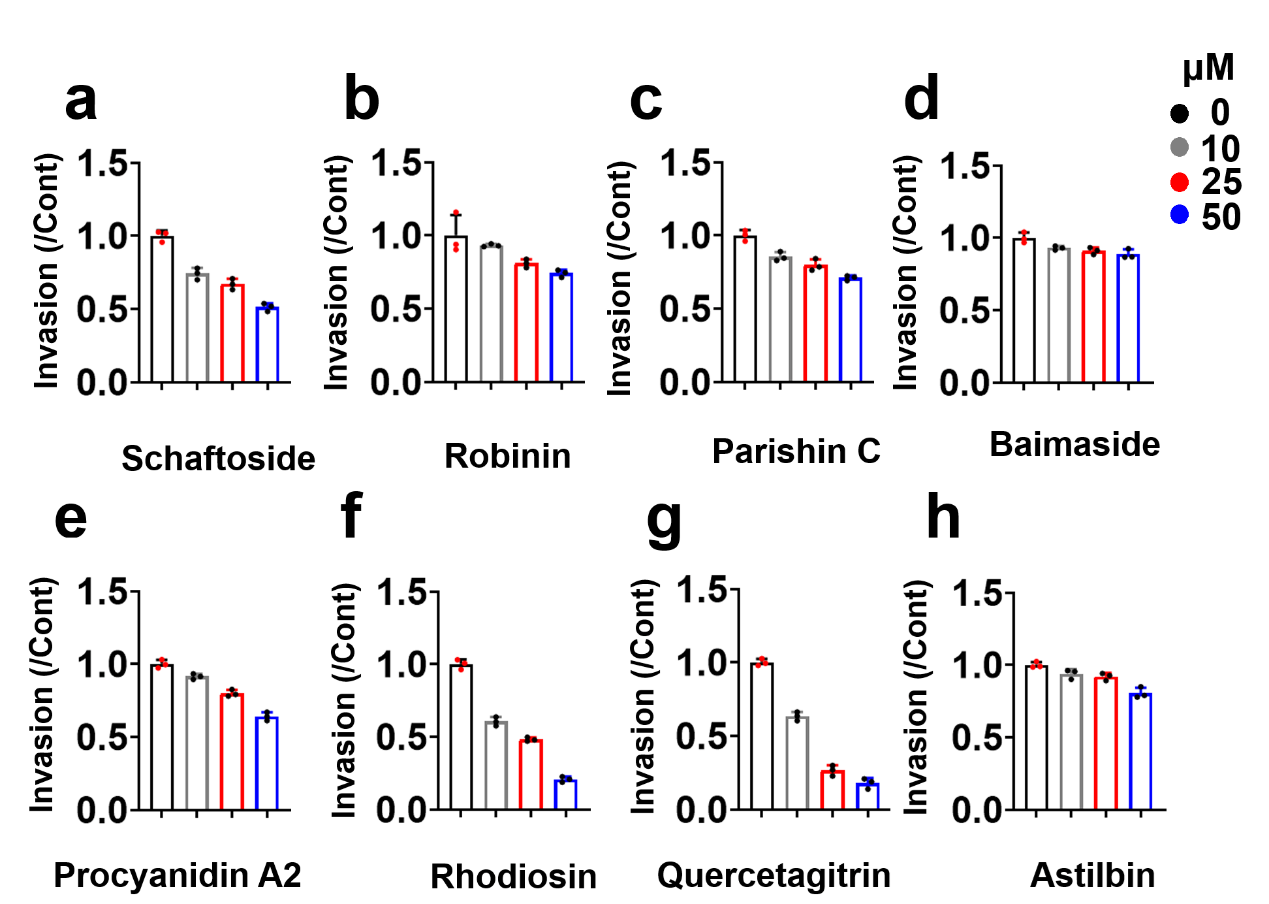


**Supplementary Fig. 13 Effect of potential ALDOA-targeted compounds on the invasion of ESCC cells**

**a-h** The effect of schaftoside **(a)**, robinin **(b)**, parishin C **(c)**, baimaside **(d)**, procyanidin A2 **(e)**, rhodiosin **(f)**, quercetagitrin **(g)**, or astilbin **(h)** (0, 10, 25, 50 μM) on the invasion of KYSE510 cell evaluated using matrigel-based transwell assay (*n* = 3). The data are presented as the means ± SDs.


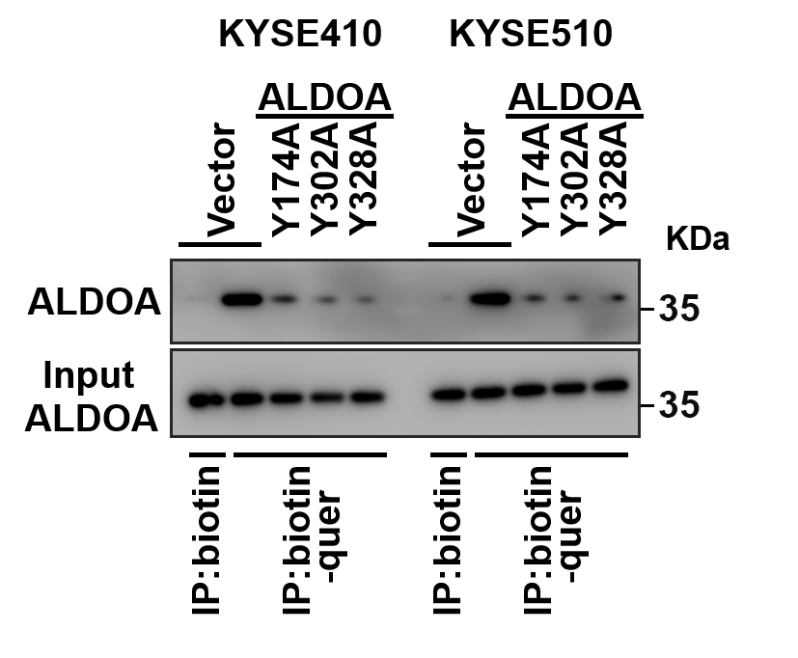


**Supplementary Fig. 14 Interaction status between quercetagitrin and ALDOA in ESCC cells harboring ALDOA mutants**

The streptavidin-pull down assay was applied to observe the interaction between biotin- quercetagitrin and ALDOA using immunoblotting with ALDOA antibody in KYSE410 and KYSE510 cells harboring ALDOA Y174A, Y302A, or Y328A.


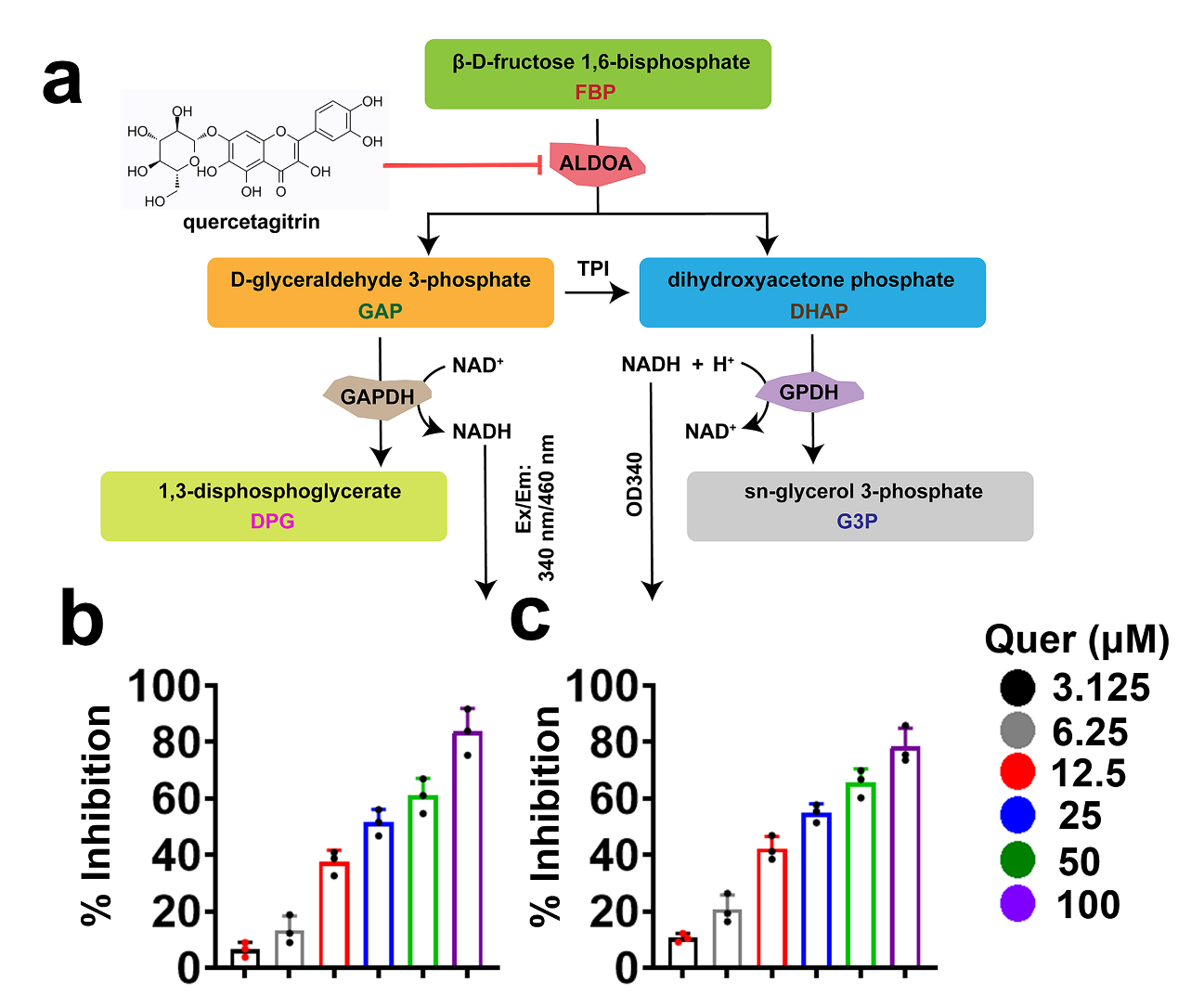


**Supplementary Fig. 15 Quercetagitrin inhibits the activity of recombinant human ALDOA activity in *in vitro* system**

**a** Schematic diagram for observing quercetagitrin-inhibited ALDOA activity. Adobe Illustrator 2022 software is applied to draw Supplementary Fig. 15a. **b** ALDOA activity was evaluated using GAPDH-produced NADH (*n* = 3). **c** ALDOA activity was evaluated using GPDH enzyme, and observed the remaining NADH level (*n* = 3). The data are presented as the means ± SDs.


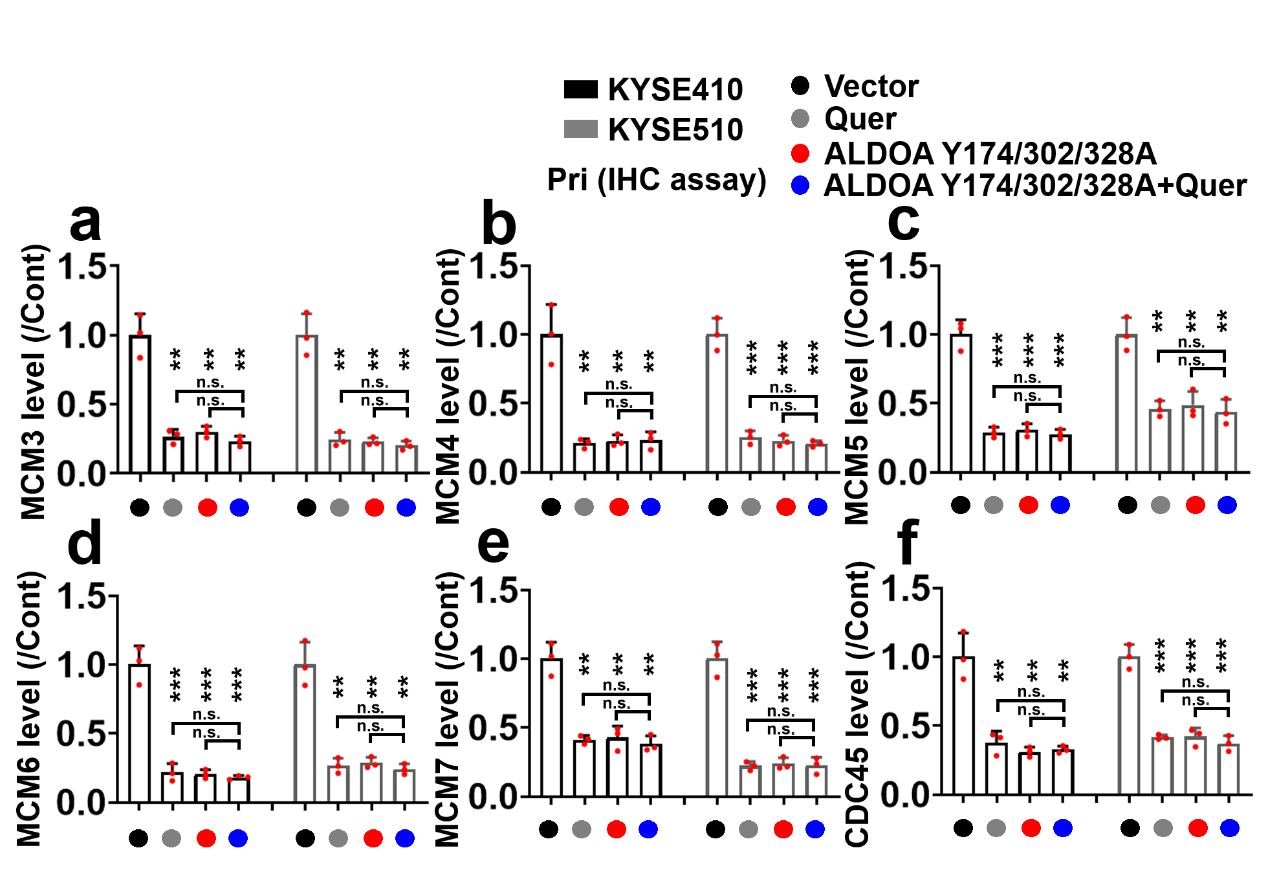


**Supplementary Fig. 16 Quercetagitrin inhibits the expression of MCMs/CDC45 complex in primary ESCC tumors**

**a-f** Statistical analysis of MCM3 **(a)**, MCM4 **(b)**, MCM5 **(c)**, MCM6 **(d)**, MCM7 **(e)**, or CDC45 **(f)** in Fig. 7c (*n* = 3). The data are presented as the means ± SDs. n.s., not significantly different; ***P* <0.01; ****P* < 0.001.


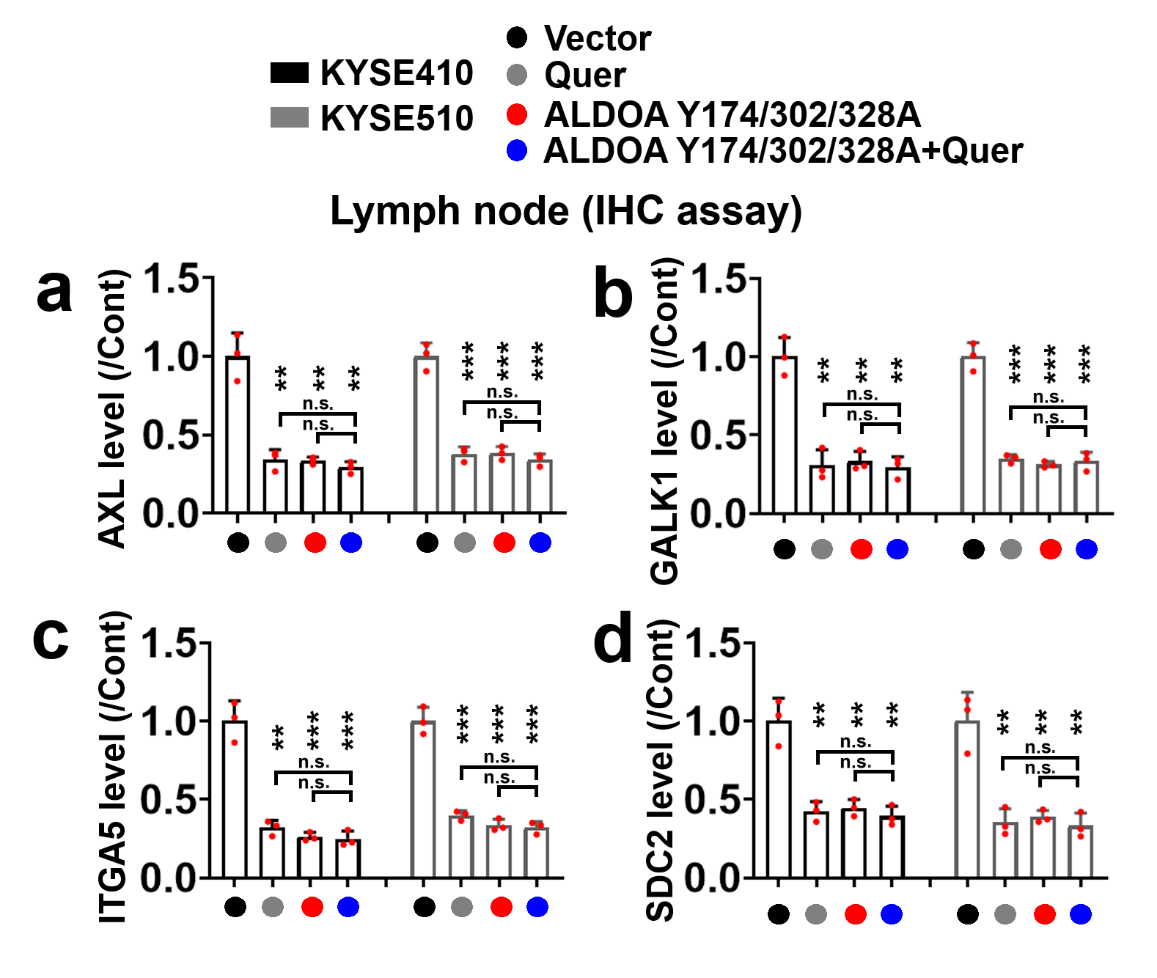


**Supplementary Fig. 17 Quercetagitrin inhibits the expression of AXL, GALK1, ITGA5, or SDC2 in lymph nodes**

**a-d** Statistical analysis of AXL **(a)**, GALK1 **(b)**, ITGA5 **(c)**, or SDC2 **(d)** in Fig. 7d (*n* = 3). The data are presented as the means ± SDs. n.s., not significantly different; ***P* <0.01; ****P* < 0.001.


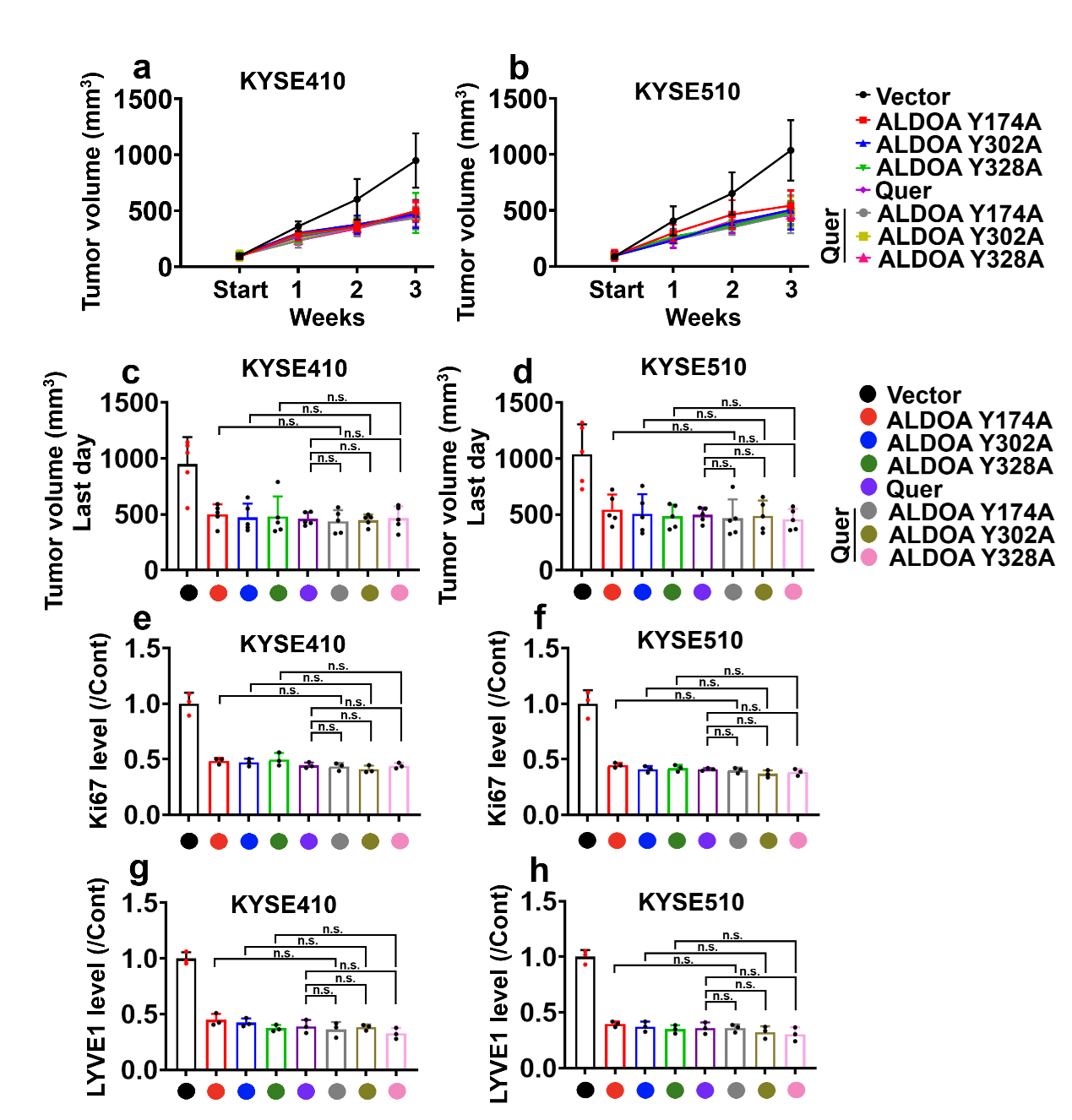


**Supplementary Fig. 18 ALDOA Tyr174, 302, or 328 critically contributes to** **quercetagitrin-mediated tumor malignancy inhibition**

**a-b** KYSE410 **(a)** and KYSE510 **(b)** cells harboring vector and ALDOA Y174A, Y302A, or Y328A mutants were respectively injected into the flank of BALB/c mouse. After tumor volume arrived at approximately 100 mm^3^. Mice were received with control vehicle or (*n* = 5; 25 mg/kg/day, p.o.) for 3 weeks. Tumor volume curves were listed. **c-d** The KYSE410 **(c)** and KYSE510 **(d)** tumor volumes at last day were indicated. Levels of intratumoral Ki67 **(e and f)** and LYVE1 **(g and h)** in KYSE410 **(e and g)** and KYSE510 **(f and h)** tumors were measured using quantitative ELISA assays (*n* = 3). The data are presented as the means ± SDs. n.s., not significantly different.


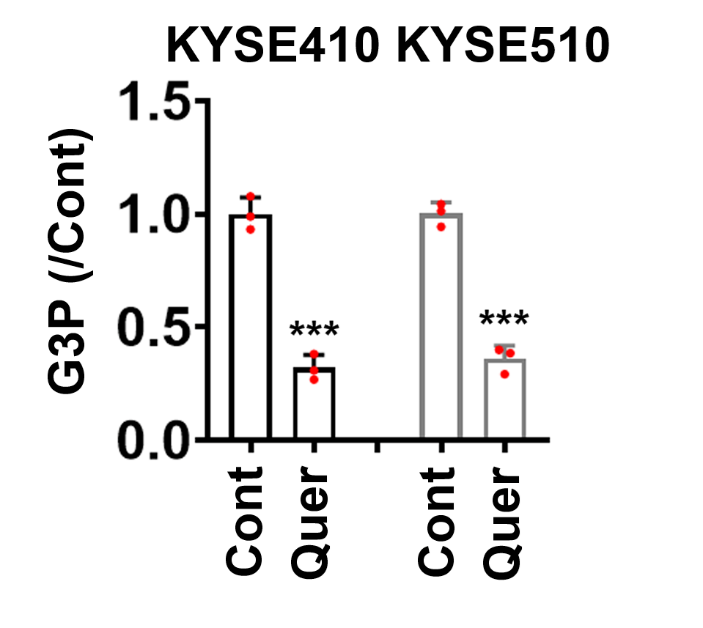


**Supplementary Fig. 19 Quercetagitrin inhibits the production of G3P in ESCC tumors**

KYSE410 and KYSE510 tumors were treated with control or quercetagitrin (25 mg/kg/day, p.o.). The tumor tissues were collected and lysates were subject to the G3P level measurement (*n* = 3). The data are presented as the means ± SDs. ****P* < 0.001.


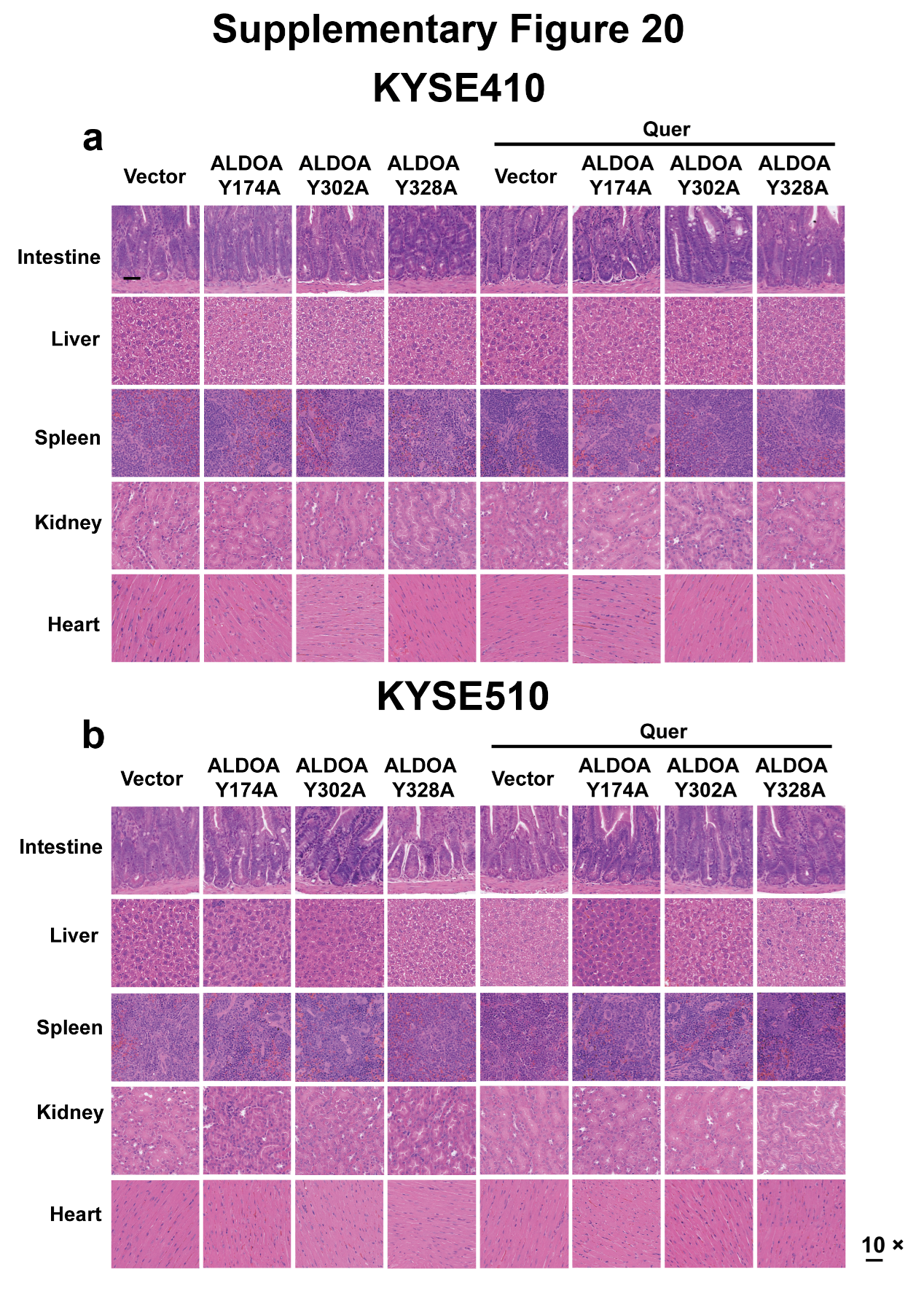


**Supplementary Fig. 20 Quercetagitrin produces no evidently systemic toxicity in xenograft model**

**a-b** H&E analyses of intestine, liver, spleen, kidney, and heart from animals harboring KYSE410 **(a)** and KYSE510 **(b)** tumors treated with control or quercetagitrin (25 mg/kg/day, p.o.). Magnification, 10 × as indicated.


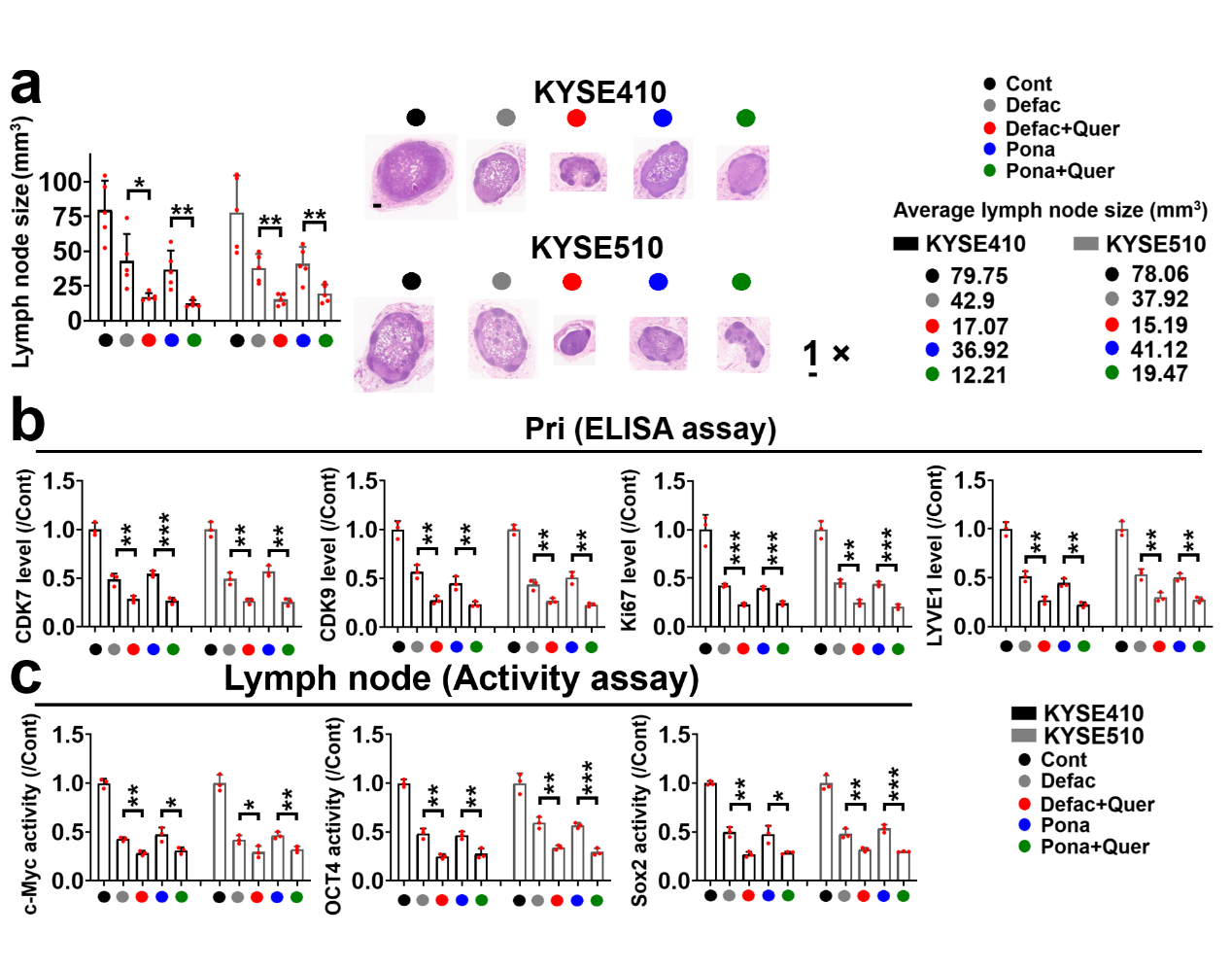


**Supplementary Fig. 21 Quercetagitrin synergizes with FAK/SFK inhibitors to block ESCC malignancy**

**a** Lymph node metastasis mouse model was established, and then the footpads of animals were injected with KYSE410 and KYSE510 cells. After approximately 1 week, animals were treated with defactinib (15 mg/kg/day, p.o.), or ponatinib (10 mg/kg/day, p.o.) alone or in the presence of quercetagitrin (25 mg/kg/day, p.o.) for 5 weeks. Lymph nodes were extracted and volumes of lymph nodes were calculated and quantified (*n* = 5). Representative images of H&E staining of lymph nodes were shown. Magnification, 1 × as indicated. **b** Protein lysates were extracted from primary ESCC tissues, and the level of CDK7, CDK9, Ki67, or LYVE1 was evaluated using quantitative ELISA assay (*n* = 3). **c** Nuclear lysates were obtained from lymph nodes, and the transcriptional activity of c-Myc, Oct4, or Sox2 was assessed using ELISA assay (*n* = 3). The data are presented as the means ± SDs. **P* < 0.05; ***P* <0.01; ****P* < 0.001.


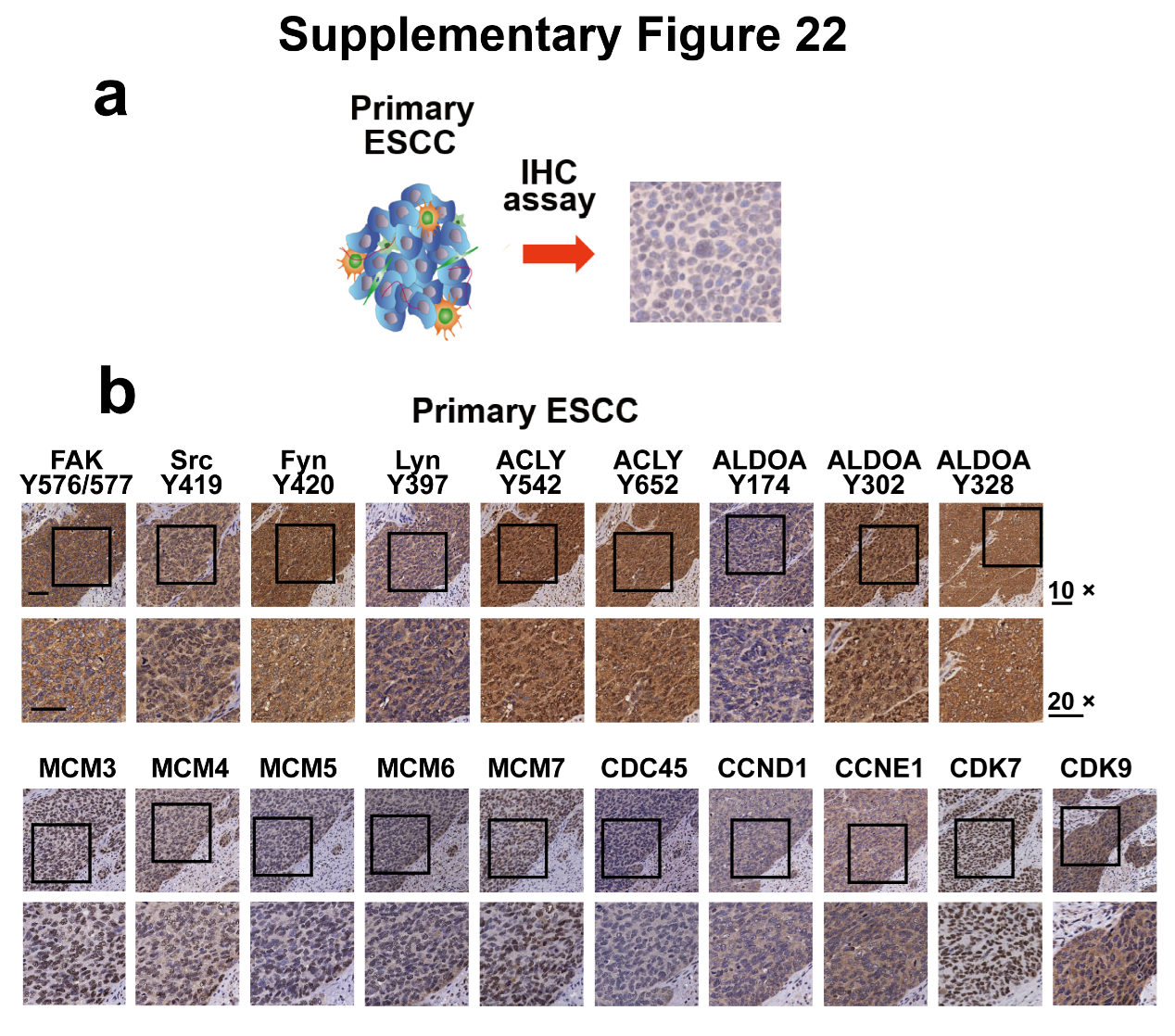


**Supplementary Fig. 22 FAK/SFK/ACLY/ALDOA axis is positively correlated with CDK7, CDK9 and MCMs/CDC45 complex in primary ESCC tissues**

**a** Protocol of IHC assay in primary ESCC. Adobe Illustrator 2022 software is applied to draw Supplementary Fig. 22a. **b** The expression correlation of pFAK Tyr576/577, pSrc Tyr419, pFyn Tyr420, pLyn Tyr397, pACLY Tyr542, Tyr652, and pALDOA Tyr174, Tyr302, or Tyr328 with MCM3, MCM4, MCM5, MCM6, MCM7, CDC45, CCND1, CCNE1, CDK7, and CDK9 in sequential slices of primary ESCC tissues (approximately *n* = 18). The representative specimens were shown. Magnification, 10 ×, 20 × as indicated. The statistical information was listed in Supplementary Table 4.


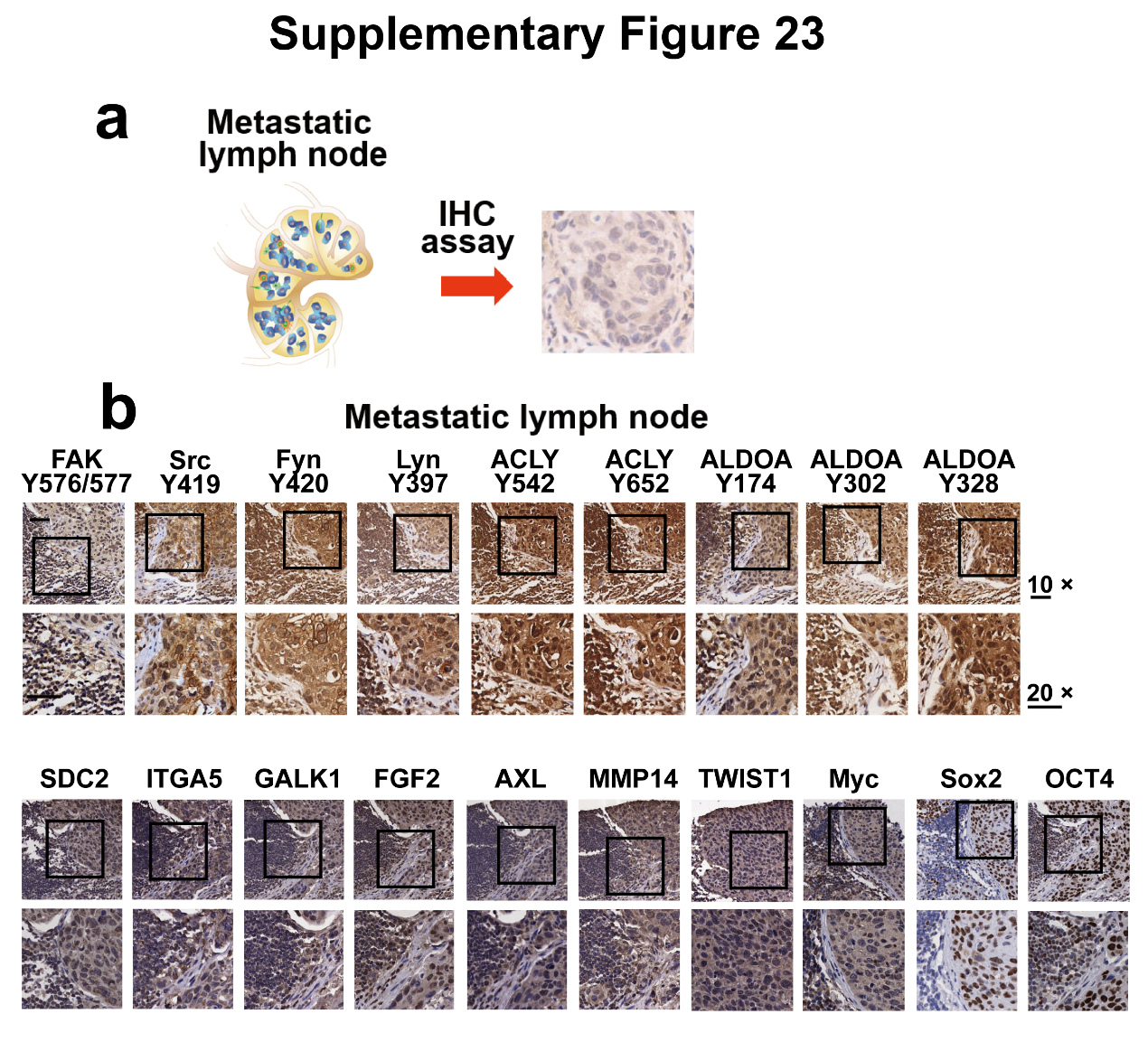


**Supplementary Fig. 23 FAK/SFK/ACLY/ALDOA axis is positively correlated with Yamanaka transcriptional factors and downstream effectors in metastatic ESCC tissues**

**a** Protocol of IHC assay in metastatic lymph node ESCC. Adobe Illustrator 2022 software is applied to draw Supplementary Fig. 23a. **b** Tyrosine phosphorylated FAK/SFK/ACLY/ALDOA axis was positively correlated with Yamanaka transcriptional factors and downstream molecules, including SDC2, ITGA5, GALK1, FGF2, AXL, MMP14, TWIST1, c-Myc, Sox2, and Oct4 in sequential slices of metastatic lymph nodes (approximately *n* = 20). The representative specimens were shown. Magnification, 10 ×, 20 × as indicated. The statistical information was listed in Supplementary Table 5.


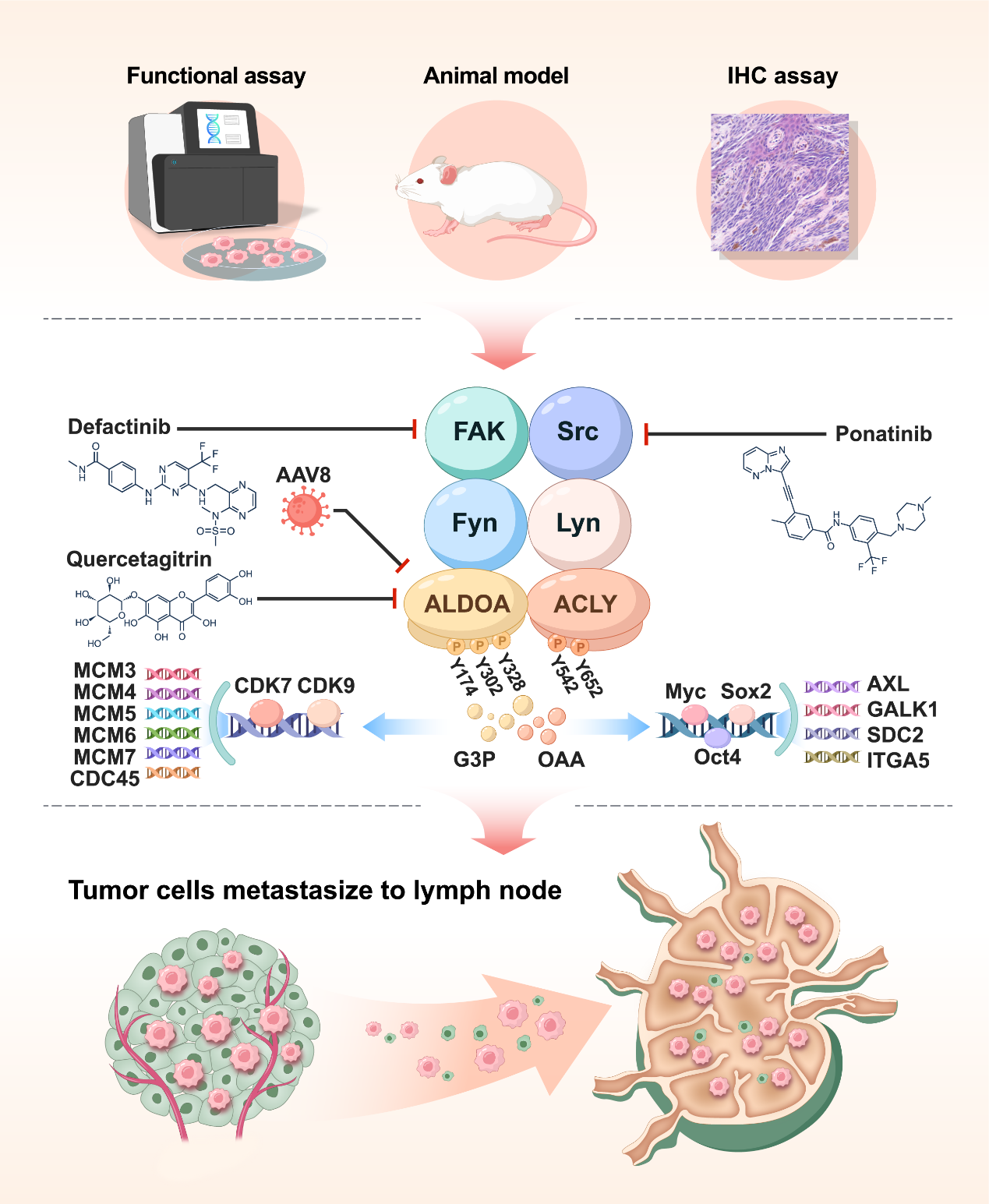


**Supplementary Figure 24. Proposed model of FAK/SFK axis phosphorylates tyrosine sites in ALDOA and ACLY to induce ESCC malignancy**

FAK/SFK axis phosphorylates the specific tyrosine sites in ACLY and ALDOA to induce the downstream reprogram, including facilitating CDK7/9-mediated the expression of MCMs/CDC45 complex in primary tumor cells, and stimulating transcriptional activity of Yamanaka factors to induce the expression of cell plasticity-related molecules in metastatic lymph nodes. Resultantly, FAK/SFK axis-phosphorylated ACLY and ALDOA induce the lymph node metastasis of ESCC cells. Adobe Illustrator 2022 software is applied to draw Supplementary Fig. 24.

**Supplementary Tables**

**Supplementary Table 1** **Pearson's correlation coefficient of FAK/SFK axis and ACLY, ALDOA in primary ESCC**

|  | **FAK Y576/577** | **Src Y419** | **Lyn Y397** | **Fyn Y420** |
| --- | --- | --- | --- | --- |
|  | **ALDOA Y174** | | | |
| **r** | 0.6541 | 0.813 | 0.6862 | 0.7655 |
| **P (two-tailed)** | 0.0032 | <0.0001 | 0.0017 | 0.0002 |
| **95% confidence interval** | 0.2695 to 0.8587 | 0.5578 to 0.9277 | 0.3227 to 0.8733 | 0.4647 to 0.9079 |
|  | **ALDOA Y302** | | | |
| **r** | 0.6027 | 0.7573 | 0.6951 | 0.633 |
| **P (two-tailed)** | 0.0063 | 0.0002 | 0.001 | 0.0036 |
| **95% confidence interval** | 0.2045 to 0.8298 | 0.4619 to 0.9014 | 0.3521 to 0.8735 | 0.2510 to 0.8444 |
|  | **ALDOA Y328** | | | |
| **r** | 0.7562 | 0.7694 | 0.7721 | 0.7752 |
| **P (two-tailed)** | 0.0002 | 0.0001 | 0.0001 | <0.0001 |
| **95% confidence interval** | 0.4600 to 0.9010 | 0.4845 to 0.9067 | 0.4895 to 0.9079 | 0.4953 to 0.9092 |
|  | **ACLY Y542** | | | |
| **r** | 0.6159 | 0.4514 | 0.571 | 0.5692 |
| **P (two-tailed)** | 0.0065 | 0.06 | 0.0133 | 0.0137 |
| **95% confidence interval** | 0.2092 to 0.8409 | -0.01958 to 0.7584 | 0.1419 to 0.8194 | 0.1394 to 0.8186 |
|  | **ACLY Y652** | | | |
| **r** | 0.7157 | 0.6863 | 0.495 | 0.874 |
| **P (two-tailed)** | 0.0006 | 0.0012 | 0.0312 | <0.0001 |
| **95% confidence interval** | 0.3874 to 0.8829 | 0.3372 to 0.8695 | 0.05266 to 0.7750 | 0.6962 to 0.9508 |

**Supplementary Table 2 Pearson's correlation coefficient of FAK/SFK axis and ACLY, ALDOA in metastatic lymph node**

|  | **FAK Y576/577** | **Src Y419** | **Lyn Y397** | **Fyn Y420** |
| --- | --- | --- | --- | --- |
|  | **ALDOA Y174** | | | |
| **r** | 0.6337 | 0.6656 | 0.6982 | 0.6701 |
| **P (two-tailed)** | 0.0027 | 0.0014 | 0.0006 | 0.0012 |
| **95% confidence interval** | 0.2657 to 0.8405 | 0.3162 to 0.8560 | 0.3699 to 0.8715 | 0.3235 to 0.8581 |
|  | **ALDOA Y302** | | | |
| **r** | 0.8757 | 0.6565 | 0.86 | 0.6855 |
| **P (two-tailed)** | <0.0001 | 0.0017 | <0.0001 | 0.0009 |
| **95% confidence interval** | 0.7072 to 0.9501 | 0.3016 to 0.8516 | 0.6740 to 0.9435 | 0.3487 to 0.8655 |
|  | **ALDOA Y328** | | | |
| **r** | 0.8391 | 0.7729 | 0.8835 | 0.754 |
| **P (two-tailed)** | <0.0001 | <0.0001 | <0.0001 | 0.0001 |
| **95% confidence interval** | 0.6308 to 0.9346 | 0.5022 to 0.9057 | 0.7241 to 0.9533 | 0.4675 to 0.8972 |
|  | **ACLY Y542** | | | |
| **r** | 0.6488 | 0.729 | 0.7872 | 0.5417 |
| **P (two-tailed)** | 0.002 | 0.0003 | <0.0001 | 0.0136 |
| **95% confidence interval** | 0.2894 to 0.8479 | 0.4230 to 0.8858 | 0.5290 to 0.9120 | 0.1305 to 0.7939 |
|  | **ACLY Y652** | | | |
| **r** | 0.7774 | 0.76 | 0.7433 | 0.814 |
| **P (two-tailed)** | <0.0001 | 0.0001 | 0.0002 | <0.0001 |
| **95% confidence interval** | 0.5105 to 0.9077 | 0.4784 to 0.8999 | 0.4481 to 0.8923 | 0.5807 to 0.9238 |

**Supplementary Table 3 Screening potential ALDOA inhibitors**

| Item | Catalog No. in MCE | Drug Name | Docking score | CAS No. | Formula | Mw |
| --- | --- | --- | --- | --- | --- | --- |
| 1 | HY-N4150 | Quercetagitrin | -14.283 | 548-75-4 | C_21_H_20_O_13_ | 480.38 |
| 2 | HY-N6006 | 1,3,6-Tri-O-galloyl-beta-D-glucose | -13.081 | 18483-17-5 | C_27_H_24_O_18_ | 636.47 |
| 3 | HY-N5119 | Kaempferol-3-O-(2''-O-β-D-glucopyl)-β-D-rutinoside | -12.508 | 55696-58-7 | C_33_H_40_O_20_ | 756.66 |
| 4 | HY-N1346 | Robinin | -12.209 | 301-19-9 | C_33_H_40_O_19_ | 740.66 |
| 5 | HY-N0522 | (-)-Gallocatechin gallate | -12.163 | 4233-96-9 | C_22_H_18_O_11_ | 458.37 |
| 6 | HY-N6937 | (R,R)-Secoisolariciresinol diglucoside | -12.080 | 158932-33-3 | C_32_H_46_O_16_ | 686.70 |
| 7 | HY-B1228 | Ribostamycin (sulfate) | -11.922 | 53797-35-6 | C_17_H_36_N_4_O_14_S | 454.47 |
| 8 | HY-N0712 | Typhaneoside | -11.871 | 104472-68-6 | C_34_H_42_O_20_ | 770.69 |
| 9 | HY-N2125 | Parishin C | -11.802 | 174972-80-6 | C_32_H_40_O_19_ | 728.65 |
| 10 | HY-N2425 | Rhodiosin | -11.716 | 86831-54-1 | C_27_H_30_O_16_ | 610.52 |
| 11 | HY-N2343 | Procyanidin A2 | -11.655 | 41743-41-3 | C_30_H_24_O_12_ | 576.50 |
| 12 | HY-N0244 | Theaflavin-3'-gallate | -11.601 | 28543-07-9 | C_36_H_28_O_16_ | 716.60 |
| 13 | HY-N6880 | Rabdosiin | -11.546 | 263397-69-9 | C_36_H_30_O_16_ | 718.61 |
| 14 | HY-N0509 | Astilbin | -11.497 | 29838-67-3 | C_21_H_22_O_11_ | 450.39 |
| 15 | HY-N0703 | Schaftoside | -11.453 | 51938-32-0 | C_26_H_28_O_14_ | 564.49 |
| 16 | HY-N2524 | Camelliaside A | -11.394 | 135095-52-2 | C_33_H_40_O_20_ | 756.66 |
| 17 | HY-125818 | Cytidine-5'-triphosphate | -11.393 | 65-47-4 | C_9_H_16_N_3_O_14_P_3_ | 483.16 |
| 18 | HY-107372 | Uridine triphosphate | -11.369 | 63-39-8 | C_9_H_15_N_2_O_15_P_3_ | 484.14 |
| 19 | HY-N2183 | Baimaside | -11.270 | 18609-17-1 | C_27_H_30_O_17_ | 626.52 |
| 20 | HY-13653 | (-)-Epigallocatechin Gallate | -11.259 | 989-51-5 | C_22_H_18_O_11_ | 458.37 |
| 21 | HY-N0152 | Myricitrin | -10.981 | 17912-87-7 | C_21_H_20_O_12_ | 464.38 |
| 22 | HY-N6617 | Norswertianolin | -10.962 | 54954-12-0 | C_19_H_18_O_11_ | 422.34 |
| 23 | HY-N0058 | 4,5-Dicaffeoylquinic acid | -10.782 | 57378-72-0 | C_25_H_24_O_12_ | 516.45 |
| 24 | HY-N3031 | Grosvenorine | -10.730 | 156980-60-8 | C_33_H_40_O_19_ | 740.66 |
| 25 | HY-B0509B | Amikacin (disulfate) | -10.679 | 39831-55-5 | C_22_H_47_N_5_O_21_S_2_ | 585.60 |
| 26 | HY-N0119 | Naringin Dihydrochalcone | -10.659 | 18916-17-1 | C_27_H_34_O_14_ | 582.55 |
| 27 | HY-N6895 | Violanthin | -10.637 | 40581-17-7 | C_27_H_30_O_14_ | 578.52 |
| 28 | HY-N1362 | Salvianolic acid B | -10.570 | 121521-90-2 | C_36_H_30_O_16_ | 718.61 |
| 29 | HY-B0470 | Neomycin (sulfate) | -10.530 | 1405-10-3 | C_23_H_52_N_6_O_25_S_3_ | 614.64 |
| 30 | HY-N0526 | 2"-O-Galloylhyperin | -10.519 | 53209-27-1 | C_28_H_24_O_16_ | 616.48 |
| 31 | HY-101886 | 5-BrUTP sodium salt | -10.509 | 161848-60-8 | C_9_H_14_BrN_2_O_15_P_3_.xNa | 563.04 |
| 32 | HY-17624A | Framycetin (sulfate) | -10.501 | 4146-30-9 | C_23_H_52_N_6_O_25_S_3_ | 614.64 |
| 33 | HY-N0356 | (-)-Catechin gallate | -10.493 | 130405-40-2 | C_22_H_18_O_10_ | 442.37 |
| 34 | HY-N2394 | Emodin-1-O-β-D-glucopyranoside | -10.393 | 38840-23-2 | C_21_H_20_O_10_ | 432.38 |
| 35 | HY-108869 | Fodipir | -10.338 | 118248-91-2 | C_22_H_32_N_4_O_14_P_2_ | 638.46 |
| 36 | HY-105008 | Secoisolariciresinol diglucoside | -10.336 | 257930-74-8 | C_32_H_46_O_16_ | 686.70 |
| 37 | HY-125112 | Vicenin-1 | -10.329 | 35927-38-9 | C_26_H_28_O_14_ | 564.49 |
| 38 | HY-135867E | NHC-triphosphate (tetraammonium) | -10.202 |  | C_9_H_28_N_7_O_15_P_3_ | 499.16 |
| 39 | HY-N4099 | Luteolin-3-O-beta-D-glucuronide | -10.149 | 53527-42-7 | C_21_H_18_O_12_ | 462.36 |
| 40 | HY-113325 | NADP | -10.107 | 53-59-8 | C_21_H_28_N_7_O_17_P_3_ | 743.41 |
| 41 | HY-N2192 | Swertianolin | -10.065 | 23445-00-3 | C_20_H_20_O_11_ | 436.37 |
| 42 | HY-101895 | Fluorescein di(β-D-galactopyranoside) | -9.959 | 17817-20-8 | C_32_H_32_O_15_ | 656.59 |
| 43 | HY-110135 | NBI-31772 | -9.943 | 374620-70-9 | C_17_H_11_NO_7_ | 341.27 |
| 44 | HY-N2397 | 9''-Methyl salvianolate B | -9.926 | 1167424-31-8 | C_37_H_32_O_16_ | 732.64 |
| 45 | HY-N0055 | Chlorogenic acid | -9.901 | 327-97-9 | C_16_H_18_O_9_ | 354.31 |
| 46 | HY-N2224 | Guaijaverin | -9.799 | 22255-13-6 | C_20_H_18_O_11_ | 434.35 |
| 47 | HY-N0652 | 2,3,5,4'-Tetrahydroxystilbene 2-O-β-D-glucoside | -9.751 | 82373-94-2 | C_20_H_22_O_9_ | 406.38 |
| 48 | HY-N0222 | Avicularin | -9.726 | 572-30-5 | C_20_H_18_O_11_ | 434.35 |
| 49 | HY-N0539 | Calceolarioside B | -9.575 | 105471-98-5 | C_23_H_26_O_11_ | 478.45 |
| 50 | HY-N0112 | Dihydromyricetin | -9.535 | 27200-12-0 | C_15_H_12_O_8_ | 320.25 |
| 51 | HY-126585 | SAICAR | -9.518 | 3031-95-6 | C_13_H_19_N_4_O_12_P | 454.28 |
| 52 | HY-10444 | R-1479 | -9.469 | 478182-28-4 | C_9_H_12_N_6_O_5_ | 284.23 |
| 53 | HY-N1510 | Kaempferol 3-O-gentiobioside | -9.437 | 22149-35-5 | C_27_H_30_O_16_ | 610.52 |
| 54 | HY-119580 | Leucocyanidin | -9.402 | 480-17-1 | C_15_H_14_O_7_ | 306.27 |
| 55 | HY-N9397 | Quercetin 3-O-(6''-O-malonyl)-β-D-glucoside | -9.378 | 96862-01-0 | C_24_H_22_O_15_ | 550.42 |
| 56 | HY-N1996 | Chebulagic acid | -9.360 | 23094-71-5 | C_41_H_30_O_27_ | 954.66 |
| 57 | HY-N0057 | 3,4-Dicaffeoylquinic acid | -9.354 | 14534-61-3 | C_25_H_24_O_12_ | 516.45 |
| 58 | HY-N0521 | (-)-Gallocatechin | -9.351 | 3371-27-5 | C_15_H_14_O_7_ | 306.27 |
| 59 | HY-100973A | Adenosine 5'-diphosphoribose (sodium) | -9.329 | 68414-18-6 | C_15_H_22_N_5_NaO_14_P_2_ | 558.31 |
| 60 | HY-129283A | Goralatide (TFA) | -9.297 | 1796568-94-9 | C_22_H_34_F_3_N_5_O_11_ | 487.50 |
| 61 | HY-N4090 | Vicenin 3 | -9.272 | 59914-91-9 | C_26_H_28_O_14_ | 564.49 |
| 62 | HY-W010918 | Adenosine 5'-diphosphate | -9.193 | 58-64-0 | C_10_H_15_N_5_O_10_P_2_ | 427.20 |
| 63 | HY-N5116 | Neoisoastilbin | -9.186 | 54141-72-9 | C_21_H_22_O_11_ | 450.39 |
| 64 | HY-125286 | AB-680 | -9.143 | 2105904-82-1 | C_20_H_24_ClFN_4_O_9_P_2_ | 580.82 |
| 65 | HY-N0794 | Proanthocyanidins | -9.070 | 20347-71-1 | C_30_H_26_O_13_ | 594.52 |
| 66 | HY-N2807 | Acanthoside B | -9.032 | 7374-79-0 | C_28_H_36_O_13_ | 580.58 |
| 67 | HY-100589 | Isepamicin (sulfate) | -8.974 | 67814-76-0 | C_22_H_43_N_5_O_12_.xH_2_SO_4_ | 569.60 |
| 68 | HY-N4100 | Trilobatin | -8.969 | 4192-90-9 | C_21_H_24_O_10_ | 436.41 |
| 69 | HY-P1868A | α2β1 Integrin Ligand Peptide (TFA) | -8.898 |  | C_16_H_23_F_3_N_4_O_11_ | 390.35 |
| 70 | HY-B0028 | Fludarabine (phosphate) | -8.858 | 75607-67-9 | C_10_H_13_FN_5_O_7_P | 365.21 |
| 71 | HY-N8321 | 5-O-Caffeoylshikimic acid | -8.820 | 73263-62-4 | C_16_H_16_O_8_ | 336.29 |
| 72 | HY-N0619 | Mulberroside A | -8.811 | 102841-42-9 | C_26_H_32_O_14_ | 568.52 |
| 73 | HY-N0651 | Spinosin | -8.798 | 72063-39-9 | C_28_H_32_O_15_ | 608.54 |
| 74 | HY-107780 | Cyclic-di-GMP | -8.790 | 61093-23-0 | C_20_H_24_N_10_O_14_P_2_ | 690.41 |
| 75 | HY-107777 | LLY-283 | -8.715 | 2040291-27-6 | C_17_H_18_N_4_O_4_ | 342.35 |
| 76 | HY-N4073 | 6"-O-Malonyldaidzin | -8.711 | 124590-31-4 | C_24_H_22_O_12_ | 502.42 |
| 77 | HY-107202 | Polyinosinic-polycytidylic acid | -8.683 | 24939-03-5 | (C_10_H_13_N_4_O_8_P)x.(C_9_H_14_N_3_O_8_P)x | 348.21 |
| 78 | HY-B0277 | Vidarabine | -8.662 | 5536-17-4 | C_10_H_13_N_5_O_4_ | 267.24 |
| 79 | HY-N0567 | Hydroxysafflor yellow A | -8.648 | 78281-02-4 | C_27_H_32_O_16_ | 612.53 |
| 80 | HY-P1043A | NGR peptide (Trifluoroacetate) | -8.640 |  | C_22_H_37_F_3_N_10_O_10_S_2_ | 608.69 |
| 81 | HY-12290 | Arg-Gly-Asp-Ser | -8.600 | 91037-65-9 | C_15_H_27_N_7_O_8_ | 433.42 |
| 82 | HY-119917 | Gossypetin | -8.583 | 489-35-0 | C_15_H_10_O_8_ | 318.24 |
| 83 | HY-N0532 | Morroniside | -8.570 | 25406-64-8 | C_17_H_26_O_11_ | 406.38 |
| 84 | HY-135780A | 3'-Deoxyuridine-5'-triphosphate (trisodium) | -8.561 |  | C_9_H_15_N_2_Na_3_O_14_P_3_ | 468.14 |
| 85 | HY-N6868 | Dimethyl lithospermate B | -8.536 | 875313-64-7 | C_38_H_34_O_16_ | 746.67 |
| 86 | HY-136497 | Coenzyme FO | -8.506 | 37333-48-5 | C_16_H_17_N_3_O_7_ | 363.32 |
| 87 | HY-N1425 | Tiliroside | -8.479 | 20316-62-5 | C_30_H_26_O_13_ | 594.52 |
| 88 | HY-135897 | Urolithin C | -8.403 | 165393-06-6 | C_13_H_8_O_5_ | 244.20 |
| 89 | HY-A0181 | Adenosine monophosphate | -8.343 | 61-19-8 | C_10_H_14_N_5_O_7_P | 347.22 |
| 90 | HY-113061 | Pseudouridine | -8.324 | 1445-07-4 | C_9_H_12_N_2_O_6_ | 244.20 |
| 91 | HY-N0002 | (-)-Epicatechin gallate | -8.321 | 1257-08-5 | C_22_H_18_O_10_ | 442.37 |
| 92 | HY-112639 | Aristeromycin | -8.123 | 19186-33-5 | C_11_H_15_N_5_O_3_ | 265.27 |
| 93 | HY-W010861 | 2'-Deoxycytidine-5'-diphosphate (trisodium) | -8.121 | 151151-32-5 | C_9_H_12_N_3_Na_3_O_10_P_2_ | 384.15 |
| 94 | HY-18644 | CWHM-12 | -8.081 | 1564286-55-0 | C_26_H_32_BrN_5_O_6_ | 590.47 |
| 95 | HY-P1740A | RGD peptide (GRGDNP) (TFA) | -8.077 |  | C_25_H_39_F_3_N_10_O_12_ | 614.61 |
| 96 | HY-N7664 | Isotheaflavin | -8.069 | 31701-93-6 | C_29_H_24_O_12_ | 564.49 |
| 97 | HY-N0006 | Demethoxycurcumin | -8.041 | 22608-11-3 | C_20_H_18_O_5_ | 338.35 |
| 98 | HY-101943 | LY 345899 | -8.002 | 10538-99-5 | C_20_H_21_N_7_O_7_ | 471.42 |
| 99 | HY-10820 | Pemetrexed | -7.934 | 137281-23-3 | C_20_H_21_N_5_O_6_ | 427.41 |
| 100 | HY-B0203A | Nebivolol (hydrochloride) | -7.905 | 152520-56-4 | C_22_H_26_ClF_2_NO_4_ | 405.44 |
| 101 | HY-131592 | Tricetin | -7.877 | 520-31-0 | C_15_H_10_O_7_ | 302.24 |
| 102 | HY-N0240 | Herbacetin | -7.869 | 527-95-7 | C_15_H_10_O_7_ | 302.24 |
| 103 | HY-N0418 | Quercitrin | -7.866 | 522-12-3 | C_21_H_20_O_11_ | 448.38 |
| 104 | HY-125989 | 2-Methylthio-AMP | -7.863 | 22140-20-1 | C_11_H_16_N_5_O_7_PS | 393.31 |
| 105 | HY-19528 | SAH | -7.817 | 979-92-0 | C_14_H_20_N_6_O_5_S | 384.41 |
| 106 | HY-B1087 | Prednisolone (hemisuccinate) | -7.619 | 2920-86-7 | C_25_H_32_O_8_ | 460.52 |
| 107 | HY-N6071 | Secoisolariciresinol | -7.444 | 29388-59-8 | C_20_H_26_O_6_ | 362.42 |
| 108 | HY-N4095 | Brevifolincarboxylic acid | -7.431 | 18490-95-4 | C_13_H_8_O_8_ | 292.20 |
| 109 | HY-N0604 | Ginsenoside Rh1 | -7.391 | 63223-86-9 | C_36_H_62_O_9_ | 638.87 |
| 110 | HY-19738 | NQTrp | -7.380 | 185351-19-3 | C_21_H_16_N_2_O_4_ | 360.36 |
| 111 | HY-N0072 | Brazilin | -7.380 | 474-07-7 | C_16_H_14_O_5_ | 286.28 |
| 112 | HY-N2101 | Benzoyloxypaeoniflorin | -7.363 | 72896-40-3 | C_30_H_32_O_13_ | 600.57 |
| 113 | HY-N0182 | Fisetin | -7.341 | 528-48-3 | C_15_H_10_O_6_ | 286.24 |
| 114 | HY-145443 | Fludarabine-Cl | -7.340 | 2734853-80-4 | C_10_H_11_ClFN_5_O_3_ | 303.68 |
| 115 | HY-10586 | 5-Azacytidine | -7.336 | 320-67-2 | C_8_H_12_N_4_O_5_ | 244.20 |
| 116 | HY-W009216 | 2'-Deoxycytidine-5'-monophosphoric acid | -7.312 | 1032-65-1 | C_9_H_14_N_3_O_7_P | 307.20 |
| 117 | HY-N7138 | 2-(Phosphonooxy)benzoic acid | -7.192 | 6064-83-1 | C_7_H_7_O_6_P | 218.10 |
| 118 | HY-103173 | 5'-N-Ethylcarboxamidoadenosine | -7.169 | 35920-39-9 | C_12_H_16_N_6_O_4_ | 308.29 |
| 119 | HY-B0025 | Voglibose | -7.167 | 83480-29-9 | C_10_H_21_NO_7_ | 267.28 |
| 120 | HY-A0032A | Valganciclovir (hydrochloride) | -7.121 | 175865-59-5 | C_14_H_23_ClN_6_O_5_ | 354.36 |
| 121 | HY-B0148 | Risedronic acid | -7.115 | 105462-24-6 | C_7_H_11_NO_7_P_2_ | 283.11 |
| 122 | HY-13623A | Entecavir (monohydrate) | -7.112 | 209216-23-9 | C_12_H_17_N_5_O_4_ | 277.28 |
| 123 | HY-N6741 | β-Zearalenol | -7.109 | 71030-11-0 | C_18_H_24_O_5_ | 320.38 |
| 124 | HY-119751 | Hematein | -7.059 | 475-25-2 | C_16_H_12_O_6_ | 300.26 |
| 125 | HY-13573 | Biapenem | -7.052 | 120410-24-4 | C_15_H_18_N_4_O_4_S | 350.39 |
| 126 | HY-101097 | PD-1-IN-17 | -7.021 | 1673560-66-1 | C_13_H_22_N_6_O_7_ | 374.35 |
| 127 | HY-16563 | Narciclasine | -7.019 | 29477-83-6 | C_14_H_13_NO_7_ | 307.26 |
| 128 | HY-N1347 | Robinetin | -6.975 | 490-31-3 | C_15_H_10_O_7_ | 302.24 |
| 129 | HY-N0621 | Morin | -6.905 | 480-16-0 | C_15_H_10_O_7_ | 302.24 |
| 130 | HY-W009162 | Cytidine 5'-monophosphate | -6.897 | 63-37-6 | C_9_H_14_N_3_O_8_P | 323.20 |
| 131 | HY-143207 | 10-Formyl-5,8-dideazafolic acid | -6.894 | 61038-31-1 | C_22_H_21_N_5_O_7_ | 467.43 |
| 132 | HY-N2093 | Vicine | -6.878 | 152-93-2 | C_10_H_16_N_4_O_7_ | 304.26 |
| 133 | HY-112730 | PEO-IAA | -6.849 | 6266-66-6 | C_18_H_15_NO_3_ | 293.32 |
| 134 | HY-N6714 | Alternariol | -6.841 | 641-38-3 | C_14_H_10_O_5_ | 258.23 |
| 135 | HY-A0006 | Pentostatin | -6.818 | 53910-25-1 | C_11_H_16_N_4_O_4_ | 268.27 |
| 136 | HY-19168 | Perzinfotel | -6.765 | 144912-63-0 | C_9_H_13_N_2_O_5_P | 260.18 |
| 137 | HY-N0225 | (-)-Epigallocatechin | -6.758 | 970-74-1 | C_15_H_14_O_7_ | 306.27 |
| 138 | HY-138598 | 5'-O-TBDMS-dG | -6.757 | 51549-33-8 | C_16_H_27_N_5_O_4_Si | 381.50 |
| 139 | HY-N4142 | Cyanidin-3-O-galactoside (chloride) | -6.664 | 27661-36-5 | C_21_H_21_ClO_11_ | 449.38 |
| 140 | HY-W077589 | 3-(5-(Aminomethyl)-1-oxoisoindolin-2-yl)piperidine-2,6-dione hydrochloride | -6.655 | 1158264-69-7 | C_14_H_16_ClN_3_O_3_ | 273.29 |
| 141 | HY-B1402 | Hydrocortisone hemisuccinate | -6.639 | 2203-97-6 | C_25_H_34_O_8_ | 462.53 |
| 142 | HY-13417 | AICAR | -6.605 | 2627-69-2 | C_9_H_14_N_4_O_5_ | 258.23 |
| 143 | HY-B0021 | Doxifluridine | -6.605 | 3094-09-5 | C_9_H_11_FN_2_O_5_ | 246.19 |
| 144 | HY-14814A | Delafloxacin (meglumine) | -6.570 | 352458-37-8 | C_25_H_29_ClF_3_N_5_O_9_ | 440.76 |
| 145 | HY-112861A | Gln-AMS (TFA) | -6.565 |  | C_17_H_23_F_3_N_8_O_10_S | 474.45 |
| 146 | HY-W039892 | 4-Nitrophenyl β-D-glucuronide | -6.557 | 10344-94-2 | C_12_H_13_NO_9_ | 315.23 |
| 147 | HY-13518 | Piceatannol | -6.548 | 10083-24-6 | C_14_H_12_O_4_ | 244.24 |
| 148 | HY-131394 | 5-Hydroxymethyl-2'-deoxycytidine | -6.535 | 7226-77-9 | C_10_H_15_N_3_O_5_ | 257.24 |
| 149 | HY-106263B | Tyroserleutide (hydrochloride) | -6.508 | 852982-42-4 | C_18_H_28_ClN_3_O_6_ | 381.42 |
| 150 | HY-15205 | Ganetespib | -6.507 | 888216-25-9 | C_20_H_20_N_4_O_3_ | 364.40 |
| 151 | HY-115688 | TXNIP-IN-1 | -6.499 | 1268955-50-5 | C_12_H_12_N_2_O_4_ | 248.23 |
| 152 | HY-N7400 | Phaseoloidin | -6.491 | 118555-82-1 | C_14_H_18_O_9_ | 330.29 |
| 153 | HY-12312 | TCS 401 | -6.480 | 243966-09-8 | C_10_H_11_ClN_2_O_5_S | 270.26 |
| 154 | HY-10644 | Lck inhibitor 2 | -6.473 | 944795-06-6 | C_18_H_17_N_5_O_2_ | 335.36 |
| 155 | HY-15097 | Myricetin | -6.459 | 529-44-2 | C_15_H_10_O_8_ | 318.24 |
| 156 | HY-B1158 | Imidazolidinyl urea | -6.454 | 39236-46-9 | C_11_H_16_N_8_O_8_ | 388.29 |
| 157 | HY-77839 | Cortodoxone | -6.444 | 152-58-9 | C_21_H_30_O_4_ | 346.46 |
| 158 | HY-122381 | Kyotorphin | -6.419 | 70904-56-2 | C_15_H_23_N_5_O_4_ | 337.37 |
| 159 | HY-111444 | Auxinole | -6.386 | 86445-22-9 | C_20_H_19_NO_3_ | 321.37 |
| 160 | HY-N3755 | Dihydroresveratrol | -6.371 | 58436-28-5 | C_14_H_14_O_3_ | 230.26 |
| 161 | HY-N0519 | Calycosin | -6.362 | 20575-57-9 | C_16_H_12_O_5_ | 284.26 |
| 162 | HY-B1449 | Uridine | -6.341 | 58-96-8 | C_9_H_12_N_2_O_6_ | 244.20 |
| 163 | HY-17622 | Tavilermide | -6.326 | 263251-78-1 | C_24_H_32_N_6_O_11_ | 580.54 |
| 164 | HY-B1832 | Prednisone acetate | -6.312 | 125-10-0 | C_23_H_28_O_6_ | 400.46 |
| 165 | HY-N0792 | Tectorigenin | -6.306 | 548-77-6 | C_16_H_12_O_6_ | 300.26 |
| 166 | HY-112582 | N1-Methylpseudouridine | -6.305 | 13860-38-3 | C_10_H_14_N_2_O_6_ | 258.23 |
| 167 | HY-108251 | Methotrexate metabolite | -6.291 | 19741-14-1 | C_15_H_15_N_7_O_2_ | 325.33 |
| 168 | HY-16420 | R112 | -6.287 | 575474-82-7 | C_16_H_13_FN_4_O_2_ | 312.30 |
| 169 | HY-19332 | Kifunensine | -6.284 | 109944-15-2 | C_8_H_12_N_2_O_6_ | 232.19 |
| 170 | HY-113291 | 5'-Deoxyadenosine | -6.255 | 4754-39-6 | C_10_H_13_N_5_O_3_ | 251.24 |
| 171 | HY-128578 | KPLH1130 | -6.242 | 906669-07-6 | C_15_H_13_N_3_O_3_ | 283.28 |
| 172 | HY-B1493 | Bentiromide | -6.242 | 37106-97-1 | C_23_H_20_N_2_O_5_ | 404.42 |
| 173 | HY-B0648 | Medroxyprogesterone | -6.229 | 520-85-4 | C_22_H_32_O_3_ | 344.49 |
| 174 | HY-15847 | HS38 | -6.225 | 1030203-81-6 | C_14_H_12_ClN_5_O_2_S | 349.80 |
| 175 | HY-121629 | PS210 | -6.217 | 1221962-86-2 | C_19_H_15_F_3_O_5_ | 380.31 |
| 176 | HY-14648A | Dexamethasone acetate | -6.210 | 1177-87-3 | C_24_H_31_FO_6_ | 434.50 |
| 177 | HY-N0898A | (-)-Catechin | -6.150 | 18829-70-4 | C_15_H_14_O_6_ | 290.27 |
| 178 | HY-N5072 | Desmethylglycitein | -6.131 | 17817-31-1 | C_15_H_10_O_5_ | 270.24 |
| 179 | HY-P0002A | Protirelin (acetate) | -6.108 | 120876-23-5 | C_16_H_22_N_6_O_4_ | 362.38 |
| 180 | HY-15440A | Fostemsavir | -6.093 | 864953-29-7 | C_25_H_26_N_7_O_8_P | 583.49 |
| 181 | HY-135842 | Aspoxicillin | -6.075 | 63358-49-6 | C_21_H_27_N_5_O_7_S | 493.53 |
| 182 | HY-111645 | 3-Methylcytidine | -6.072 | 2140-64-9 | C_10_H_15_N_3_O_5_ | 257.24 |
| 183 | HY-15167 | Glyoxalase I inhibitor | -6.053 | 221174-33-0 | C_21_H_30_BrClN_4_O_8_S | 577.45 |
| 184 | HY-115688A | (S)-TXNIP-IN-1 | -6.031 | 1212421-96-9 | C_12_H_12_N_2_O_4_ | 248.23 |
| 185 | HY-N2495 | Isomucronulatol | -6.025 | 52250-35-8 | C_17_H_18_O_5_ | 302.32 |
| 186 | HY-N0162 | Luteolin | -6.000 | 491-70-3 | C_15_H_10_O_6_ | 286.24 |
| 187 | HY-B0506 | Nadifloxacin | -5.975 | 124858-35-1 | C_19_H_21_FN_2_O_4_ | 360.38 |
| 188 | HY-P0299A | LSKL, Inhibitor of Thrombospondin (TSP-1) (TFA) | -5.968 |  | C_23_H_43_F_3_N_6_O_7_ | 458.60 |
| 189 | HY-116217 | 5-Fluoro-2'-deoxycytidine | -5.961 | 10356-76-0 | C_9_H_12_FN_3_O_4_ | 245.21 |
| 190 | HY-B1182 | Chromocarb | -5.935 | 4940-39-0 | C_10_H_6_O_4_ | 190.15 |
| 191 | HY-B0243 | Meprednisone | -5.913 | 1247-42-3 | C_22_H_28_O_5_ | 372.45 |
| 192 | HY-N4075 | O-Desmethylangolensin | -5.903 | 21255-69-6 | C_15_H_14_O_4_ | 258.27 |
| 193 | HY-129997 | Luteolinidin (chloride) | -5.896 | 1154-78-5 | C_15_H_11_ClO_5_ | 271.24 |
| 194 | HY-B1203A | Fludrocortisone acetate | -5.881 | 514-36-3 | C_23_H_31_FO_6_ | 422.49 |
| 195 | HY-W092109 | H-Phe-Trp-OH | -5.857 | 24587-41-5 | C_20_H_21_N_3_O_3_ | 351.40 |
| 196 | HY-N0898 | Catechin | -5.746 | 154-23-4 | C_15_H_14_O_6_ | 290.27 |
| 197 | HY-Y0057 | NSC 13138 | -5.735 | 486-74-8 | C_10_H_7_NO_2_ | 173.17 |
| 198 | HY-41982 | D-Glucuronic acid lactone | -5.726 | 32449-92-6 | C_6_H_8_O_6_ | 176.12 |
| 199 | HY-N6670 | Cefotetan | -5.701 | 69712-56-7 | C_17_H_17_N_7_O_8_S_4_ | 575.62 |
| 200 | HY-113313 | Aldosterone | -5.689 | 52-39-1 | C_21_H_28_O_5_ | 360.44 |

**Supplementary Table 4 Pearson's correlation coefficient of FAK/SFK/ACLY and ALDOA axis and downstream effectors in primary ESCC**

|  | **FAK Y576/577** | **Src Y419** | **Fyn Y420** | **Lyn Y397** | **ACLY Y542** | **ACLY Y652** | **ALDOA Y174** | **ALDOA Y302** | **ALDOA Y328** |
| --- | --- | --- | --- | --- | --- | --- | --- | --- | --- |
|  | **MCM3** | | | | | | | | |
| **r** | 0.7565 | 0.6907 | 0.7837 | 0.6194 | 0.6462 | 0.7504 | 0.6618 | 0.7207 | 0.8788 |
| **P (two-tailed)** | 0.0004 | 0.0021 | 0.0002 | 0.008 | 0.0051 | 0.0005 | 0.0038 | 0.0011 | <0.0001 |
| **95% confidence interval** | 0.4334 to 0.9072 | 0.3144 to 0.8794 | 0.4862 to 0.9184 | 0.1976 to 0.8477 | 0.2402 to 0.8598 | 0.4219 to 0.9047 | 0.2657 to 0.8667 | 0.3672 to 0.8923 | 0.6894 to 0.9558 |
|  | **MCM4** | | | | | | | | |
| **r** | 0.7242 | 0.4556 | 0.6748 | 0.5341 | 0.5186 | 0.6498 | 0.56 | 0.649 | 0.6913 |
| **P (two-tailed)** | 0.001 | 0.0661 | 0.003 | 0.0272 | 0.0329 | 0.0048 | 0.0194 | 0.0048 | 0.0021 |
| **95% confidence interval** | 0.3736 to 0.8937 | -0.03204 to 0.7681 | 0.2873 to 0.8725 | 0.07188 to 0.8074 | 0.05060 to 0.7999 | 0.2460 to 0.8614 | 0.1085 to 0.8199 | 0.2447 to 0.8610 | 0.3155 to 0.8797 |
|  | **MCM5** | | | | | | | | |
| **r** | 0.5584 | 0.5313 | 0.6657 | 0.6429 | 0.6396 | 0.5617 | 0.4855 | 0.472 | 0.6088 |
| **P (two-tailed)** | 0.016 | 0.0233 | 0.0026 | 0.004 | 0.0043 | 0.0153 | 0.0411 | 0.048 | 0.0073 |
| **95% confidence interval** | 0.1239 to 0.8133 | 0.08566 to 0.7998 | 0.2885 to 0.8640 | 0.2515 to 0.8536 | 0.2463 to 0.8520 | 0.1286 to 0.8149 | 0.02413 to 0.7764 | 0.006566 to 0.7693 | 0.1983 to 0.8376 |
|  | **MCM6** | | | | | | | | |
| **r** | 0.7218 | 0.5509 | 0.765 | 0.7043 | 0.6884 | 0.7318 | 0.5382 | 0.5952 | 0.6856 |
| **P (two-tailed)** | 0.0007 | 0.0178 | 0.0002 | 0.0011 | 0.0016 | 0.0006 | 0.0212 | 0.0092 | 0.0017 |
| **95% confidence interval** | 0.3845 to 0.8891 | 0.1131 to 0.8096 | 0.4638 to 0.9077 | 0.3537 to 0.8814 | 0.3264 to 0.8743 | 0.4024 to 0.8934 | 0.09528 to 0.8032 | 0.1777 to 0.8311 | 0.3216 to 0.8730 |
|  | **MCM7** | | | | | | | | |
| **r** | 0.6595 | 0.6262 | 0.6801 | 0.6272 | 0.5833 | 0.6113 | 0.5656 | 0.5312 | 0.718 |
| **P (two-tailed)** | 0.0029 | 0.0054 | 0.0019 | 0.0053 | 0.0111 | 0.007 | 0.0144 | 0.0233 | 0.0008 |
| **95% confidence interval** | 0.2783 to 0.8612 | 0.2252 to 0.8458 | 0.3125 to 0.8706 | 0.2268 to 0.8463 | 0.1600 to 0.8254 | 0.2021 to 0.8388 | 0.1342 to 0.8168 | 0.08553 to 0.7997 | 0.3777 to 0.8874 |
|  | **CDC45** | | | | | | | | |
| **r** | 0.5303 | 0.6554 | 0.7353 | 0.6641 | 0.5829 | 0.7886 | 0.6407 | 0.6034 | 0.7074 |
| **P (two-tailed)** | 0.0236 | 0.0031 | 0.0005 | 0.0027 | 0.0111 | 0.0001 | 0.0042 | 0.008 | 0.001 |
| **95% confidence interval** | 0.08431 to 0.7993 | 0.2717 to 0.8593 | 0.4087 to 0.8949 | 0.2858 to 0.8633 | 0.1594 to 0.8252 | 0.5091 to 0.9176 | 0.2481 to 0.8526 | 0.1901 to 0.8350 | 0.3591 to 0.8827 |
|  | **CCND1** | | | | | | | | |
| **r** | 0.8758 | 0.6387 | 0.9053 | 0.639 | 0.5587 | 0.7827 | 0.6301 | 0.6479 | 0.7593 |
| **P (two-tailed)** | <0.0001 | 0.0043 | <0.0001 | 0.0043 | 0.0159 | 0.0001 | 0.0051 | 0.0036 | 0.0003 |
| **95% confidence interval** | 0.6918 to 0.9530 | 0.2448 to 0.8516 | 0.7593 to 0.9645 | 0.2454 to 0.8518 | 0.1242 to 0.8134 | 0.4977 to 0.9151 | 0.2312 to 0.8476 | 0.2595 to 0.8559 | 0.4530 to 0.9052 |
|  | **CCNE1** | | | | | | | | |
| **r** | 0.6808 | 0.6143 | 0.7499 | 0.5754 | 0.5109 | 0.7391 | 0.5335 | 0.5581 | 0.683 |
| **P (two-tailed)** | 0.0019 | 0.0067 | 0.0003 | 0.0125 | 0.0302 | 0.0005 | 0.0226 | 0.0161 | 0.0018 |
| **95% confidence interval** | 0.3136 to 0.8709 | 0.2067 to 0.8402 | 0.4356 to 0.9013 | 0.1483 to 0.8216 | 0.05789 to 0.7895 | 0.4156 to 0.8966 | 0.08871 to 0.8009 | 0.1234 to 0.8131 | 0.3173 to 0.8719 |
|  | **CDK7** | | | | | | | | |
| **r** | 0.5897 | 0.4544 | 0.7229 | 0.5181 | 0.3783 | 0.5978 | 0.588 | 0.433 | 0.4894 |
| **P (two-tailed)** | 0.0079 | 0.0507 | 0.0005 | 0.0231 | 0.1216 | 0.0069 | 0.0103 | 0.0641 | 0.0335 |
| **95% confidence interval** | 0.1850 to 0.8234 | 0.0001848 to 0.7531 | 0.4000 to 0.8861 | 0.08351 to 0.7871 | -0.1075 to 0.7183 | 0.1971 to 0.8274 | 0.1670 to 0.8277 | -0.02644 to 0.7414 | 0.04523 to 0.7720 |
|  | **CDK9** | | | | | | | | |
| **r** | 0.621 | 0.5775 | 0.7115 | 0.7579 | 0.7049 | 0.6935 | 0.6835 | 0.7366 | 0.6872 |
| **P (two-tailed)** | 0.0059 | 0.0121 | 0.0009 | 0.0003 | 0.0011 | 0.0014 | 0.0018 | 0.0005 | 0.0016 |
| **95% confidence interval** | 0.2171 to 0.8434 | 0.1515 to 0.8226 | 0.3663 to 0.8845 | 0.4505 to 0.9047 | 0.3547 to 0.8816 | 0.3351 to 0.8766 | 0.3182 to 0.8721 | 0.4111 to 0.8955 | 0.3244 to 0.8737 |

**Supplementary Table 5 Pearson's correlation coefficient of FAK/SFK/ACLY and ALDOA axis and downstream effectors in metastatic lymph node**

|  | **FAK Y576/577** | **Src Y419** | **Fyn Y420** | **Lyn Y397** | **ACLY Y542** | **ACLY Y652** | **ALDOA Y174** | **ALDOA Y302** | **ALDOA Y328** |
| --- | --- | --- | --- | --- | --- | --- | --- | --- | --- |
|  | **SDC2** | | | | | | | | |
| **r** | 0.5791 | 0.6653 | 0.712 | 0.672 | 0.5807 | 0.6772 | 0.5782 | 0.6454 | 0.7061 |
| **P (two-tailed)** | 0.0118 | 0.0026 | 0.0009 | 0.0023 | 0.0115 | 0.002 | 0.012 | 0.0038 | 0.0011 |
| **95% confidence interval** | 0.1538 to 0.8233 | 0.2878 to 0.8639 | 0.3673 to 0.8848 | 0.2989 to 0.8669 | 0.1562 to 0.8241 | 0.3076 to 0.8693 | 0.1525 to 0.8229 | 0.2555 to 0.8547 | 0.3568 to 0.8821 |
|  | **ITGA5** | | | | | | | | |
| **r** | 0.6564 | 0.6168 | 0.4999 | 0.7995 | 0.6456 | 0.4742 | 0.6051 | 0.7164 | 0.6819 |
| **P (two-tailed)** | 0.0017 | 0.0038 | 0.0248 | <0.0001 | 0.0021 | 0.0346 | 0.0047 | 0.0004 | 0.0009 |
| **95% confidence interval** | 0.3015 to 0.8516 | 0.2398 to 0.8322 | 0.07370 to 0.7717 | 0.5525 to 0.9174 | 0.2843 to 0.8463 | 0.04012 to 0.7577 | 0.2221 to 0.8264 | 0.4010 to 0.8800 | 0.3428 to 0.8638 |
|  | **GALK1** | | | | | | | | |
| **r** | 0.5795 | 0.7306 | 0.6251 | 0.7291 | 0.5375 | 0.4116 | 0.6127 | 0.5972 | 0.7204 |
| **P (two-tailed)** | 0.0074 | 0.0003 | 0.0032 | 0.0003 | 0.0145 | 0.0714 | 0.0041 | 0.0054 | 0.0003 |
| **95% confidence interval** | 0.1842 to 0.8134 | 0.4257 to 0.8865 | 0.2523 to 0.8363 | 0.4232 to 0.8858 | 0.1247 to 0.7917 | -0.03786 to 0.7225 | 0.2335 to 0.8301 | 0.2102 to 0.8224 | 0.4079 to 0.8818 |
|  | **FGF2** | | | | | | | | |
| **r** | 0.6286 | 0.7637 | 0.7792 | 0.7033 | 0.6302 | 0.6059 | 0.6395 | 0.5688 | 0.7212 |
| **P (two-tailed)** | 0.003 | <0.0001 | <0.0001 | 0.0005 | 0.0029 | 0.0046 | 0.0024 | 0.0089 | 0.0003 |
| **95% confidence interval** | 0.2578 to 0.8380 | 0.4852 to 0.9016 | 0.5139 to 0.9085 | 0.3787 to 0.8739 | 0.2602 to 0.8388 | 0.2232 to 0.8268 | 0.2747 to 0.8434 | 0.1688 to 0.8080 | 0.4093 to 0.8822 |
|  | **AXL** | | | | | | | | |
| **r** | 0.6067 | 0.7757 | 0.6721 | 0.7225 | 0.6989 | 0.554 | 0.5178 | 0.5417 | 0.6434 |
| **P (two-tailed)** | 0.0046 | <0.0001 | 0.0012 | 0.0003 | 0.0006 | 0.0113 | 0.0193 | 0.0136 | 0.0022 |
| **95% confidence interval** | 0.2244 to 0.8271 | 0.5074 to 0.9069 | 0.3268 to 0.8591 | 0.4116 to 0.8828 | 0.3712 to 0.8718 | 0.1477 to 0.8003 | 0.09771 to 0.7813 | 0.1305 to 0.7939 | 0.2808 to 0.8453 |
|  | **MMP14** | | | | | | | | |
| **r** | 0.7 | 0.777 | 0.7459 | 0.7835 | 0.6109 | 0.6588 | 0.7921 | 0.7506 | 0.6782 |
| **P (two-tailed)** | 0.0006 | <0.0001 | 0.0002 | <0.0001 | 0.0042 | 0.0016 | <0.0001 | 0.0001 | 0.001 |
| **95% confidence interval** | 0.3730 to 0.8723 | 0.5098 to 0.9075 | 0.4528 to 0.8935 | 0.5219 to 0.9104 | 0.2307 to 0.8292 | 0.3053 to 0.8527 | 0.5383 to 0.9142 | 0.4613 to 0.8956 | 0.3367 to 0.8620 |
|  | **TWIST1** | | | | | | | | |
| **r** | 0.7618 | 0.7902 | 0.7326 | 0.8178 | 0.7146 | 0.6346 | 0.8293 | 0.8005 | 0.7185 |
| **P (two-tailed)** | 0.0002 | <0.0001 | 0.0004 | <0.0001 | 0.0006 | 0.0035 | <0.0001 | <0.0001 | 0.0005 |
| **95% confidence interval** | 0.4703 to 0.9034 | 0.5241 to 0.9157 | 0.4172 to 0.8905 | 0.5784 to 0.9275 | 0.3855 to 0.8824 | 0.2534 to 0.8452 | 0.6017 to 0.9323 | 0.5441 to 0.9201 | 0.3924 to 0.8842 |
|  | **Myc** | | | | | | | | |
| **r** | 0.7621 | 0.6757 | 0.6119 | 0.767 | 0.5655 | 0.5697 | 0.4931 | 0.6853 | 0.739 |
| **P (two-tailed)** | <0.0001 | 0.0011 | 0.0041 | <0.0001 | 0.0094 | 0.0087 | 0.0272 | 0.0009 | 0.0002 |
| **95% confidence interval** | 0.4822 to 0.9008 | 0.3327 to 0.8608 | 0.2323 to 0.8298 | 0.4912 to 0.9030 | 0.1640 to 0.8063 | 0.1700 to 0.8084 | 0.06467 to 0.7680 | 0.3484 to 0.8654 | 0.4406 to 0.8904 |
|  | **Sox2** | | | | | | | | |
| **r** | 0.5915 | 0.6253 | 0.6578 | 0.6445 | 0.637 | 0.5748 | 0.4393 | 0.5365 | 0.7055 |
| **P (two-tailed)** | 0.006 | 0.0032 | 0.0016 | 0.0022 | 0.0025 | 0.008 | 0.0527 | 0.0147 | 0.0005 |
| **95% confidence interval** | 0.2018 to 0.8195 | 0.2527 to 0.8364 | 0.3036 to 0.8522 | 0.2825 to 0.8458 | 0.2709 to 0.8421 | 0.1774 to 0.8110 | -0.004044 to 0.7383 | 0.1232 to 0.7912 | 0.3824 to 0.8749 |
|  | **OCT4** | | | | | | | | |
| **r** | 0.7408 | 0.7782 | 0.6433 | 0.819 | 0.7865 | 0.6898 | 0.6112 | 0.7537 | 0.7789 |
| **P (two-tailed)** | 0.0002 | <0.0001 | 0.0022 | <0.0001 | <0.0001 | 0.0008 | 0.0042 | 0.0001 | <0.0001 |
| **95% confidence interval** | 0.4437 to 0.8912 | 0.5120 to 0.9080 | 0.2807 to 0.8452 | 0.5905 to 0.9259 | 0.5276 to 0.9117 | 0.3559 to 0.8675 | 0.2313 to 0.8294 | 0.4670 to 0.8970 | 0.5133 to 0.9083 |

**Supplementary Table 6** **The primer sequences of indicated genes for ChIP assay**

| **Gene** | **Forward/Reverse** | **Primer sequence** |
| --- | --- | --- |
| ***MCM3*** | Forward | 5’-CCTATTGGTCAGTTTGCTCGTC-3’ |
|  | Reverse | 5’-GGCTGAGTTCTCTGAGGTCG-3’ |
| ***MCM4*** | Forward | 5’-AGCCGCAGCAGGGAGCAA-3’ |
|  | Reverse | 5’-TTCCTTAGGTTTCCATGTTGATTCG-3’ |
| ***MCM5*** | Forward | 5’-CCAAGAAAATAAAAAAGAAACCACCTA-3’ |
|  | Reverse | 5’-CGAAGATGATTGGCTGCAAAGT-3’ |
| ***MCM6*** | Forward | 5’-TCCAGCTCCTGTGTCACGATT-3’ |
|  | Reverse | 5’-CGCGAGGTCCATATTTGCTTAGT-3’ |
| ***MCM7*** | Forward | 5’-ATGGCCGTTCTGGAGAGTAGAC-3’ |
|  | Reverse | 5’-CGCACACGCGTTCTTTTGTTC-3’ |
| ***CDC45*** | Forward | 5’-TCCTCTCAGGCAATGCAACGA-3’ |
|  | Reverse | 5’-GGGTGTTTTAGAGCCTAGCGTCTT-3’ |
| ***AXL*** | Forward | 5’-TCTCTTGAGTTAACCCCTGATTGTC -3’ |
|  | Reverse | 5’-CTTCCCTCACTCCCAGACTTG -3’ |
| ***GALK1*** | Forward | 5’-TGGATTCCCACGGGAGTTGC-3’ |
|  | Reverse | 5’-CCTGGGGCTGTCTCAAAGCA-3’ |
| ***SDC2*** | Forward | 5’-GAGGGAGCCAGAGGAAAAGAAGA-3’ |
|  | Reverse | 5’-AAAGGGAGCTACGGCAGAAGTTTAG-3’ |
| ***ITGA5*** | Forward | 5’-GCCCAGTCTAACCCAGTCCAGA-3’ |
|  | Reverse | 5’-TTTGCCAAACTCCCGGCTGA-3’ |

**Supplementary Table 7 The primer sequences of indicated genes for RT-PCR**

| **Gene** | **Forward/Reverse** | **Primer sequence** |
| --- | --- | --- |
| ***MCM3*** | Forward | 5’-GCGCAGGAAAAACGAGAAGAG-3’ |
|  | Reverse | 5’-AATGGAGGCCACAAAATCCTTT-3’ |
| ***MCM4*** | Forward | 5’-TGAACCTCTATACATGCAACGAC-3’ |
|  | Reverse | 5’-CAGGGTAACGGTCAAAGAAGATT-3’ |
| ***MCM5*** | Forward | 5’-AGCATTCGTAGCCTGAAGTCG-3’ |
|  | Reverse | 5’-CGGCACTGGATAGAGATGCG-3’ |
| ***MCM6*** | Forward | 5’-TCGGGCCTTGAAAACATTCGT-3’ |
|  | Reverse | 5’-TGTGTCTGGTAGGCAGGTCTT-3’ |
| ***MCM7*** | Forward | 5’-GCCTGTGGGAAATATCCCTCG-3’ |
|  | Reverse | 5’-GTACCACCTGTCGGAACCC-3’ |
| ***CDC45*** | Forward | 5’-CTTGAAGTTCCCGCCTATGAAG-3’ |
|  | Reverse | 5’-GCATGGTTTGCTCCACTATCTC-3’ |
| ***CCND1*** | Forward | 5’-CAATGACCCCGCACGATTTC-3’ |
|  | Reverse | 5’-CATGGAGGGCGGATTGGAA-3’ |
| ***CCNE1*** | Forward | 5’-ACTCAACGTGCAAGCCTCG-3’ |
|  | Reverse | 5’-GCTCAAGAAAGTGCTGATCCC-3’ |
| ***CDK2*** | Forward | 5’-GTACCTCCCCTGGATGAAGAT-3’ |
|  | Reverse | 5’-CGAAATCCGCTTGTTAGGGTC-3’ |
| ***CDK6*** | Forward | 5’-TCTTCATTCACACCGAGTAGTGC-3’ |
|  | Reverse | 5’-TGAGGTTAGAGCCATCTGGAAA-3’ |
| ***IL6*** | Forward | 5’-ACTCACCTCTTCAGAACGAATTG-3’ |
|  | Reverse | 5’-CCATCTTTGGAAGGTTCAGGTTG-3’ |
| ***IL11*** | Forward | 5’-CGAGCGGACCTACTGTCCTA-3’ |
|  | Reverse | 5’-GCCCAGTCAAGTGTCAGGTG-3’ |
| ***AXL*** | Forward | 5’-GTGGGCAACCCAGGGAATATC-3’ |
|  | Reverse | 5’-GTACTGTCCCGTGTCGGAAAG-3’ |
| ***FGF2*** | Forward | 5’-AGTGTGTGCTAACCGTTACCT-3’ |
|  | Reverse | 5’-ACTGCCCAGTTCGTTTCAGTG-3’ |
| ***GALK1*** | Forward | 5’-ACCAGTTCATCTCACTTATGGGA-3’ |
|  | Reverse | 5’-AGTGGCGGACATTAGAGTTGG-3’ |
| ***HIF1A*** | Forward | 5’-ATCCATGTGACCATGAGGAAATG-3’ |
|  | Reverse | 5’-TCGGCTAGTTAGGGTACACTTC-3’ |
| ***ITGA5*** | Forward | 5’-GCCTGTGGAGTACAAGTCCTT-3’ |
|  | Reverse | 5’-AATTCGGGTGAAGTTATCTGTGG-3’ |
| ***MMP14*** | Forward | 5’-CGAGGTGCCCTATGCCTAC-3’ |
|  | Reverse | 5’-CTCGGCAGAGTCAAAGTGG-3’ |
| ***SDC2*** | Forward | 5’-TGGAAACCACGACGCTGAATA-3’ |
|  | Reverse | 5’-ATAACTCCACCAGCAATGACAG-3’ |
| ***TWIST1*** | Forward | 5’-GTCCGCAGTCTTACGAGGAG-3’ |
|  | Reverse | 5’-GCTTGAGGGTCTGAATCTTGCT-3’ |
| ***VEGFA*** | Forward | 5’-AGGGCAGAATCATCACGAAGT-3’ |
|  | Reverse | 5’-AGGGTCTCGATTGGATGGCA-3’ |
| ***VEGFC*** | Forward | 5’-GGCTGGCAACATAACAGAGAA-3’ |
|  | Reverse | 5’-CCCCACATCTATACACACCTCC-3’ |
